# Rh(III)-Catalyzed Double Annulation of 3-Phenyl-1,2,4-oxadiazoles with 2-Diazo-1,3-diketones: Access to Pyran-Fused Isoquinolines

**DOI:** 10.3390/molecules30010149

**Published:** 2025-01-02

**Authors:** Enshen Zhang, Mei Sun, Lvlv Gao

**Affiliations:** School of Chemistry and Materials Engineering, Huainan Normal University, Huainan 232038, China; zhes@hnnu.edu.cn (E.Z.); smbs@hnnu.edu.cn (M.S.)

**Keywords:** annulations, C-H bond activation, diazo compounds, oxadiazole, pyranoisoquinolines

## Abstract

Efficient access to pyranoisoquinoline derivatives via rhodium-catalyzed double C-H functionalization of phenyl oxadiazoles and diazo compounds has been developed. Two C-C bonds and one C-O and C-N bond formation was realized by this tandem reaction, along with the formation of two heterocycles, affording diversified pyran-fused isoquinolines in moderate to good yields with broad functional group tolerance under mild reaction conditions.

## 1. Introduction

**Figure 1:**
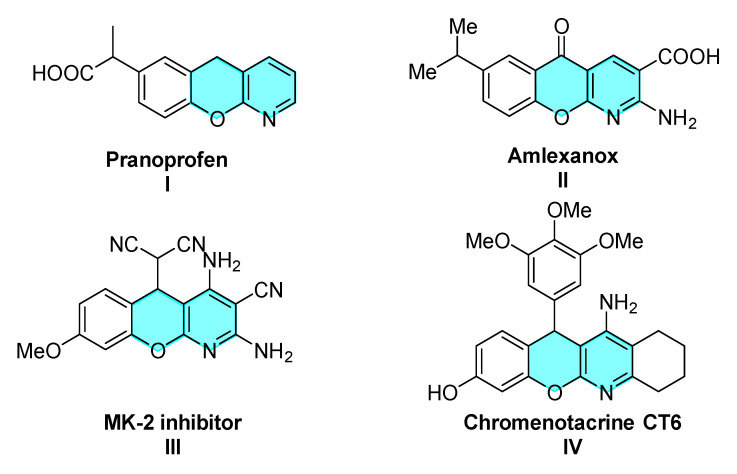
Representative molecules.

As a representative fused heterocyclic scaffold, pyranoisoquinoline derivatives are ubiquitous in bioactive compounds and natural products. For instance (Figure 1), Pranoprofen (I) and Amlexanox (II) are marketed anti-inflammatory drugs [1,2]. Compound III inhibits mitogen-activated protein kinase 2 (MK-2) and suppresses expression of TNFa in U937 cells [3]. Compound IV has potential activity for the treatment of Alzheimer’s disease [4].

**Scheme 1:**
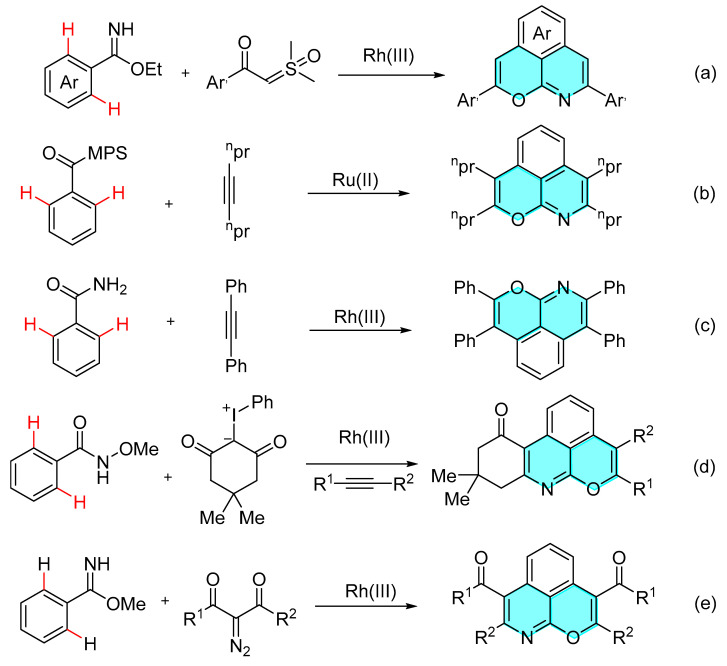
Different raw materials and catalysts of reported construction for chromopyridine skeleton. (**a**) Ethyl benzoate and α-aryl sulfoximines, Rh-catalyst. (**b**) Sulfoximines and alkyne compounds, Ru- catalyst. (**c**) Benzamide and arylacetylene, Rh-catalyst. (**d**) N-Methoxybenzamide, hypervalent iodonium ylides and alkyne, Rh-catalyst. (**e**) Methyl benzimidazolate and diazo compounds, Rh-catalyst.

Thus, the development of efficient methods for accessing such frameworks has attracted much more attention from the synthetic community. For example, in 2018, Cheng’s group developed a rhodium-catalyzed functionalization of aromatic C-H bonds to obtain a series of pyrano and isoquinoline derivatives with good optical properties [Scheme 1a] [5]. The group also developed an asymmetric double six-membered ring compound of heteroaryl and alkynes [Scheme 1b] [6]. You et al. reported a strategy for the synthesis of bicycles via Rh-catalyzed activation of double C-H bonds of benzamides [Scheme 1c] [7]. Maheswari developed a cascade strategy for the assembly of 3,4-dihydrophenanthridines using high-valent iodonium ylides as a carbene precursor [Scheme 1d] [8]. The yield of pyran-fused isoquinolines obtained via the reported literature methods is low and requires nitrogen protection, leading to the complex synthesis of pyran-fused isoquinolines. In 2022, Shang’s group reported the formation of a pyrano [4,3,2-ij]isoquinoline skeleton using bis-orthorhombic-C-H activation of methyl benzimidazolate and subsequent N,O-bicycloaddition with a-carbonyl diazides. However, AcCl, the raw material for the synthesis of methyl benzimidazolate, is highly toxic. The yields of pyran-fused isoquinolines synthesized by this reaction are generally low and the atom economy is poor [Scheme 1e] [9]. Therefore, it is necessary to further explore the low-toxicity and high-efficiency method to prepare pyran-fused isoquinolines. The unique physicochemical properties of pyranoisoquinolines have inspired more synthetic workers to develop new synthetic strategies and obtain molecules with diverse structures.

**Scheme 2:**
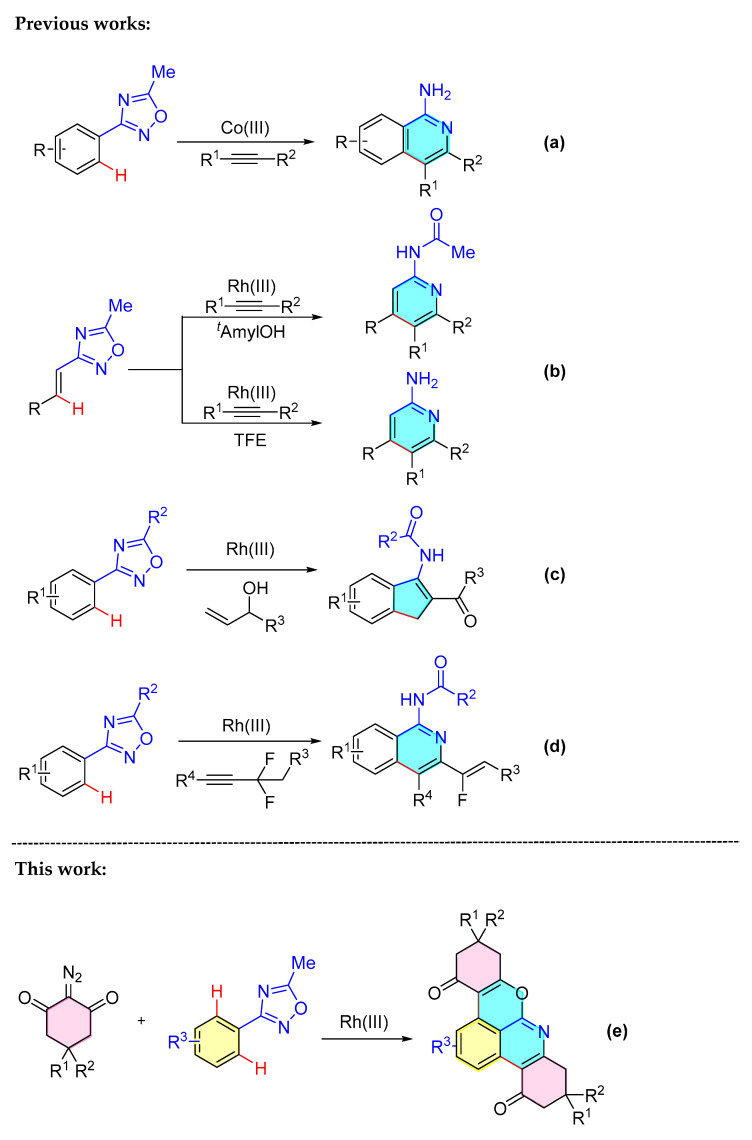
Transition-metal-catalyzed oxadiazole-directed C–H bond functionalization reactions. (**a**) Co-catalyzed reaction of oxadiazoles with alkynes. (**b**) Rh-catalyzed reaction of oxadiazoles with alkynes. (**c**) Rh-catalyzed reactions of oxadiazoles with allylic alcohols. (**d**) Rh-catalyzed reaction of oxadiazoles with difluoromethylene alkynes. (**e**) Rh-catalyzed bicyclisation of oxadiazoles with diazides.

Due to their unique structure, oxadiazoles are not only used as the cores of bioactive compounds as well as functional materials but are also applied as directing groups in the field of catalytic C-H bond activation. For example, in 2017, Zhu’s group used cobalt(III) to catalyze the construction of 1-aminoisoquinolines by oxadiazole [Scheme 2a] [10]. Later on, they reported a Rh(III)-catalyzed coupling of alkenyl oxadiazoles with alkynes for the synthesis of 2-acylamino acids and 2-aminopyridines, which are important heterocyclic backbones in a variety of natural products and synthetic drugs with readily reactive functional groups [Scheme 2b] [11]. In 2018, Dong and colleagues developed an efficient Rh(III)-catalyzed one-pot synthesis of indene derivatives from oxadiazole and allyl alcohols [Scheme 2c] [12]. In 2023, Dong’s group developed access to isoquinoline compounds with good chemo- and regioselectivity via Rh(III)-catalyzed [4+2] cycloaddition reactions of oxadiazoles and difluoromethylene alkynes [Scheme 2d] [13]. The reaction type of oxadiazole involved in the above report is a simple ortho-C-H activation reaction, which generally retains the unexpected structure and does not conform to the principle of atomic economy. Herein, we report a Rh-catalyzed double C-H activation of aryl oxadiazoles with diazo compounds. The reaction method is simple, the raw materials are easy to obtain, and it does not require N_2_ conditions. The yields of the prepared product are high. This reaction provides efficient access to obtain the pyranoisoquinoline skeleton [Scheme 2e].

## 2. Results and Discussion

This research was started with 2-diazo-5,5-dimethylcyclohexane-1,3-dione (**1a**) and 5-methyl-3-phenyl-1,2,4-oxadiazole (**2a**) as the model substrates (Table 1). A screening of the catalysts was carried out. First, the expected reaction to generate the chromopyridine product **3a** could be in [Cp*RhCl_2_]_2_ (5 mol %) as the catalyst (Table 1, entries 1–6). Other additives, including AgSbF_6_, AgOAc, AgBF_4_, and AgOTf, failed to promote the reaction, resulting in a significant decrease in reaction yield (Table 1, entries 7–10). Satisfactory yields of **3a** can also be obtained by reducing or increasing the content of additives (Table 1, entries 17 and 18). Common solvents such as toluene, acetonitrile, and DCE failed to improve product yields (Table 1, entries 12–14). For reaction temperatures of 90 °C and 110 °C, the isolated yields of product **3a** were 77% and 75%, respectively (Table 1, entries 19 and 20). No obvious change was revealed in the reaction after further optimization (Table 1, entries 21 and 22). The reaction yield was 39% when AgOAc was used as a base. The NaOAc and KOAc did not promote the reaction when used as bases (Table 1, entries 23–25). The reaction yield gradually increased with an increasing amount of Cu(OAc)_2_·H_2_O (Table 1, entries 26–28). The reaction yield was highest when the amount of base reached 2.0 equiv (entry 6), after which the reaction yield remained constant with an increasing amount (entry 29). The reaction yields gradually increased with the amount of AgNTf_2_ additive increasing (Table 1, entries 30–33). When the amount of AgNTf_2_ reached a certain amount, the reaction yield declined instead of increasing (Table 1, entry 34).

**Scheme 3:**
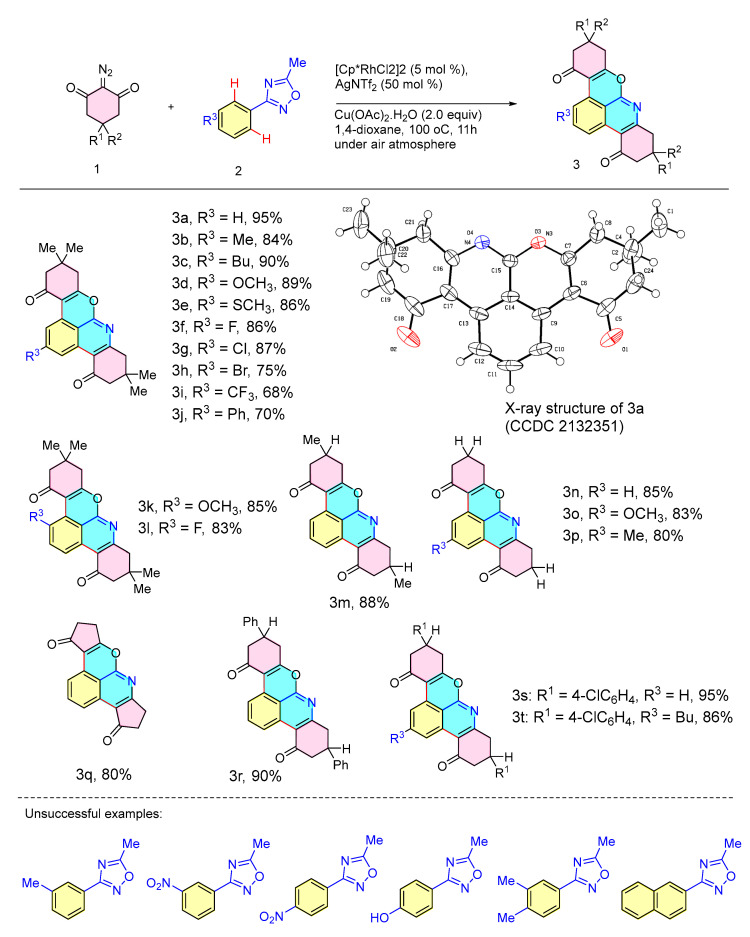
Substrate scope of cyclic 2-diazo-1,3-diketones with oxadiazole ^a,b^. ^a^ Reagents and conditions: cyclic 2-diazo compound **1** (0.35 mmol), oxadiazole **2** (0.1 mmol), [Cp*RhCl_2_]_2_ (5 mol %), AgNTf_2_ (50 mol %), and Cu(OAc)_2_·H_2_O (2.0 equiv) in 1,4-dioxane (3 mL) at 100 °C under air atmosphere for 11 h. ^b^ Isolated yields.

The interaction range of oxadiazole **2** with cyclic diazo compounds was examined under optimal conditions (Scheme 3). Typically, substrate **2** with both electron-donating (Me, Bu, OMe, and SMe) and electron-absorbing groups (F, Cl, and Br) was successfully converted to the desired chromopyridine products **3a**–**3h** in good yields (75–95%). When R^3^ was CF_3_ or Ph, the corresponding yields of products **3i** and **3j** were 68% and 70%, respectively. In addition, the reaction of oxadiazoles with cyclic diazonium compounds yielded **3k** and **3l** in 85% and 83% yields when the oxadiazole interstitials were linked to the substituted OMe and F. The reaction of different types of diazonium compounds with oxadiazole **2** also proceeded well, giving the target products **3m**–**3t** in 80–95% yields. Single-crystal characterization determined the structure of **3a** [14]. However, 3-phenyl-1,2,4-oxadiazole with other substituents could not give the desired product in this reaction. Due to the steric exclusion of the 3-phenyl-1,2,4-oxadiazole substituent group, the insertion of the diazo compound in the neighboring position was prevented, which is not conducive to the formation of Rh-carbene.

**Scheme 4:**
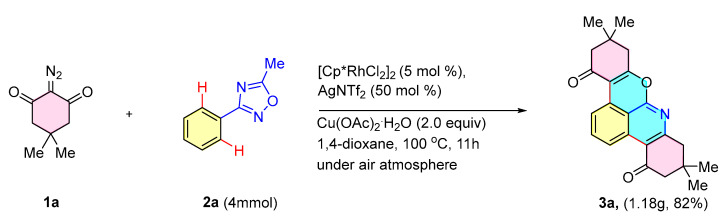
Gram-scale reaction.

**Scheme 5:**
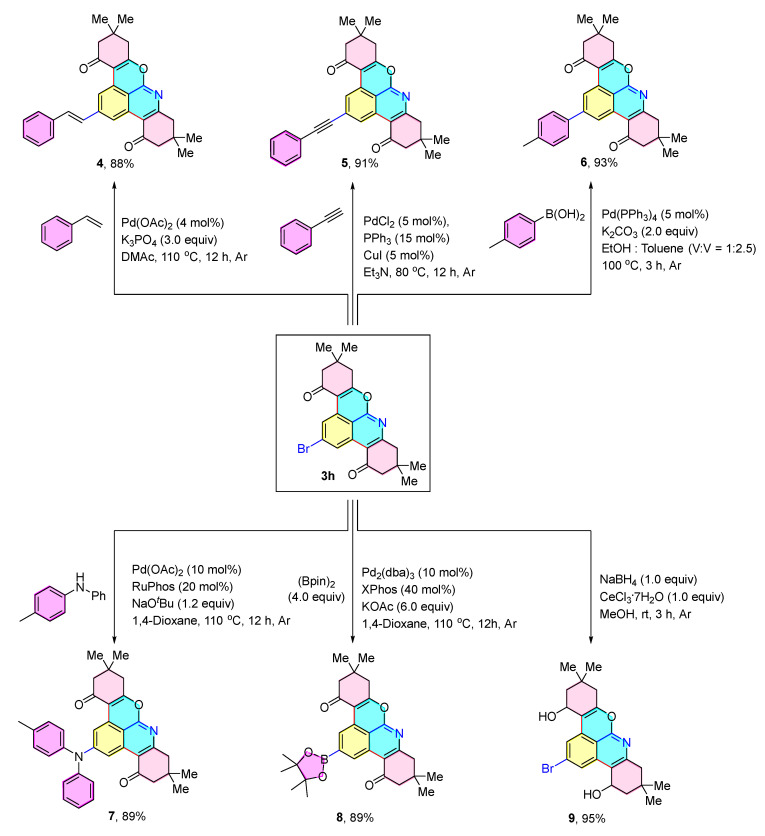
Examples of further synthetic transformations.

The reaction was scaled up to 4 mmol (scaled up to 40 times) to yield the desired product **3a** in 82% yield (Scheme 4). To demonstrate the utility of product **3**, its reactivity was then investigated in palladium-catalyzed cross-coupling reactions. As shown in the results in Scheme 5, the generated chromium pyridine compound **3h** reacted smoothly with styrene, phenylacetylene, phenylboronic acid, diphenylamine, and bis(pinacolato)diboron to obtain the corresponding products **4**, **5**, **6**, **7**, and **8** in 88%, 91%, 93%, 89%, and 89% yields, respectively. The generated chromium pyridine compound **3h** could also be reduced by NaBH_4_ to obtain product **9** in 95% yields.

Some of the products we prepared are partially similar in structure to those prepared by the previous Shang group, for example, products **3a** and **3b** [9]. Other products that they did not obtain include **3q**, **3r**, and **3s**. We were able to synthesis pyran-fused isoquinolines using cyclic diazides with different substituents. We report a Rh-catalyzed double C-H activation reaction of an aryl oxadiazole with a diazo compound. Compared with previous reports, our works have the advantages of easy availability of feedstock, no need to be carried out under nitrogen atmosphere, and high yield. Most of the synthesized compounds have bright fluorescence. The fluorescence emission range of these pyran-fused isoquinoline compounds is very wide, which indicates that they have a wide range of applications in biological imaging, organic light-emitting diodes, optoelectronic devices, fluorescent sensors, and biomedical imaging [5,6,9].

**Scheme 6:**
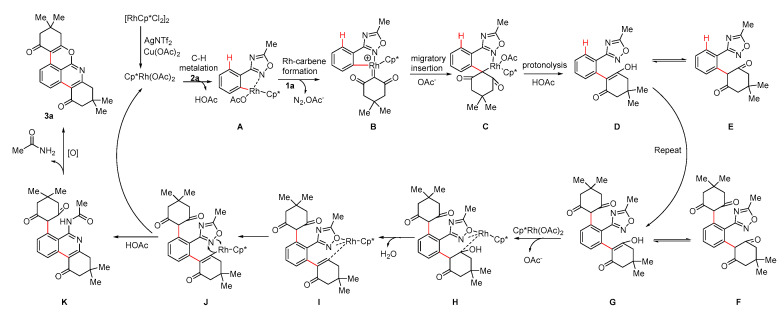
Proposed reaction mechanism. (A–C) Rhodium ring intermediate. (D) Unimolecular insertion product intermediate. (E) An isomer of D. (G) Bimolecular insertion intermediate. (F) An isomer of G. (H–J) Rhodium ring intermediates. (K) Unimolecular cyclisation and insertion intermediate.

Based on the experimental results and literature reports, a possible reaction mechanism was proposed in Scheme 6. Initially, [Cp*RhCl_2_]_2_ undergoes anion exchange in the presence of AgNTf_2_ and Cu(OAc)_2_·H_2_O to generate a Rh(III)-active catalyst. The active catalyst and substrate **2a** were activated by the C-H bond to form a five-membered rhodium ring as intermediate A. Subsequently, the cyclic 2-diazo-1,3-diketone 1a coordinates with intermediate A to form intermediate B by releasing N_2_. Then, migratory insertion of carbine into Rh-C bond completes the C-C coupling and affords the six-membered intermediate C, which can be protonated with acetic acid to generate intermediate D and release the Rh(III)-catalyst. Intermediate E is an isomer of intermediate D. Repeating the above steps, intermediate G was obtained. Intermediate F is an isomer of intermediate G. Intermediate G coordinates with the Rh(III)-active substance to generate intermediate H. Intermediate H undergoes an elimination reaction to eliminate H_2_O and obtain intermediate I. The Rh remains trivalent throughout and does not undergo a valence state change. Cu does not participate in the reaction in this process. Subsequently, intermediate I undergoes electron transfer to generate intermediate J [11,12,13]. In addition, complex J undergoes reductive elimination to form the C-N bond to generate complex K and generate Rh(I). Rh(I) is reoxidized to Rh(III) in the next catalytic cycle using the N-O bond cleavage as the internal oxidant [10,11,15]. Intermediate K undergoes oxidation by Cu(OAc)_2_·H_2_O followed by an intramolecular nucleophilic addition reaction to produce the desired product 3**a** [16].

## 3. Materials and Methods

### 3.1. General Methods (Chemistry)

The general methods are described in the Supplementary Materials. The 500 MHz NMR spectrometer (AVANCE IIITM HD 500), the 400 MHz NMR spectrometer (AVANCENEO 400) and the X-ray single crystal diffractometer (D8 VENTURE) are from Bruker, Germany. The company is located in Wurzbach in the Berlin region of eastern Germany. NMR analysis was carried out at 298 K. High Resolution Mass measurement was performed on Agilent QTOF 6520 mass spectrometer with electron spray ionization (ESI or APCI) as the ion source (Agilent Technologies, Santa Clara, CA, USA). The melting points were measured using SGWX-4 melting point apparatus and were not corrected (Zhejiang NADE Scientific Instrument Co., Ltd., Hangzhou, China). The software is ChemBioDraw Ultra (ACS Document 1996) and MestReNova (15.0).

### 3.2. General Procedures for the Preparation of Compounds ***3***

A Schlenk tube was equipped with a magnetic stir bar and charged with substituted 2-diazo-1,3-dione **1** (0.35 mmol), **2** (0.1 mmol), [Cp*RhCl_2_]_2_ (5 mol %), Cu(OAc)_2_·H_2_O (2.0 equiv), and AgNTf_2_ (0.5 equiv.), which were stirred in 1,4-dioxane (3.0 mL) under atmosphere at 100 °C for 11 h. After completion, the reaction mixture was purified by flash chromatography, eluting with ethyl acetate and petroleum ether to give product **3** as a yellow solid.

*6,6,11,11-tetramethyl-6,7,11,12-tetrahydrochromeno[2,3,4-gh]phenanthridine-4,13(5H,10H)-dione* (**3a**), a yellow solid after purification by flash column chromatography (petroleum ether/ethyl acetate = 10/1), 34.4 mg, yield: 95%; m.p 259–260 °C. ^1^H NMR (500 MHz, CDCl_3_) δ 9.20 (dd, *J* = 8.7, 0.9 Hz, 1H), 8.80 (dd, *J* = 7.7, 0.9 Hz, 1H), 7.86 (dd, *J* = 8.7, 7.7 Hz, 1H), 3.03 (s, 2H), 2.80 (s, 2H), 2.60 (s, 2H), 2.53 (s, 2H), 1.19 (s, 6H), 1.14 (s, 6H). ^13^C NMR (125 MHz, CDCl_3_) δ 199.4, 197.0, 168.0, 161.4, 159.8, 136.9, 136.0, 127.8, 123.5, 120.8, 117.6, 116.2, 112.2, 54.3, 53.1, 47.9, 42.7, 33.0, 32.3, 28.6, 28.5. HRMS (ESI) *m/z*: [M + H]^+^ calcd for C_23_H_24_NO_3_^+^ 362.1751; found 362.1753.

*2,6,6,11,11-pentamethyl-6,7,11,12-tetrahydrochromeno[2,3,4-gh]phenanthridine-4,13(5H,10H)-dione* (**3b**), a yellow solid after purification by flash column chromatography (petroleum ether/ethyl acetate = 10/1), 31.5 mg, yield: 84%; m.p 256–257 °C. ^1^H NMR (500 MHz, CDCl_3_) δ 9.03 (s, 1H), 8.65 (s, 1H), 3.04 (s, 2H), 2.78 (s, 2H), 2.59–2.58 (m, 5H), 2.52 (s, 2H), 1.18 (s, 6H), 1.13 (s, 6H). ^13^C NMR (125 MHz, CDCl_3_) δ 199.6, 197.1, 168.2, 161.7, 159.7, 147.5, 137.1, 127.6, 123.1, 122.3, 117.2, 114.4, 112.1, 54.3, 53.2, 47.9, 42.7, 32.9, 32.3, 28.6, 28.5, 23.8. HRMS (ESI) *m/z*: [M + H]^+^ calcd for C_24_H_26_NO_3_^+^ 376.1907; found 376.1906.

*2-butyl-6,6,11,11-tetramethyl-6,7,11,12-tetrahydrochromeno[2,3,4-gh]phenanthridine-4,13(5H,10H)-dione* (**3c**), a yellow solid after purification by flash column chromatography (petroleum ether/ethyl acetate = 10/1), 37.5 mg, yield: 90%; m.p 196–197 °C. ^1^H NMR (500 MHz, CDCl_3_) δ 9.02 (d, *J* = 1.5 Hz, 1H), 8.66 (d, *J* = 1.4 Hz, 1H), 2.99 (s, 2H), 2.86–2.78 (m, 2H), 2.77 (s, 2H), 2.58 (s, 2H), 2.51 (s, 2H), 1.74–1.59 (m, 2H), 1.43–1.35 (m, 2H), 1.18 (s, 6H), 1.13 (s, 6H), 0.93 (t, *J* = 7.4 Hz, 3H). ^13^C NMR (125 MHz, CDCl_3_) δ 199.6, 197.2, 168.1, 161.7, 159.7, 152.4, 137.1, 127.6, 122.6, 121.8, 117.3, 114.6, 112.2, 54.3, 53.2, 48.0, 42.7, 37.8, 34.0, 33.0, 32.3, 28.6, 28.5, 23.0, 14.4. HRMS (ESI) *m/z*: [M + H]^+^ calcd for C_27_H_32_NO_3_^+^ 418.2377; found 418.2368.

*2-methoxy-6,6,11,11-tetramethyl-6,7,11,12-tetrahydrochromeno[2,3,4-gh]phenanthridine-4,13(5H,10H)-dione* (**3d**), a yellow solid after purification by flash column chromatography (petroleum ether/ethyl acetate = 10/1), 34.8 mg, yield: 89%; m.p 260–261 °C. ^1^H NMR (500 MHz, CDCl_3_) δ 8.70 (d, *J* = 2.4 Hz, 1H), 8.41 (d, *J* = 2.4 Hz, 1H), 3.96 (s, 3H), 2.98 (s, 2H), 2.77 (s, 2H), 2.58 (s, 2H), 2.51 (s, 2H), 1.18 (s, 6H), 1.12 (s, 6H). ^13^C NMR (125 MHz, CDCl_3_) δ 199.6, 196.9, 168.5, 166.1, 162.6, 159.2, 139.3, 129.4, 116.8, 111.7, 111.5, 111.3, 104.0, 56.1, 54.3, 53.0, 48.0, 42.7, 32.9, 32.3, 28.6, 28.5. HRMS (ESI) *m/z*: [M + H]^+^ calcd for C_24_H_26_NO_4_^+^ 392.1856; found 392.1853.

*6,6,11,11-tetramethyl-2-(methylthio)-6,7,11,12-tetrahydrochromeno[2,3,4-gh]phenanthridine-4,13(5H,10H)-dione* (**3e**), a yellow solid after purification by flash column chromatography (petroleum ether/ethyl acetate = 10/1), 35.1 mg, yield: 86%; m.p 305–306 °C. ^1^H NMR (400 MHz, CDCl_3_) δ 9.04 (d, *J* = 1.5 Hz, 1H), 8.72 (d, *J* = 1.5 Hz, 1H), 3.00 (s, 2H), 2.79 (s, 2H), 2.62 (s, 3H), 2.59 (s, 2H), 2.52 (s, 2H), 1.19 (s, 6H), 1.14 (s, 6H). ^13^C NMR (100 MHz, CDCl_3_) δ 199.1, 196.5, 168.3, 162.0, 150.5, 136.6, 127.0, 117.8, 116.6, 115.9, 112.9, 111.1, 53.9, 52.7, 47.6, 42.4, 32.5, 31.9, 28.1, 28.0, 14.7. HRMS (ESI) *m/z*: [M + H]^+^ calcd for C_24_H_26_NO_3_S^+^ 408.1628; found 408.1625.

*2-fluoro-6,6,11,11-tetramethyl-6,7,11,12-tetrahydrochromeno[2,3,4-gh]phenanthridine-4,13(5H,10H)-dione* (**3f**), a yellow solid after purification by flash column chromatography (petroleum ether/ethyl acetate = 10/1), 32.6 mg, yield: 86%; m.p 235–236 °C. ^1^H NMR (500 MHz, CDCl_3_) δ 8.94 (dd, *J* = 11.9, 2.5 Hz, 1H), 8.62 (dd, *J* = 10.6, 2.5 Hz, 1H), 3.03 (s, 2H), 2.82 (s, 2H), 2.60 (s, 2H), 2.54 (s, 2H), 1.20 (s, 6H), 1.14 (s, 6H). ^13^C NMR (100 MHz, CDCl_3_) δ198.8, 196.2, 168.7 (d, *J*_C,F_ = 27.0 Hz), 166.4, 162.3, 138.8, 130.5 (d, *J*_C,F_ = 13.7 Hz), 117.1, 113.1, 110.2 (d, *J*_C,F_ = 29.4 Hz), 108.7, 108.4, 53.7, 52.5, 47.5, 42.3, 32.6, 32.0, 28.2, 28.1. HRMS (ESI) *m/z*: [M + H]^+^ calcd for C_23_H_23_FNO_3_^+^ 380.1656; found 380.1665.

*2-chloro-6,6,11,11-tetramethyl-6,7,11,12-tetrahydrochromeno[2,3,4-gh]phenanthridine-4,13(5H,10H)-dione* (**3g**), a yellow solid after purification by flash column chromatography (petroleum ether/ethyl acetate = 10/1), 34.4 mg, yield: 87%; m.p 234–235 °C. ^1^H NMR (500 MHz, CDCl_3_) δ 9.27 (d, *J* = 1.9 Hz, 1H), 8.84 (d, *J* = 1.9 Hz, 1H), 3.03 (s, 2H), 2.81 (s, 2H), 2.60 (s, 2H), 2.53 (s, 2H), 1.19 (s, 6H), 1.14 (s, 6H). ^13^C NMR (125 MHz, CDCl_3_) δ 199.1, 196.6, 168.8, 162.5, 159.5, 143.6, 137.6, 129.2, 122.9, 121.5, 116.8, 114.4, 111.3, 54.1, 52.9, 47.9, 42.7, 32.9, 32.3, 28.6, 28.5. HRMS (ESI) *m/z*: [M + H]^+^ calcd for C_23_H_23_ClNO_3_^+^ 396.1361; found 396.1369.

*2-bromo-6,6,11,11-tetramethyl-6,7,11,12-tetrahydrochromeno[2,3,4-gh]phenanthridine-4,13(5H,10H)-dione* (**3h**), a yellow solid after purification by flash column chromatography (petroleum ether/ethyl acetate = 10/1), 32.9 mg, yield: 75%; m.p 259–260 °C. ^1^H NMR (500 MHz, CDCl_3_) δ 9.43 (s, 1H), 8.97 (s, 1H), 3.03 (s, 2H), 2.81 (s, 2H), 2.60 (s, 2H), 2.53 (s, 2H), 1.19 (s, 6H), 1.14 (s, 6H). ^13^C NMR (100 MHz, CDCl_3_) δ 198.7, 196.2, 168.5, 166.3, 162.2, 159.0, 130.4, 117.0, 113.0, 111.2, 110.2, 110.0, 108.7, 108.4, 53.7, 52.5, 47.5, 42.3, 32.5, 31.9, 28.2, 28.1. HRMS (ESI) *m/z*: [M + H]^+^ calcd for C_23_H_23_BrNO_3_^+^ 440.0856; found 440.0853.

*6,6,11,11-tetramethyl-2-(trifluoromethyl)-6,7,11,12-tetrahydrochromeno[2,3,4-gh]phenanthridine-4,13(5H,10H)-dione* (**3i**), a yellow solid after purification by flash column chromatography (petroleum ether/ethyl acetate = 10/1), 29.2 mg, yield: 68%; m.p 240–241 °C. ^1^H NMR (400 MHz, CDCl_3_) δ 9.56 (dd, *J* = 1.7, 0.8 Hz, 1H), 9.08 (d, *J* = 1.6 Hz, 1H), 3.07 (s, 2H), 2.83 (s, 2H), 2.63 (s, 2H), 2.56 (s, 2H), 1.21 (s, 6H), 1.15 (s, 6H). ^13^C NMR (125 MHz, CDCl_3_) δ 198.7, 196.1, 168.5, 162.0, 159.0, 136.2, 128.7, 127.8, 120.3 (q, *J*_C,F_ = 5.6 Hz), 117.5, 116.5 (q, *J*_C,F_ = 3.8 Hz), 111.2, 53.7, 52.5, 47.4, 42.3, 32.6, 31.9, 28.1, 28.0. HRMS (ESI) *m/z*: [M + H]^+^ calcd for C_24_H_23_F_3_NO_3_^+^ 430.1625; found 430.1624.

*6,6,11,11-tetramethyl-2-phenyl-6,7,11,12-tetrahydrochromeno[2,3,4-gh]phenanthridine-4,13(5H,10H)-dione* (**3j**), a yellow solid after purification by flash column chromatography (petroleum ether/ethyl acetate = 10/1), 30.6 mg, yield: 70%; m.p 280–281 °C. ^1^H NMR (400 MHz, CDCl_3_) δ 9.51 (d, *J* = 1.6 Hz, 1H), 9.14 (d, *J* = 1.6 Hz, 1H), 7.82–7.79 (m, 2H), 7.51–7.47 (m, 2H), 7.44–7.40 (m, 1H), 3.04 (s, 2H), 2.81 (s, 2H), 2.62 (s, 2H), 2.54 (s, 2H), 1.20 (s, 6H), 1.16 (s, 6H). ^13^C NMR (100 MHz, CDCl_3_) δ 199.0, 196.6, 167.9, 161.5, 159.3, 148.1, 140.5, 136.9, 128.9, 128.5, 128.0, 127.8, 121.2, 119.6, 117.3, 114.7, 111.8, 53.9, 52.7, 47.6, 42.3, 32.6, 31.9, 28.2, 28.1. HRMS (ESI) *m/z*: [M + H]^+^ calcd for C_29_H_28_NO_3_^+^ 438.2064; found 438.2066.

*3-methoxy-6,6,11,11-tetramethyl-6,7,11,12-tetrahydrochromeno[2,3,4-gh]phenanthridine-4,13(5H,10H)-dione* (**3k**), a yellow solid after purification by flash column chromatography (petroleum ether/ethyl acetate = 10/1), 33.3 mg, yield: 85%; m.p 258–259 °C. ^1^H NMR (500 MHz, CDCl_3_) δ 8.78 (d, *J* = 8.6 Hz, 1H), 7.29 (d, *J* = 8.6 Hz, 1H), 3.96 (s, 3H), 2.96 (s, 2H), 2.75 (s, 2H), 2.69 (s, 2H), 2.50 (s, 2H), 1.18 (s, 6H), 1.16 (s, 6H). ^13^C NMR (125 MHz, CDCl_3_) δ 197.2, 197.0, 166.4, 159.3, 158.2, 153.3, 126.6, 122.0, 121.5, 120.2, 118.1, 115.7, 112.0, 56.5, 54.0, 53.0, 47.4, 42.5, 34.2, 32.4, 29.2, 28.6. HRMS (ESI) *m/z*: [M + H]^+^ calcd for C_24_H_26_NO_4_^+^ 392.1856; found 392.1857.

*3-fluoro-6,6,11,11-tetramethyl-6,7,11,12-tetrahydrochromeno[2,3,4-gh]phenanthridine-4,13(5H,10H)-dione* (**3l**), a yellow solid after purification by flash column chromatography (petroleum ether/ethyl acetate = 10/1), 31.5 mg, yield: 83%; m.p 234–235 °C. ^1^H NMR (500 MHz, CDCl_3_) δ 9.43 (s, 1H), 9.97 (s, 1H), 3.03 (s, 2H), 2.81 (s, 2H), 2.60 (s, 2H), 2.53 (s, 2H), 1.19 (s, 6H), 1.14 (s, 6H). ^13^C NMR (125 MHz, CDCl_3_) δ 196.8, 196.3, 167.4, 159.5 (d, *J*_C,F_ = 58.8 Hz), 156.6, 154.6, 126.2, 124.6 (t, *J*_C,F_ = 12.5 Hz), 122.1 (d, *J*_C,F_ = 7.8 Hz), 121.5 (d, *J*_C,F_ = 22.5 Hz), 119.4, 118.3, 117.6, 111.8, 53.7, 53.0, 47.6, 42.5, 34.0, 32.3, 28.9, 28.6. HRMS (ESI) *m/z*: [M + H]^+^ calcd for C_23_H_23_FNO_3_^+^ 380.1656; found 380.1658.

*6,11-dimethyl-6,7,11,12-tetrahydrochromeno[2,3,4-gh]phenanthridine-4,13(5H,10H)-dione* (**3m**), a yellow solid after purification by flash column chromatography (petroleum ether/ethyl acetate = 10/1), 29.3 mg, yield: 88%; m.p 241–242 °C. ^1^H NMR (500 MHz, CDCl_3_) δ 9.19 (d, *J* = 8.6 Hz, 1H), 8.81 (d, *J* = 7.4 Hz, 1H), 7.85 (t, *J* = 8.2 Hz, 1H), 3.27–3.08 (m, 1H), 3.02–2.91 (m, 1H), 2.92–2.77 (m, 2H), 2.76–2.60 (m, 2H), 2.55–2.27 (m, 4H), 1.21–1.18 (m, 6H). ^13^C NMR (125 MHz, CDCl_3_) δ 199.4, 197.0, 169.0, 162.4, 159.4, 137.0, 136.0, 127.9, 123.6, 120.9, 118.2, 116.2, 112.8, 48.8, 47.4, 42.3, 37.0, 29.3, 28.0, 21.5, 21.2. HRMS (ESI) *m/z*: [M + H]^+^ calcd for C_21_H_20_NO_3_^+^ 334.1438; found 334.1447.

*6,7,11,12-tetrahydrochromeno[2,3,4-gh]phenanthridine-4,13(5H,10H)-dione* (**3n**), a yellow solid after purification by flash column chromatography (petroleum ether/ethyl acetate = 10/1), 25.9 mg, yield: 85%; m.p 239–240 °C. ^1^H NMR (500 MHz, CDCl_3_) δ 9.19 (d, *J* = 8.7 Hz, 1H), 8.81 (d, *J* = 7.7 Hz, 1H), 7.85 (dd, *J* = 8.6, 7.8 Hz, 1H), 3.15 (t, *J* = 6.2 Hz, 2H), 2.95 (t, *J* = 6.3 Hz, 2H), 2.83–2.71 (m, 2H), 2.70–2.57 (m, 2H), 2.37–1.99 (m, 4H). ^13^C NMR (125 MHz, CDCl_3_) δ 199.4, 197.0, 169.5, 163.0, 159.2, 137.7, 136.0, 128.0, 123.7, 121.1, 118.6, 116.2, 113.2, 40.7, 39.2, 34.1, 29.2, 22.0, 20.4. HRMS (ESI) *m/z*: [M + H]^+^ calcd for C_19_H_16_NO_3_^+^ 306.1125; found 306.1134.

*2-methoxy-6,7,11,12-tetrahydrochromeno[2,3,4-gh]phenanthridine-4,13(5H,10H)-dione* (**3o**), a yellow solid after purification by flash column chromatography (petroleum ether/ethyl acetate = 10/1), 27.8 mg, yield: 83%; m.p 309–310 °C. ^1^H NMR (400 MHz, CDCl_3_) δ 8.74 (d, *J* = 2.4 Hz, 1H), 8.46 (d, *J* = 2.4 Hz, 1H), 3.98 (s, 3H), 3.12 (t, *J* = 6.2 Hz, 2H), 2.94 (t, *J* = 6.3 Hz, 2H), 2.75–2.72 (m, 2H), 2.68–2.64 (m, 2H), 2.21–2.16 (m, 4H). ^13^C NMR (100 MHz, CDCl_3_) δ 199.2, 196.5, 169.6, 165.7, 163.8, 158.3, 139.2, 129.2, 117.4, 112.3, 111.4, 111.0, 104.0, 55.7, 40.3, 38.8, 33.9, 28.8, 21.7, 19.9. HRMS (ESI) *m/z*: [M + H]^+^ calcd for C_20_H_18_NO_4_^+^ 336.1230; found 336.1232.

*2-methyl-6,7,11,12-tetrahydrochromeno[2,3,4-gh]phenanthridine-4,13(5H,10H)-dione* (**3p**), a yellow solid after purification by flash column chromatography (petroleum ether/ethyl acetate = 10/1), 25.5 mg, yield: 80%; m.p 328–329 °C. ^1^H NMR (400 MHz, CDCl_3_) δ 8.99 (s, 1H), 8.63 (s, 1H), 3.12 (t, *J* = 6.4 Hz, 2H), 2.93 (t, *J* = 6.4 Hz, 2H), 2.73 (t, *J* = 6.4 Hz, 2H), 2.66 (t, *J* = 6.4 Hz, 2H), 2.57 (s, 3H), 2.20–2.17 (m, 4H). ^13^C NMR (100 MHz, CDCl_3_) δ 199.1, 196.7, 169.2, 162.9, 158.7, 147.0, 136.9, 127.2, 122.8, 122.1, 117.7, 114.0, 112.6, 40.3, 38.9, 33.7, 28.8, 23.4, 21.6, 19.9. HRMS (ESI) *m/z*: [M + H]^+^ calcd for C_20_H_18_NO_3_^+^ 320.1281; found 320.1286.

*5,6,9,10-tetrahydrocyclopenta[5,6]pyrano[4,3,2-ij]cyclopenta[c]isoquinoline-4,11-Dione* (**3q**), a yellow solid after purification by flash column chromatography (petroleum ether/ethyl acetate = 10/1), 22.2 mg, yield: 80%; m.p 214–215 °C. ^1^H NMR (500 MHz, CDCl_3_) δ 8.62 (d, *J* = 8.3 Hz, 1H), 8.19 (d, *J* = 7.3 Hz, 1H), 7.86 (t, *J* = 7.9 Hz, 1H), 3.44–3.14 (m, 2H), 3.17–2.90 (m, 2H), 2.85–2.80 (m, 2H), 2.80–2.75 (m, 2H). ^13^C NMR (125 MHz, CDCl_3_) δ 204.7, 200.5, 180.9, 174.6, 163.3, 136.0, 134.9, 126.8, 121.7, 121.4, 118.8, 117.0, 115.5, 36.5, 34.9, 29.2, 26.0. HRMS (ESI) *m/z*: [M + H]^+^ calcd for C_17_H_12_NO_3_^+^ 278.0812; found 278.0816.

*6,11-diphenyl-6,7,11,12-tetrahydrochromeno[2,3,4-gh]phenanthridine-4,13(5H,10H)-dione* (**3r**), a yellow solid after purification by flash column chromatography (petroleum ether/ethyl acetate = 10/1), 41.1 mg, yield: 90%; m.p 263–264 °C. ^1^H NMR (500 MHz, CDCl_3_) δ 9.26 (d, *J* = 8.6 Hz, 1H), 8.88 (d, *J* = 7.7 Hz, 1H), 7.91 (t, *J* = 8.2 Hz, 1H), 7.42–7.38 (m, 4H), 7.36–7.29 (m, 6H), 3.59 (d, *J* = 14.8 Hz, 2H), 3.51–3.28 (m, 2H), 3.19 (d, *J* = 8.0 Hz, 2H), 3.11–3.02 (m, 1H), 3.02–2.76 (m, 3H). ^13^C NMR (125 MHz, CDCl_3_) δ 198.1, 195.7, 168.2, 161.8, 159.2, 142.6, 141.4, 136.6, 135.8, 129.1, 128.9, 127.5, 127.4, 127.1, 126.7, 126.6, 123.4, 120.8, 117.9, 112.6, 47.1, 45.8, 41.0, 39.2, 37.9, 36.0. HRMS (ESI) *m/z*: [M + H]^+^ calcd for C_31_H_24_NO_3_^+^ 458.1751; found 458.1744.

*6,11-bis(4-chlorophenyl)-6,7,11,12-tetrahydrochromeno[2,3,4-gh]phenanthridine-4,13(5H,10H)-dione* (**3s**), a yellow solid after purification by flash column chromatography (petroleum ether/ethyl acetate = 10/1), 49.9 mg, yield: 95%; m.p 329–330 °C. ^1^H NMR (500 MHz, CDCl_3_) δ 9.24 (d, *J* = 8.7 Hz, 1H), 8.86 (d, *J* = 7.7 Hz, 1H), 7.90 (t, *J* = 8.2 Hz, 1H), 7.38–7.34 (m, 4H), 7.27–7.24 (m, 4H), 3.58–3.55 (m, 2H), 3.41–3.33 (m, 2H), 3.16–3.13 (m, 2H), 3.03 (d, *J* = 13.5 Hz, 1H), 2.96–2.85 (m, 3H). ^13^C NMR (125 MHz, CDCl_3_) δ 198.0, 195.6, 168.3, 161.8, 159.5, 141.5, 140.2, 137.0, 136.3, 133.8, 133.2, 129.7, 129.4, 128.5, 128.4, 127.7, 123.8, 121.3, 118.3, 116.3, 113.1, 47.3, 46.0, 41.3, 39.0, 37.7, 36.4. HRMS (ESI) *m/z*: [M + H]^+^ calcd for C_31_H_22_ClNO_3_^+^ 526.0971; found 526.0978.

*2-butyl-6,11-bis(4-chlorophenyl)-6,7,11,12-tetrahydrochromeno[2,3,4-gh]phenanthridine-4,13(5H,10H)-dione* (**3t**), a yellow solid after purification by flash column chromatography (petroleum ether/ethyl acetate = 10/1), 50.0 mg, yield: 86%; m.p 230–231 °C. ^1^H NMR (500 MHz, CDCl_3_) δ 9.08 (s, 1H), 8.73 (d, *J* = 1.4 Hz, 1H), 7.35 (dd, *J* = 14.3, 8.5 Hz, 4H), 7.26 (s, 1H), 7.25–7.23 (m, 3H), 3.66–3.50 (m, 2H), 3.43–3.24 (m, 2H), 3.21–3.06 (m, 2H), 3.08–2.98 (m, 1H), 2.97–2.73 (m, 5H), 1.74–1.68 (m, 2H), 1.43–1.39 (m, 2H), 0.95 (t, *J* = 7.3 Hz, 3H). ^13^C NMR (100 MHz, CDCl_3_) δ 197.8, 195.4, 168.0, 161.6, 159.1, 152.4, 141.2, 139.8, 136.9, 133.4, 132.8, 129.3, 129.0, 128.1, 128.0, 127.1, 122.7, 122.0, 117.6, 114.4, 112.7, 47.0, 45.7, 41.0, 38.7, 37.4, 37.4, 36.0, 33.6, 22.6, 14.0. HRMS (ESI) *m/z*: [M + H]^+^ calcd for C_35_H_30_ClNO_3_^+^ 582.1597; found 582.1604.

### 3.3. General Procedures for the Preparation of Compounds ***4***

A Schlenk tube was equipped with a magnetic stir bar and charged with 2-bromo-6,6,11,11-tetramethyl-6,7,11,12-tetrahydrochromeno[2,3,4-gh]phenanthridine-4,13(5H,10H)-dione **3h** (0.1 mmol), styrene (0.1 mmol), Pd(OAc)_2_ (4 mol %), K_3_PO_4_ (3.0 equiv), and DMAc (2 mL). Then, the flask was sealed under Ar and stirred at 110 °C for 12 h. After the reaction was quenched by the addition of water, the mixture was extracted with dichloromethane, and the combined organic layer was dried over sodium sulfate. The concentration in vacuo followed by silica gel column purification with petroleum ether/ethyl acetate eluent gave the desired product **4**.

*(E)-6,6,11,11-tetramethyl-2-styryl-6,7,11,12-tetrahydrochromeno[2,3,4-gh] phenanthridine-4,13(5H,10H)-dione* (**4**), a yellow solid after purification by flash column chromatography (petroleum ether/ethyl acetate = 10/1), 40.7 mg, yield: 88%; m.p 186–187 °C. ^1^H NMR (400 MHz, CDCl_3_) δ 9.12 (s, 1H), 8.70 (d, *J* = 1.2 Hz, 1H), 7.25–7.13 (m, 5H), 6.88–6.71 (m, 2H), 2.99 (s, 2H), 2.75 (s, 2H), 2.55 (s, 2H), 2.44 (s, 2H), 1.15 (s, 6H), 1.12 (s, 6H). ^13^C NMR (100 MHz, CDCl_3_) δ 198.6, 195.9, 167.4, 161.2, 159.2, 145.1, 136.6, 136.5, 133.3, 130.2, 129.0, 128.2, 127.5, 127.0, 123.3, 121.2, 117.1, 114.6, 111.7, 53.8, 52.6, 47.5, 42.3, 32.5, 31.8, 28.1, 28.0. HRMS (ESI) *m/z*: [M + H]^+^ calcd for C_31_H_30_NO_3_^+^ 464.2220; found 464.2213.

### 3.4. General Procedures for the Preparation of Compounds ***5***

A Schlenk tube was equipped with a magnetic stir bar and charged with 2-bromo-6,6,11,11-tetramethyl-6,7,11,12-tetrahydrochromeno[2,3,4-gh]phenanthridine-4,13(5H,10H)-dione **3h** (0.1 mmol), ethynylbenzene (0.1 mmol), PdCl_2_ (5 mol %), PPh_3_ (15 mol %), CuI (5 mol %), and Et_3_N (2 mL). Then, the flask was sealed under Ar and stirred at 80 °C for 12 h. After the reaction was quenched by the addition of water, the mixture was extracted with dichloromethane, and the combined organic layer was dried over sodium sulfate. The concentration in vacuo followed by silica gel column purification with petroleum ether/ethyl acetate eluent gave the desired product **5**.

*6,6,11,11-tetramethyl-2-(phenylethynyl)-6,7,11,12-tetrahydrochromeno[2,3,4-gh] phenanthridine-4,13(5H,10H)-dione* (**5**), a yellow solid after purification by flash column chromatography (petroleum ether/ethyl acetate = 10/1), 41.9 mg, yield: 91%; m.p 222–223 °C. ^1^H NMR (500 MHz, CDCl_3_) δ 9.40 (s, 1H), 8.94 (s, 1H), 7.74–7.53 (m, 2H), 7.50–7.30 (m, 3H), 3.03 (s, 2H), 2.80 (s, 2H), 2.62 (s, 2H), 2.55 (s, 2H), 1.20 (s, 6H), 1.15 (s, 6H). ^13^C NMR (125 MHz, CDCl_3_) δ 198. 8, 196.4, 168.0, 161.7, 159.1, 136.3, 132.0, 130.9, 128.8, 128.4, 127.4, 126.2, 122.9, 122.7, 116.7, 114.7, 111.3, 93.1, 89.8, 53.8, 52.6, 47.5, 42.3, 32.6, 31.9, 28.2, 28.1. HRMS (ESI) *m/z*: [M + H]^+^ calcd for C_31_H_28_NO_3_^+^ 462.2064; found 462.2067.

### 3.5. General Procedures for the Preparation of Compounds ***6***

A Schlenk tube was equipped with a magnetic stir bar and charged with 2-bromo-6,6,11,11-tetramethyl-6,7,11,12-tetrahydrochromeno[2,3,4-gh]phenanthridine-4,13(5H,10H)-dione **3h** (0.1 mmol), p-tolylboronic acid (0.1 mmol), Pd(PPh_3_)_4_ (5 mol %), K_2_CO_3_ (2.0 equiv), and EtOH:Toluene (V:V = 1:2.5, 3.5 mL). Then, the flask was sealed under Ar and stirred at 100 °C for 3 h. After the reaction was quenched by the addition of water, the mixture was extracted with dichloromethane, and the combined organic layer was dried over sodium sulfate. The concentration in vacuo followed by silica gel column purification with petroleum ether/ethyl acetate eluent gave the desired product **6**.

*6,6,11,11-tetramethyl-2-(p-tolyl)-6,7,11,12-tetrahydrochromeno[2,3,4-gh] phenanthridine-4,13(5H,10H)-dione* (**6**), a yellow solid after purification by flash column chromatography (petroleum ether/ethyl acetate = 10/1), 41.9 mg, yield: 93%; m.p 143–144 °C. ^1^H NMR (500 MHz, CDCl_3_) δ 9.51 (s, 1H), 9.15 (s, 1H), 7.73 (d, *J* = 7.5 Hz, 2H), 7.30 (d, *J* = 7.5 Hz, 2H), 3.04 (s, 2H), 2.81 (s, 2H), 2.62 (s, 2H), 2.55 (s, 2H), 2.43 (s, 3H), 1.21 (s, 6H), 1.16 (s, 6H). ^13^C NMR (125 MHz, CDCl_3_) δ 199.1, 196.6, 167.9, 161.5, 159.4, 148.1, 138.6, 137.5, 137.0, 134.7, 129.6, 127.9, 120.8, 119.5, 117.3, 114.6, 111.9, 53.9, 52.8, 47.6, 42.4, 32.6, 31.9, 28.2, 28.1, 21.2. HRMS (ESI) *m/z*: [M + H]^+^ calcd for C_30_H_30_NO_3_^+^ 452.2220; found 452.2227.

### 3.6. General Procedures for the Preparation of Compounds ***7***

A Schlenk tube was equipped with a magnetic stir bar and charged with 2-bromo-6,6,11,11-tetramethyl-6,7,11,12-tetrahydrochromeno[2,3,4-gh]phenanthridine-4,13(5H,10H)-dione **3h** (0.1 mmol), 4-methyl-N-phenylaniline (0.1 mmol), Pd(OAc)_2_ (10 mol %), RuPhos (20 mol %), NaO*^t^*Bu (1.2 equiv), and 1,4-Dioxane (2 mL). Then, the flask was sealed under Ar and stirred at 110 °C for 12 h. After the reaction was quenched by the addition of water, the mixture was extracted with dichloromethane, and the combined organic layer was dried over sodium sulfate. The concentration in vacuo followed by silica gel column purification with petroleum ether/ethyl acetate eluent gave the desired product **7**.

*6,6,11,11-tetramethyl-2-(phenyl(p-tolyl)amino)-6,7,11,12-tetrahydrochromeno[2,3,4-gh] phenanthridine-4,13(5H,10H)-dione* (**7**), a yellow solid after purification by flash column chromatography (petroleum ether/ethyl acetate = 10/1), 48.2 mg, yield: 89%; m.p 199–200 °C. ^1^H NMR (500 MHz, CDCl_3_) δ 8.68 (s, 1H), 8.46 (s, 1H), 7.37 (t, *J* = 7.5 Hz, 2H), 7.24–7.16 (m, 5H), 7.12 (d, *J* = 8.0 Hz, 2H), 2.92 (s, 2H), 2.74 (s, 2H), 2.46 (s, 2H), 2.41 (s, 2H), 2.36 (s, 3H), 1.14 (s, 6H), 1.09 (s, 6H). ^13^C NMR (125 MHz, CDCl_3_) δ 198.7, 196.5, 167.9, 162.2, 158.7, 154.7, 146.2, 143.4, 138.0, 135.3, 130.4, 129.7, 128.3, 126.6, 125.4, 115.9, 112.9, 111.5, 110.2, 110.0, 53.8, 52.6, 47.7, 42.4, 32.4, 31.9, 28.2, 28.1, 21.1. HRMS (ESI) *m/z*: [M + H]^+^ calcd for C_36_H_35_N_2_O_3_^+^ 543.2642; found 543.2648.

### 3.7. General Procedures for the Preparation of Compounds ***8***

A Schlenk tube was equipped with a magnetic stir bar and charged with 2-bromo-6,6,11,11-tetramethyl-6,7,11,12-tetrahydrochromeno[2,3,4-gh]phenanthridine-4,13(5H,10H)-dione **3h** (0.1 mmol), (Bpin)_2_ (4.0 equiv), Pd_2_(dba)_3_ (10 mol %), XPhos (40 mol %), KOAc (6.0 equiv), and 1,4-Dioxane (2 mL). Then, the flask was sealed under Ar and stirred at 110 °C for 12 h. After the reaction was quenched by the addition of water, the mixture was extracted with dichloromethane, and the combined organic layer was dried over sodium sulfate. The concentration in vacuo followed by silica gel column purification with petroleum ether/ethyl acetate eluent gave the desired product **8**.

*6,6,11,11-tetramethyl-2-(4,4,5,5-tetramethyl-1,3,2-dioxaborolan-2-yl)-6,7,11,12-tetrahydrochromeno[2,3,4-gh] phenanthridine-4,13(5H,10H)-dione* (**8**), a yellow solid after purification by flash column chromatography (petroleum ether/ethyl acetate = 10/1), 43.3 mg, yield: 89%; m.p 265–266 °C. ^1^H NMR (500 MHz, CDCl_3_) δ 9.61 (s, 1H), 9.14 (s, 1H), 3.03 (s, 2H), 2.79 (s, 2H), 2.60 (s, 2H), 2.53 (s, 2H), 1.37 (s, 12H), 1.19 (s, 6H), 1.14 (s, 6H). ^13^C NMR (125 MHz, CDCl_3_) δ 198.8, 196.3, 167.3, 160.7, 159.5, 135.6, 129.9, 126.3, 125.2, 117.5, 116.9, 112.0, 84.4, 53.8, 52.7, 47.5, 42.3, 32.6, 31.9, 28.2, 28.1, 24.9. HRMS (ESI) *m*/*z*: [M + H]^+^ calcd for C_29_H_35_BNO_5_^+^ 488.2603; found 488.2604.

### 3.8. General Procedures for the Preparation of Compounds ***9***

A Schlenk tube was equipped with a magnetic stir bar and charged with 2-bromo-6,6,11,11-tetramethyl-6,7,11,12-tetrahydrochromeno[2,3,4-gh]phenanthridine-4,13(5H,10H)-dione **3h** (0.1 mmol), NaBH_4_ (1.0 equiv), CeCl_3_·7H_2_O (1.0 equiv), and MeOH (2 mL). Then, the flask was sealed under Ar and stirred at rt for 3 h. After the reaction was quenched by the addition of water, the mixture was extracted with dichloromethane, and the combined organic layer was dried over sodium sulfate. The concentration in vacuo followed by silica gel column purification with petroleum ether/ethyl acetate eluent gave the desired product **9**.

*2-bromo-6,6,11,11-tetramethyl-4,5,6,7,10,11,12,13-octahydrochromeno[2,3,4-gh] phenanthridine-4,13-diol* (**9**), a yellow solid after purification by flash column chromatography (petroleum ether/ethyl acetate = 10/1), 42.1 mg, yield: 95%; m.p 174–175 °C. ^1^H NMR (500 MHz, CDCl_3_) δ 8.03 (s, 1H), 7.55 (s, 1H), 5.39–4.99 (m, 1H), 4.84–4.82 (m, 1H), 2.78 (d, *J* = 17.2 Hz, 1H), 2.62 (d, *J* = 17.3 Hz, 1H), 2.46 (d, *J* = 17.6 Hz, 1H), 2.35 (d, *J* = 17.5 Hz, 1H), 2.11 (dd, *J* = 13.7, 6.1 Hz, 1H), 2.01 (dd, *J* = 13.9, 5.8 Hz, 1H), 1.82 (dd, *J* = 13.8, 5.7 Hz, 1H), 1.76 (dd, *J* = 13.7, 5.2 Hz, 1H), 1.19 (s, 3H), 1.15 (s, 3H), 1.06 (s, 3H), 1.01 (s, 3H). ^13^C NMR (125 MHz, CDCl_3_) δ 157.9, 154.3, 150.7, 139.4, 133.3, 128.3, 122.3, 119.1, 114.6, 110.5, 65.6, 65.1, 46.8, 45.9, 45.3, 41.4, 30.4, 30.3, 29.0, 28.6, 28.3. HRMS (ESI) *m/z*: [M + H]^+^ calcd for C_23_H_27_BrNO_3_^+^ 444.1169; found 444.1171.

## 4. Conclusions

In conclusion, we have developed an efficient double C-H activation/cyclization reaction for the construction of pyranoisoquinoline derivatives from aryl oxadiazoles and diazo compounds. This reaction features a broad substrate scope and mild conditions. In addition, large-scale preparation and further transformations of the products were carried out, revealing the practicality of the annulation method.

## Figures and Tables

**Table 1:** Optimization of the reaction conditions ^a^.

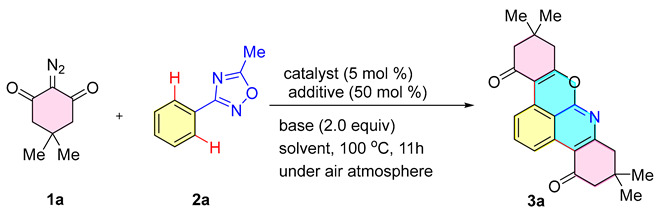					
Entry	Catalyst	Base	Additive	Solvent	Yield (%) ^b^
1	Rh_2_(OAc)_4_	Cu(OAc)_2_·H_2_O	AgNTf_2_	1,4-dioxane	nr
2	Pd(OAc)_2_	Cu(OAc)_2_·H_2_O	AgNTf_2_	1,4-dioxane	nr
3	Ru(PPh_3_)_3_Cl_2_	Cu(OAc)_2_·H_2_O	AgNTf_2_	1,4-dioxane	nr
4	[RuCl_2_(p-cymene)]_2_	Cu(OAc)_2_·H_2_O	AgNTf_2_	1,4-dioxane	nr
5	[Cp*IrCl_2_]_2_	Cu(OAc)_2_·H_2_O	AgNTf_2_	1,4-dioxane	41
6	[Cp*RhCl_2_]_2_	Cu(OAc)_2_·H_2_O	AgNTf_2_	1,4-dioxane	95
7	[Cp*RhCl_2_]_2_	Cu(OAc)_2_·H_2_O	AgSbF_6_	1,4-dioxane	25
8	[Cp*RhCl_2_]_2_	Cu(OAc)_2_·H_2_O	AgOAc	1,4-dioxane	10
9	[Cp*RhCl_2_]_2_	Cu(OAc)_2_·H_2_O	AgBF_4_	1,4-dioxane	18
10	[Cp*RhCl_2_]_2_	Cu(OAc)_2_·H_2_O	AgOTf	1,4-dioxane	15
11	[Cp*RhCl_2_]_2_	Cu(OAc)_2_·H_2_O	none	1,4-dioxane	nr
12	[Cp*RhCl_2_]_2_	Cu(OAc)_2_·H_2_O	AgNTf_2_	toluene	nr
13	[Cp*RhCl_2_]_2_	Cu(OAc)_2_·H_2_O	AgNTf_2_	MeCN	nr
14	[Cp*RhCl_2_]_2_	Cu(OAc)_2_·H_2_O	AgNTf_2_	DCE	nr
15 ^c^	[Cp*RhCl_2_]_2_	Cu(OAc)_2_·H_2_O	AgNTf_2_	1,4-dioxane	85
16 ^d^	[Cp*RhCl_2_]_2_	Cu(OAc)_2_·H_2_O	AgNTf_2_	1,4-dioxane	92
17 ^e^	[Cp*RhCl_2_]_2_	Cu(OAc)_2_·H_2_O	AgNTf_2_	1,4-dioxane	73
18 ^f^	[Cp*RhCl_2_]_2_	Cu(OAc)_2_·H_2_O	AgNTf_2_	1,4-dioxane	80
19 ^g^	[Cp*RhCl_2_]_2_	Cu(OAc)_2_·H_2_O	AgNTf_2_	1,4-dioxane	77
20 ^h^	[Cp*RhCl_2_]_2_	Cu(OAc)_2_·H_2_O	AgNTf_2_	1,4-dioxane	75
21 ^i^	[Cp*RhCl_2_]_2_	Cu(OAc)_2_·H_2_O	AgNTf_2_	1,4-dioxane	79
22 ^j^	[Cp*RhCl_2_]_2_	Cu(OAc)_2_·H_2_O	AgNTf_2_	1,4-dioxane	82
23	[Cp*RhCl_2_]_2_	AgOAc	AgNTf_2_	1,4-dioxane	39
24	[Cp*RhCl_2_]_2_	NaOAc	AgNTf_2_	1,4-dioxane	nr
25	[Cp*RhCl_2_]_2_	KOAc	AgNTf_2_	1,4-dioxane	nr
26 ^k^	[Cp*RhCl_2_]_2_	Cu(OAc)_2_·H_2_O	AgNTf_2_	1,4-dioxane	46
27 ^l^	[Cp*RhCl_2_]_2_	Cu(OAc)_2_·H_2_O	AgNTf_2_	1,4-dioxane	71
28 ^m^	[Cp*RhCl_2_]_2_	Cu(OAc)_2_·H_2_O	AgNTf_2_	1,4-dioxane	80
29 ^n^	[Cp*RhCl_2_]_2_	Cu(OAc)_2_·H_2_O	AgNTf_2_	1,4-dioxane	93
30	[Cp*RhCl_2_]_2_	Cu(OAc)_2_·H_2_O	AgNTf_2_ (10 mol%)	1,4-dioxane	53
31	[Cp*RhCl_2_]_2_	Cu(OAc)_2_·H_2_O	AgNTf_2_ (20 mol%)	1,4-dioxane	67
32	[Cp*RhCl_2_]_2_	Cu(OAc)_2_·H_2_O	AgNTf_2_ (30 mol%)	1,4-dioxane	75
33	[Cp*RhCl_2_]_2_	Cu(OAc)_2_·H_2_O	AgNTf_2_(40 mol%)	1,4-dioxane	82
34	[Cp*RhCl_2_]_2_	Cu(OAc)_2_·H_2_O	AgNTf_2_ (60 mol%)	1,4-dioxane	85

^a^ Reaction conditions: 1a (0.35 mmol), 2a (0.1 mmol), catalyst (5 mol %), additive (50 mol %), and Base (2.0 equiv) in solvent (3 mL) at 100 °C under air atmosphere for 11 h. ^b^ Isolated yields. nr: no reaction. ^c^ The catalyst loading was 4 mol %. ^d^ The catalyst loading was 6 mol %. ^e^ The additive loading was 40 mol %. ^f^ The additive loading was 60 mol %. ^g^ At 90 °C. ^h^ At 110 °C. ^i^ For 10 h. ^j^ For 12 h. ^k^ Cu(OAc)_2_·H_2_O (0.5 equiv). ^l^ Cu(OAc)_2_·H_2_O (1.0 equiv). ^m^ Cu(OAc)_2_·H_2_O (1.5 equiv). ^n^ Cu(OAc)_2_·H_2_O (2.5 equiv).

## Data Availability

The data are contained within the article and Supplementary Materials.

## Supplementary Materials

The following supporting information can be downloaded at: https://www.mdpi.com/article/10.3390/molecules30010149/s1. The characterization data for products **3**, **4**, **5**, **6**, **7**, **8,** and **9,** including the ^1^H-NMR, ^13^C-NMR, and ^19^F-NMR spectroscopies, are available online. CCDC 2132351 contains the supplementary crystallographic data for this paper. These data can be obtained free of charge via www.ccdc.cam.ac.uk/data_request/cif [14] or by emailing data_request@ccdc.cam.ac.uk or by contacting the Cambridge Crystallographic Data Centre, 12 Union Road, Cambridge, CB2 1EZ, UK; fax:+44-1223-336033.
