# Supplementary material for: Rh(III)-Catalyzed Double Annulation of 3-Phenyl-1,2,4-oxadiazoles with 2-Diazo-1,3-diketones: Access to Pyran-Fused Isoquinolines
Source: Molecules. 2025 Jan 2;30(1):149. doi: 10.3390/molecules30010149 (PMC11721317; doi:10.3390/molecules30010149)

Supporting Information

Rh(III)-Catalyzed Double Annulation of 3-Phenyl-1,2,4-Oxadiazoles with 2-Diazo-1,3-diketones: Access to Pyran-Fused Isoquinolines

Enshen Zhang, Mei Sun and Lvlv Gao *

School of Chemistry and Materials Engineering, Huainan Normal University, Huainan 232038, China; zhes@hnnu.edu.cn (E.Z.); smbs@hnnu.edu.cn (M.S.)

* Correspondence: gaolvlv@hnnu.edu.cn

Table of Contents

S1. General methods 2

S2. General procedure for synthesis of oxadiazoles 4

S3. Characterization data and NMR spectra 5

S4. X-ray crystal data of compound 3a 33

S5. HRMS spectra 35

S1. General methods

All reagents (catalysts, additives, chemical reagents) were purchased from commercial suppliers and used directly without purification (Table S1、Table S2、Table S3). Silica gel (200-300 mesh) was used for column chromatography. Melting points were recorded on an uncorrected Melting Point instrument. ^{1}H NMR spectra were recorded on a 500 MHz or 400 MHz spectrometer. Spectra were referenced internally to the residual proton resonance in $CDCl_3$ (δ 7.26 ppm), or with TMS (δ 0.00 ppm) as the internal standard. Chemical shifts (δ) were reported as part per million (ppm) in δ scale downfield from TMS. ^{13}C NMR spectra were recorded on a 125 MHz or 100 MHz spectrometer and the spectra were referenced to $CDCl_3$ (δ = 77.00 ppm, the middle peak). Coupling constants (J) were reported in Hertz (Hz).

Table S1. The purities and sources of catalysts.

Catalyst	Purity	Source
$Rh_2(OAc)_4$	98%	Shanghai Haohong Scientific Co., Ltd.
$Pd(OAc)_2$	98%	Shanghai Haohong Scientific Co., Ltd.
$Ru(PPh_3)_3Cl_2$	98%	Shanghai Haohong Scientific Co., Ltd.
$[RuCl_2(p\text{-cymene})]_2$	98%	Shanghai Haohong Scientific Co., Ltd.
$[Cp^*IrCl_2]_2$	98%	Shanghai Haohong Scientific Co., Ltd.
$[Cp^*RhCl_2]_2$	98%	Shanghai Haohong Scientific Co., Ltd.
$PdCl_2$	98.0%	TCI Shanghai
$Pd(PPh_3)_4$	98%	Shanghai Haohong Scientific Co., Ltd.
$Pd_2(dba)_3$	98%	Saen Chemical Technology (Shanghai) Co., Ltd.

Table S2. The purities and sources of additives.

Additive	Purity	Source
$Cu(OAc)_2 \cdot H_2O$	98%	Shanghai Haohong Scientific Co., Ltd.
$AgNTf_2$	98%	Shanghai Haohong Scientific Co., Ltd.
$AgSbF_6$	98%	Shanghai Haohong Scientific Co., Ltd.
AgOAc	98%	Shanghai Haohong Scientific Co., Ltd.
$AgBF_4$	98%	TCI Shanghai
AgOTf	98%	TCI Shanghai
K_3PO_4	98%	Shanghai Haohong Scientific Co., Ltd.
PPh_3	95	TCI Shanghai
CuI	99%	Shanghai Haohong Scientific Co., Ltd.
K_2CO_3	99%	Shanghai Haohong Scientific Co., Ltd.
NaOAc	>98.5%	TCI Shanghai
KOAc	99%	Shanghai Haohong Scientific Co., Ltd.
RuPhos	98%	Shanghai Haohong Scientific Co., Ltd.
NaO^tBu	98%	Shanghai Haohong Scientific Co., Ltd.
XPhos	98%	Shanghai Haohong Scientific Co., Ltd.
$CeCl_3 \cdot 7H_2O$	99%	Shanghai Haohong Scientific Co., Ltd.
$NaBH_4$	98%	Saen Chemical Technology (Shanghai) Co., Ltd.

Table S3. The purities and sources of chemical reagents.

Chemical Reagent	Purity	Source
1,4-Dioxane	AR, ≥99.5%	SINOPHARM
Toluene	AR, ≥99.5%	SINOPHARM
MeCN	AR, ≥99.5%	SINOPHARM
DCE	AR, ≥99.5%	SINOPHARM
DMAc	AR, ≥99.0%	SINOPHARM
Et_3N	AR, ≥99.0%	SINOPHARM
EtOH	AR, ≥99.7%	SINOPHARM
MeOH	AR, ≥99.7%	SINOPHARM
Petroleum Ether	AR	SINOPHARM
Ethyl Acetate	AR, ≥99.5%	SINOPHARM

S2. General procedure for synthesis of oxadiazoles

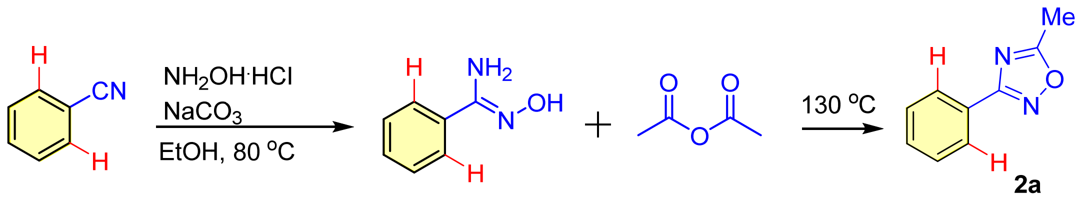

2a

A solution with benzonitrile (500 mg, 5.0 mmol), sodium carbonate (2.0 equiv), hydroxylamine hydrochloride (2.0 equiv), and EtOH (7.5 mL) was heated to reflux overnight. After cooling to room temperature, the mixture was filtered over a pad of Celite and evaporated to dryness to give crude benzamidoxime. Then the acetic anhydride (1.1 equiv) was added and the mixture was heated at 130 °C for 1 h. After cooling to room temperature, the mixture solution was extracted with EtOAc and the organic layer was dried over Na_2SO_4 and the solvent was evaporated. The residue was purified by column chromatography (petroleum ether/ethyl acetate = 50/1) to give **2a** as white solid (528 mg, 66%).

S3. Characterization data and NMR spectra

6,6,11,11-tetramethyl-6,7,11,12-tetrahydrochromeno[2,3,4-gh]phenanthridine-4,13(5H,10H)-dione **(3a)**

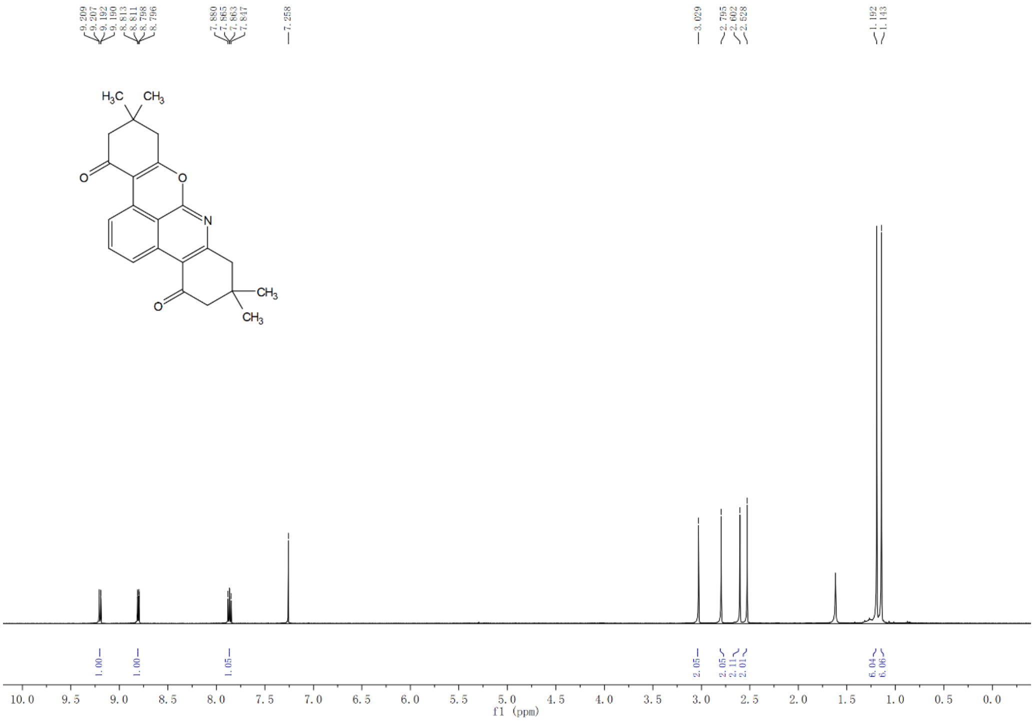

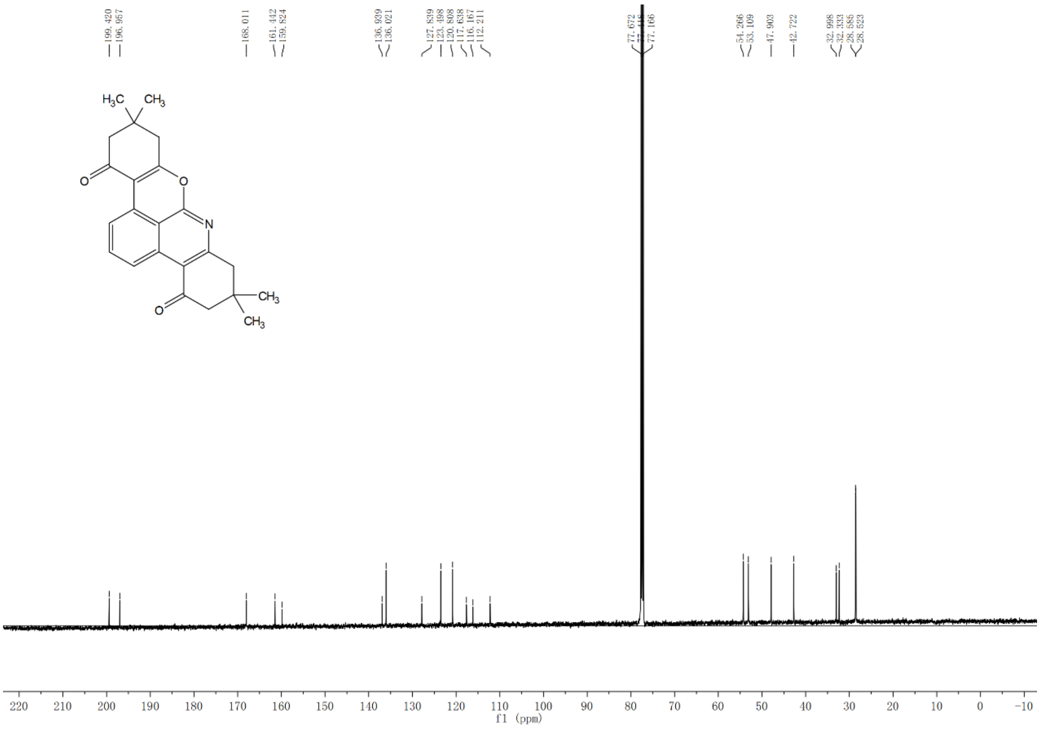

2,6,6,11,11-pentamethyl-6,7,11,12-tetrahydrochromeno[2,3,4-gh]phenanthridine-4,13(5H,10H)-dione **(3b)**

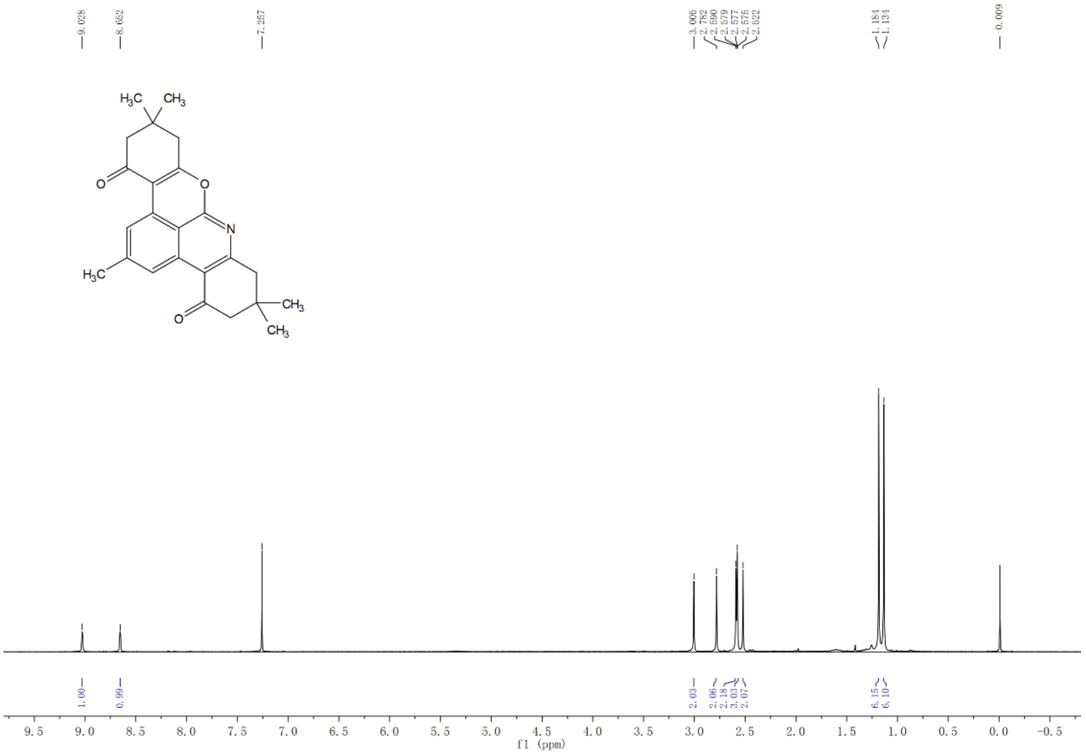

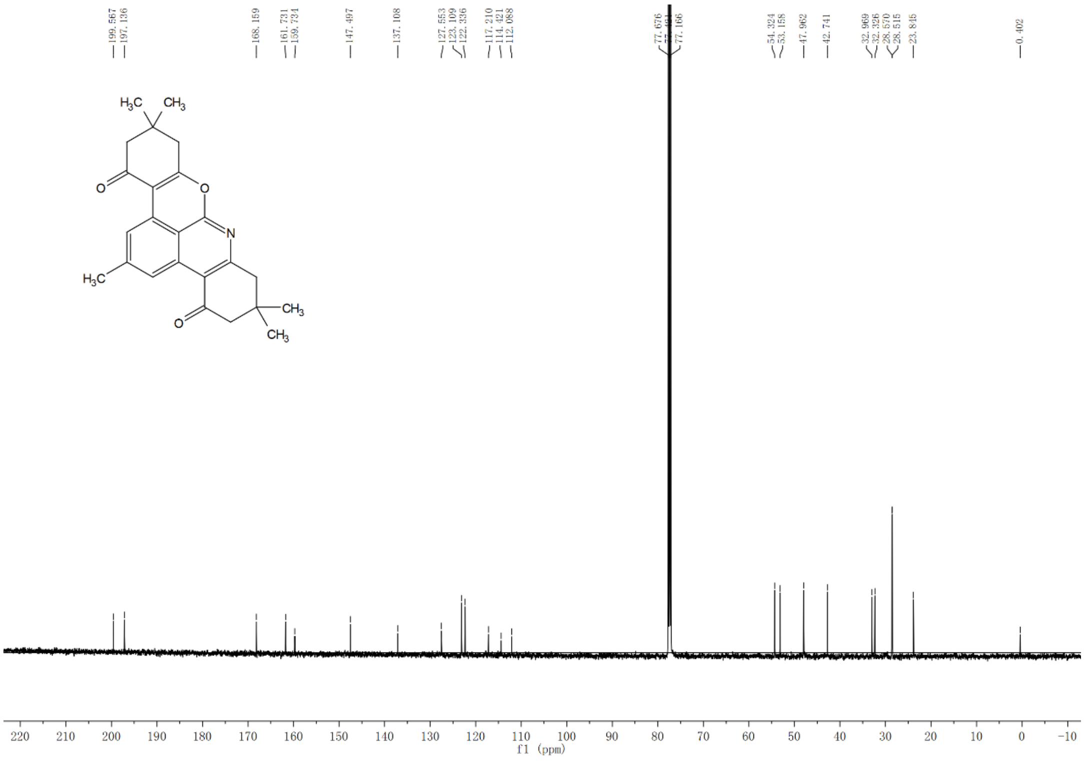

2-butyl-6,6,11,11-tetramethyl-6,7,11,12-tetrahydrochromeno[2,3,4-gh]phenanthridine-4,13(5H,10H)-dione **(3c)**

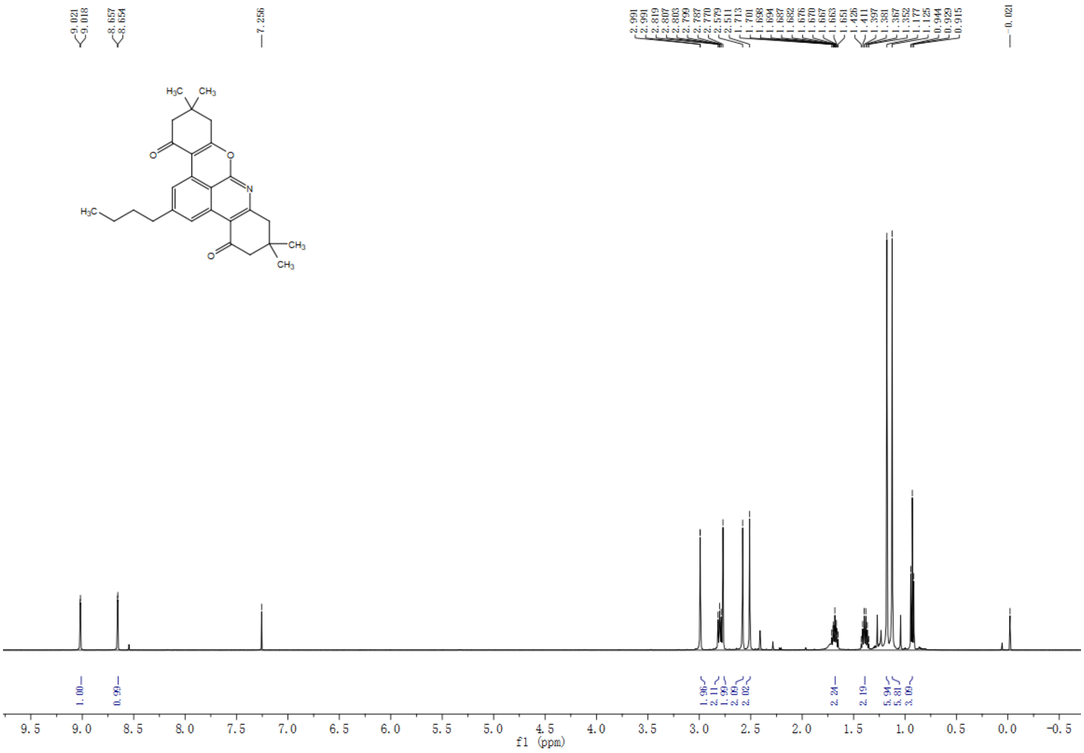

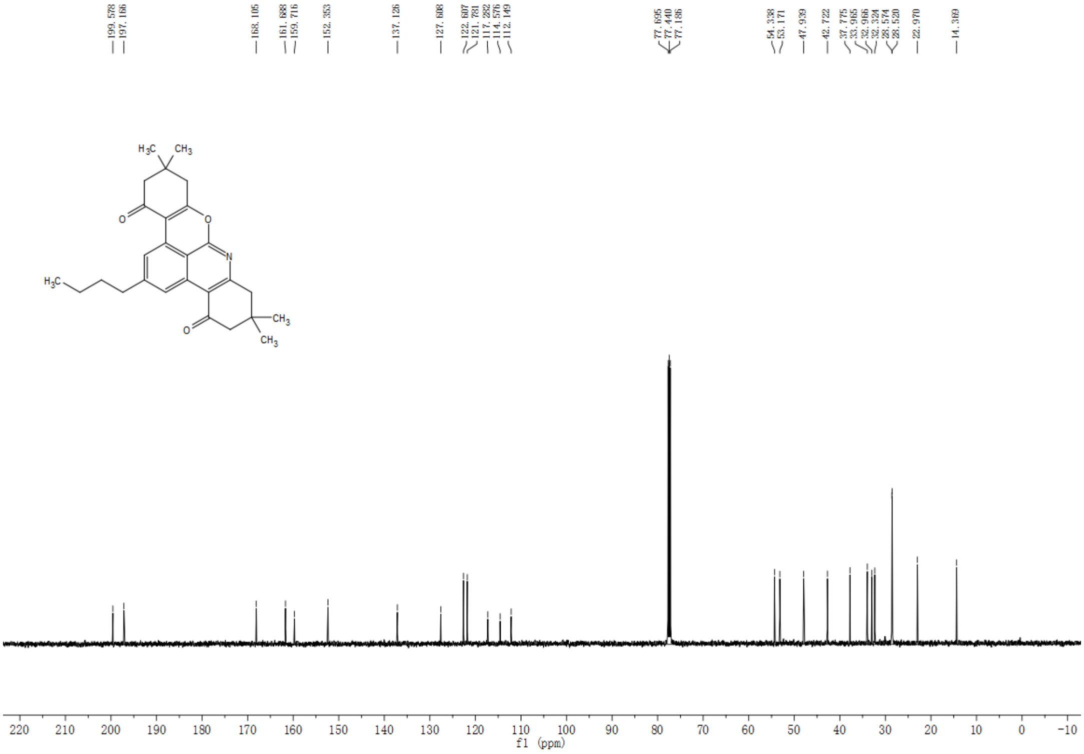

2-methoxy-6,6,11,11-tetramethyl-6,7,11,12-tetrahydrochromeno[2,3,4-gh]phenanthridine-4,13(5H,10H)-dione **(3d)**

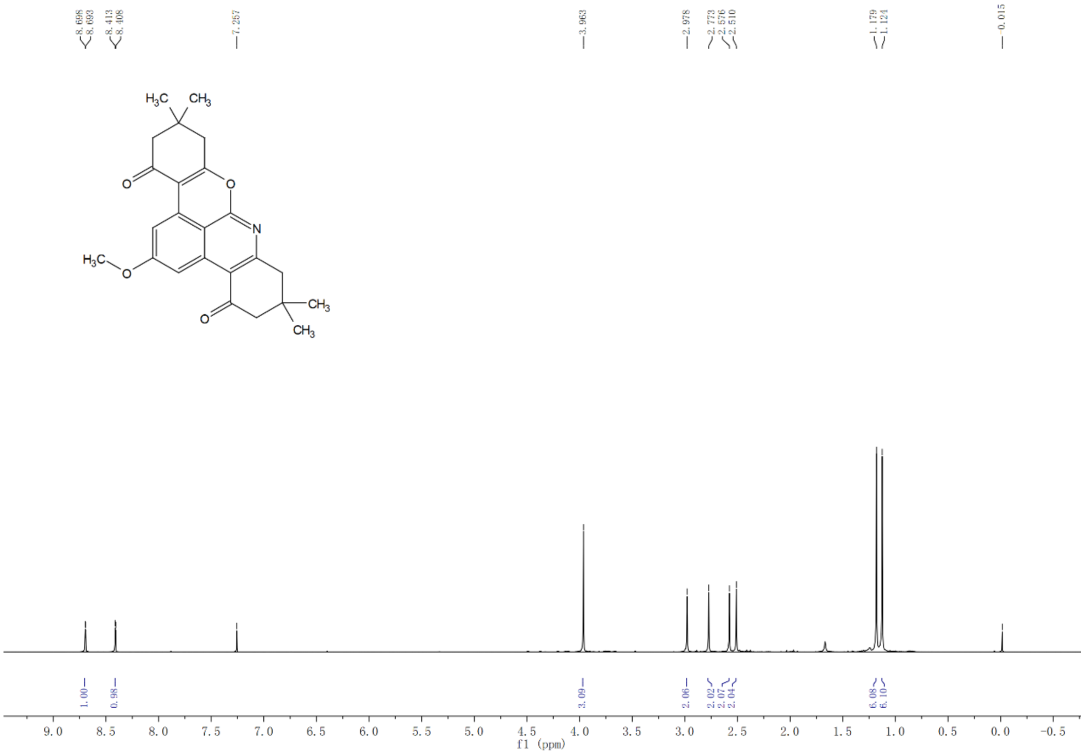

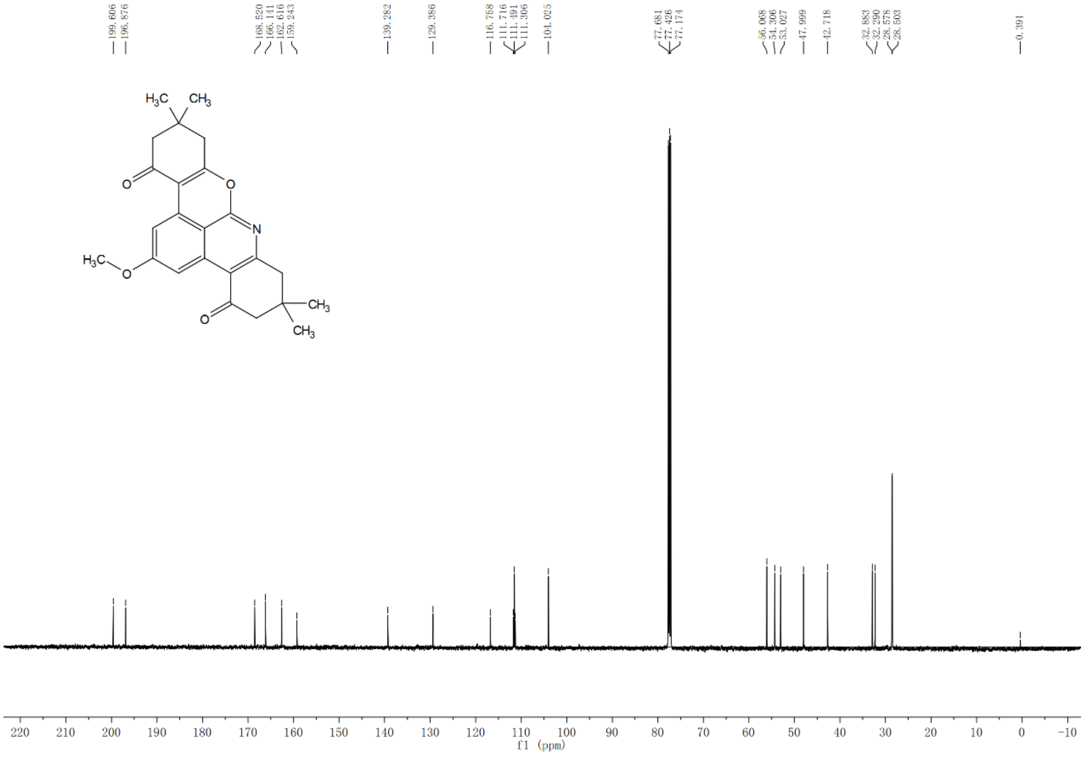

6,6,11,11-tetramethyl-2-(methylthio)-6,7,11,12-tetrahydrochromeno[2,3,4-gh]phenanthridine-4,13(5H,10H)-dione **(3e)**

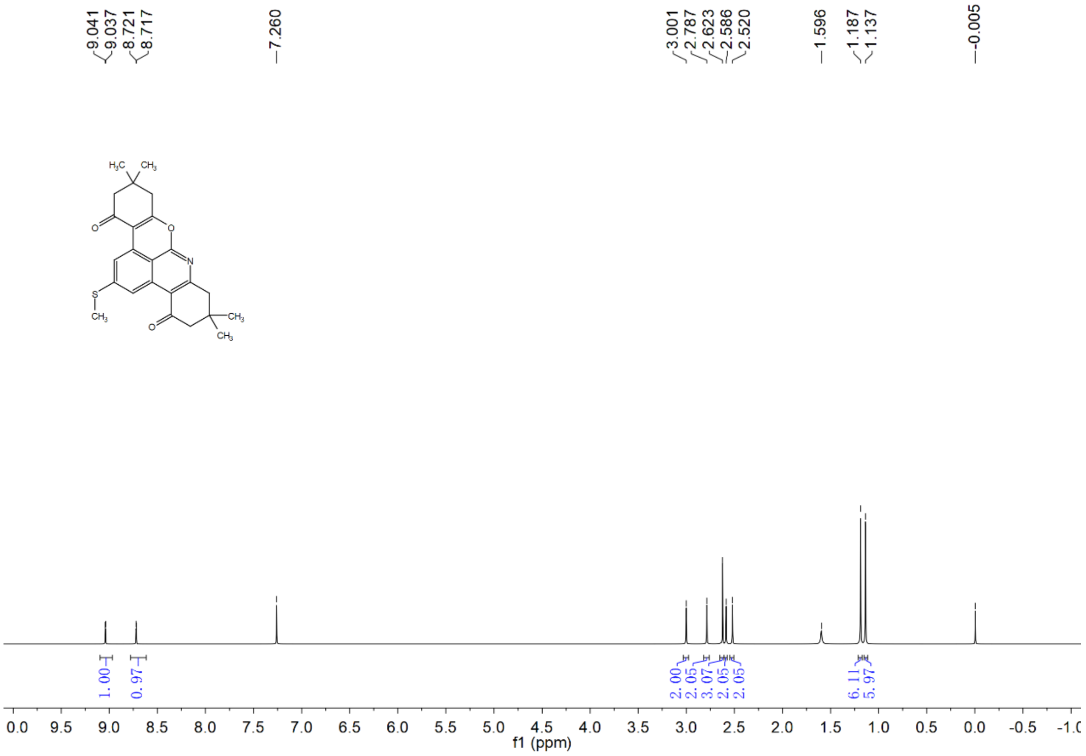

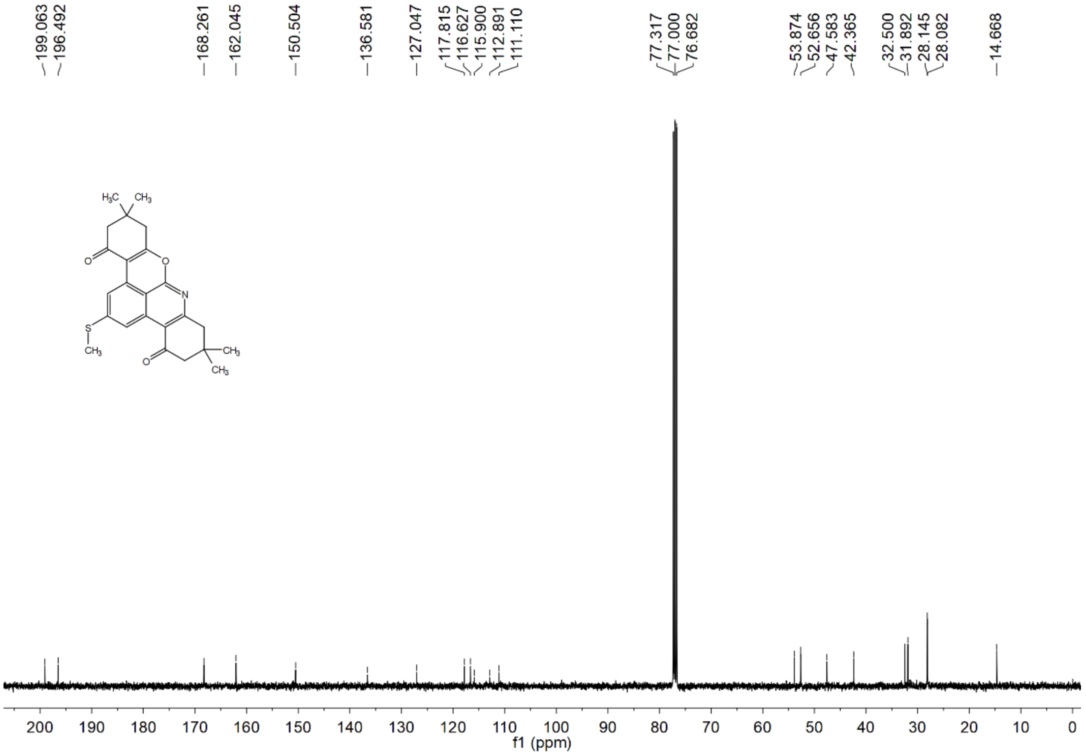

2-fluoro-6,6,11,11-tetramethyl-6,7,11,12-tetrahydrochromeno[2,3,4-gh]phenanthridine-4,13(5H,10H)-dione **(3f)**

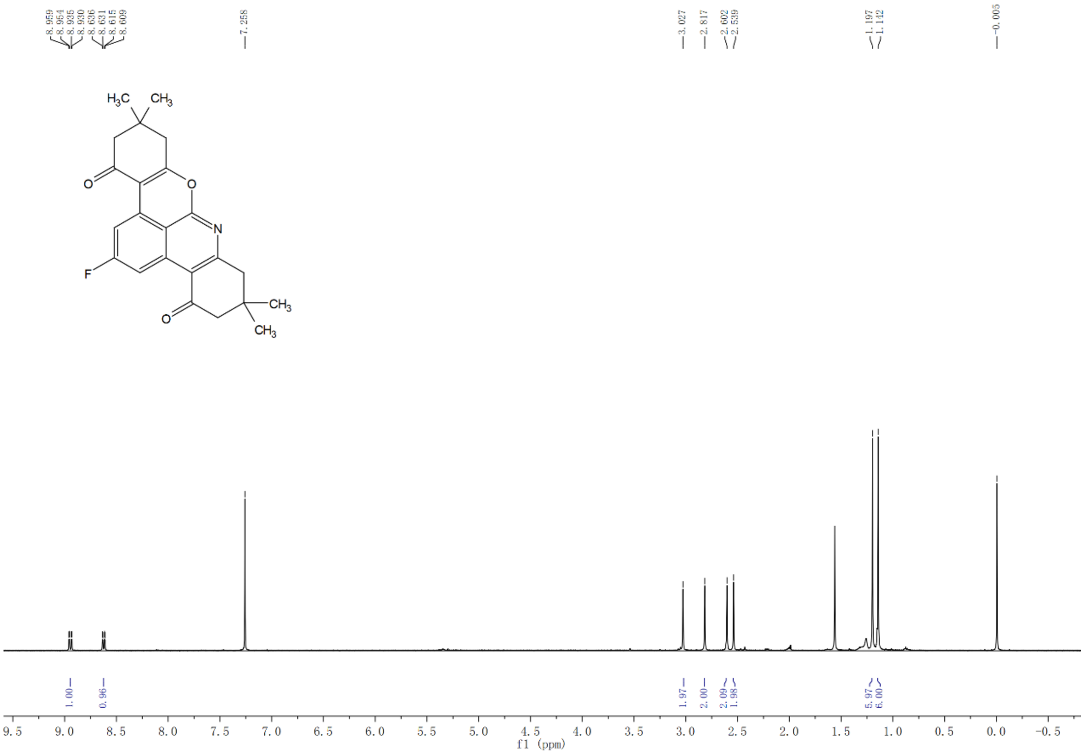

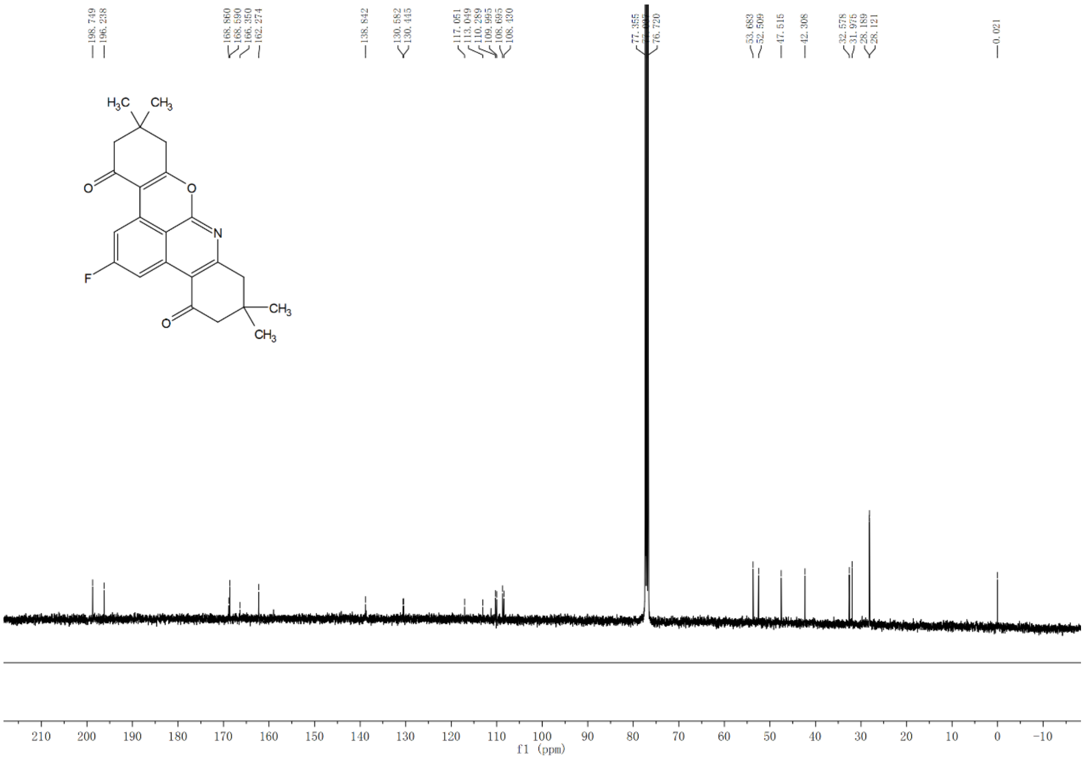

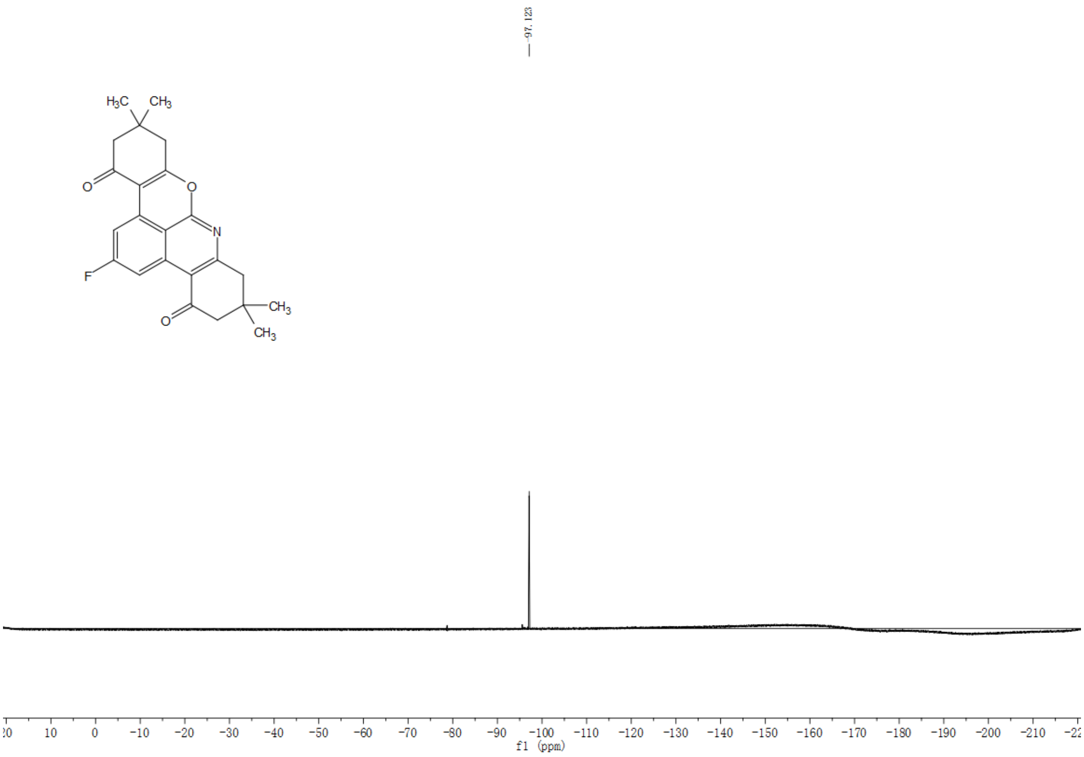

2-chloro-6,6,11,11-tetramethyl-6,7,11,12-tetrahydrochromeno[2,3,4-gh]phenanthridine-4,13(5H,10H)-dione **(3g)**

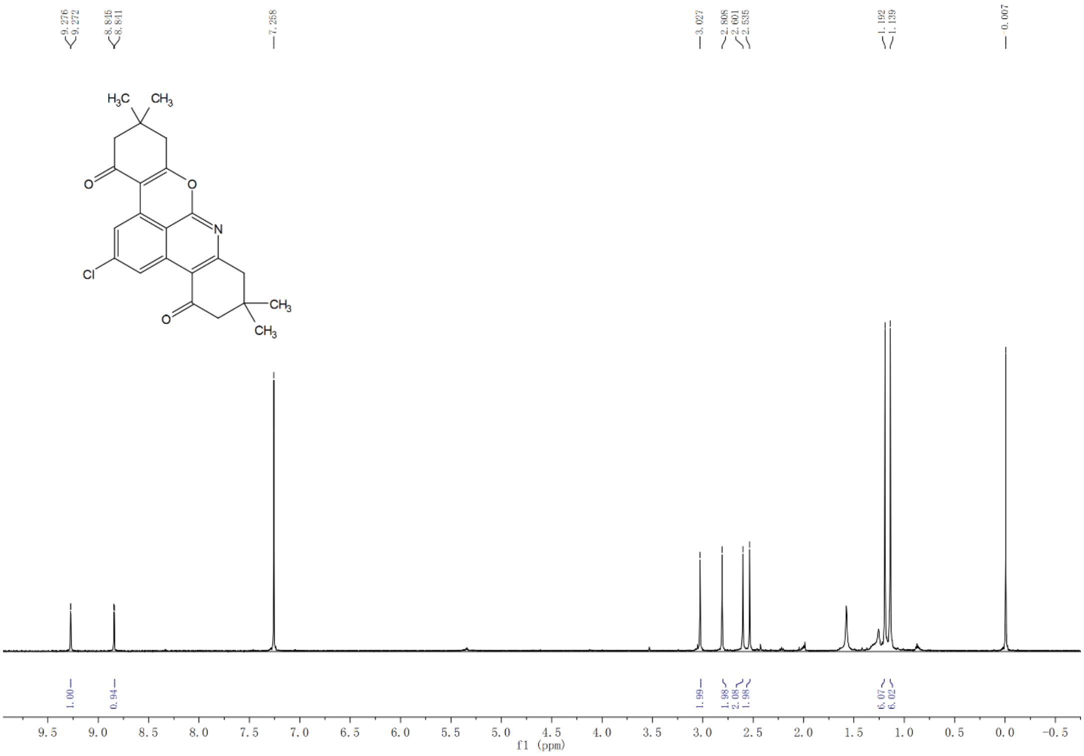

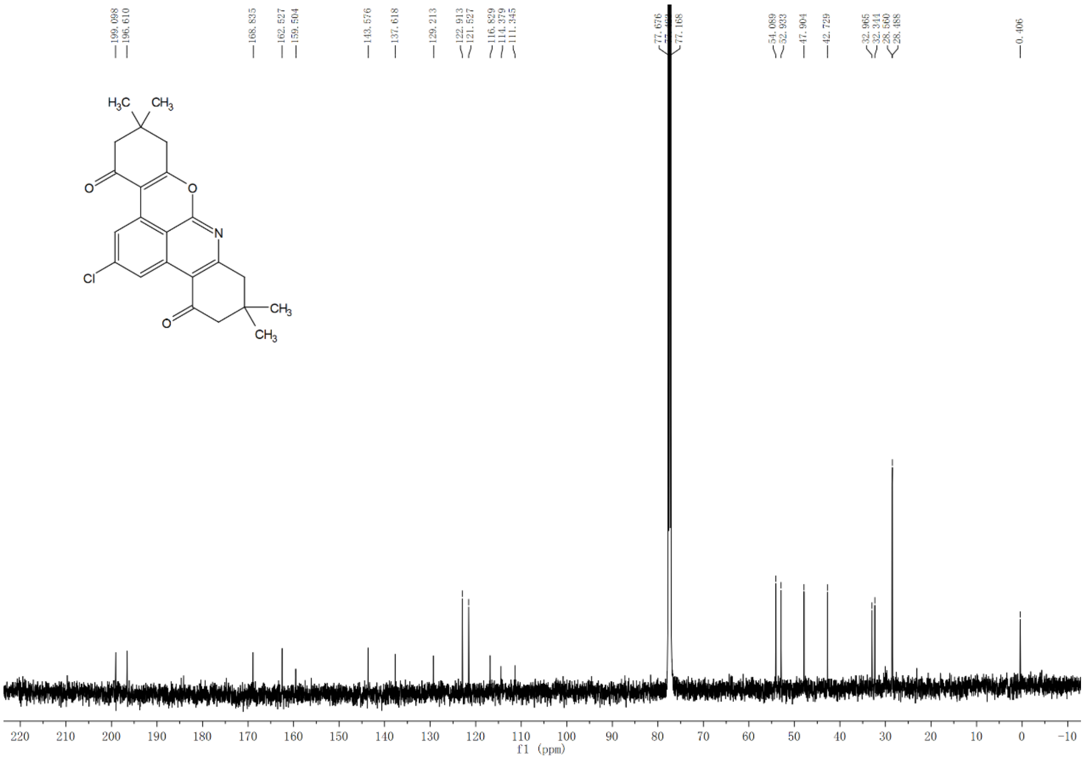

2-bromo-6,6,11,11-tetramethyl-6,7,11,12-tetrahydrochromeno[2,3,4-gh]phenanthridine-4,13(5H,10H)-dione **(3h)**

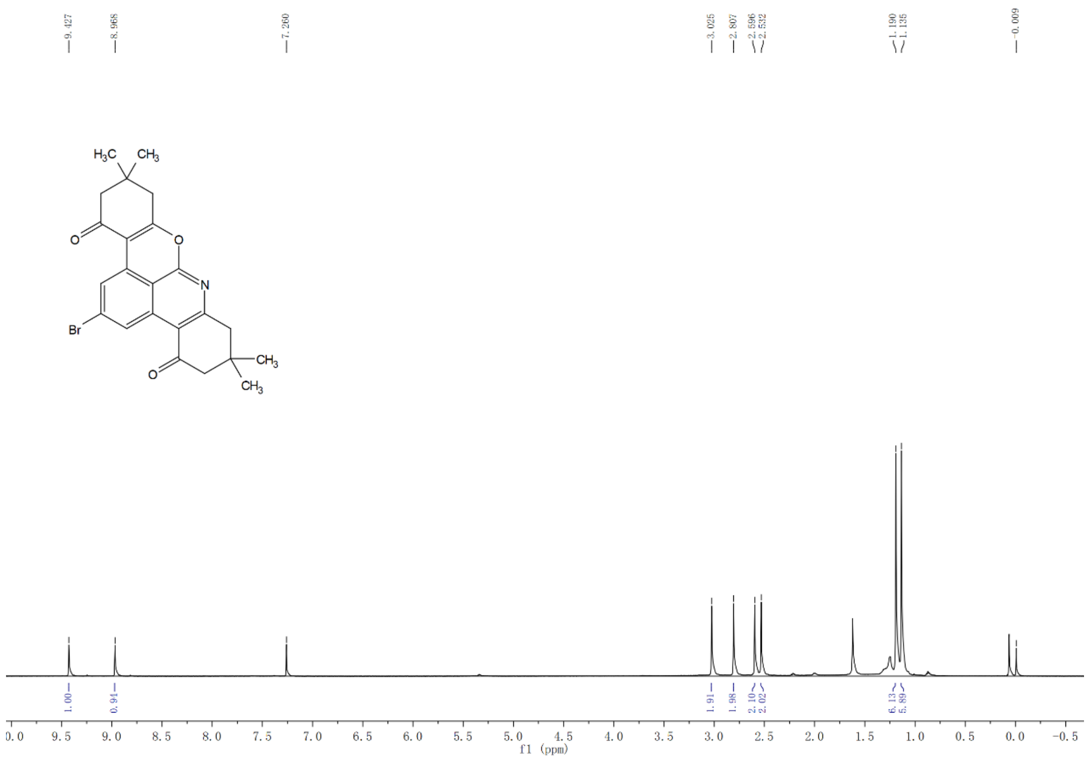

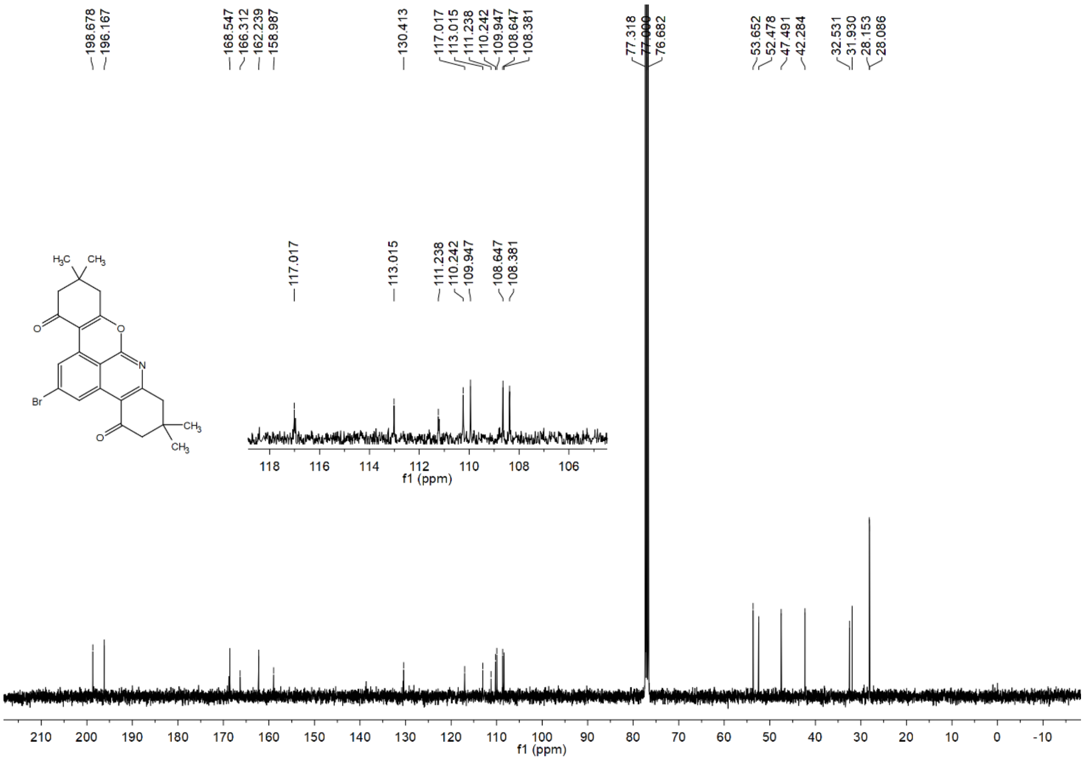

6,6,11,11-tetramethyl-2-(trifluoromethyl)-6,7,11,12-tetrahydrochromeno[2,3,4-gh]phenanthridine-4,13(5H,10H)-dione **(3i)**

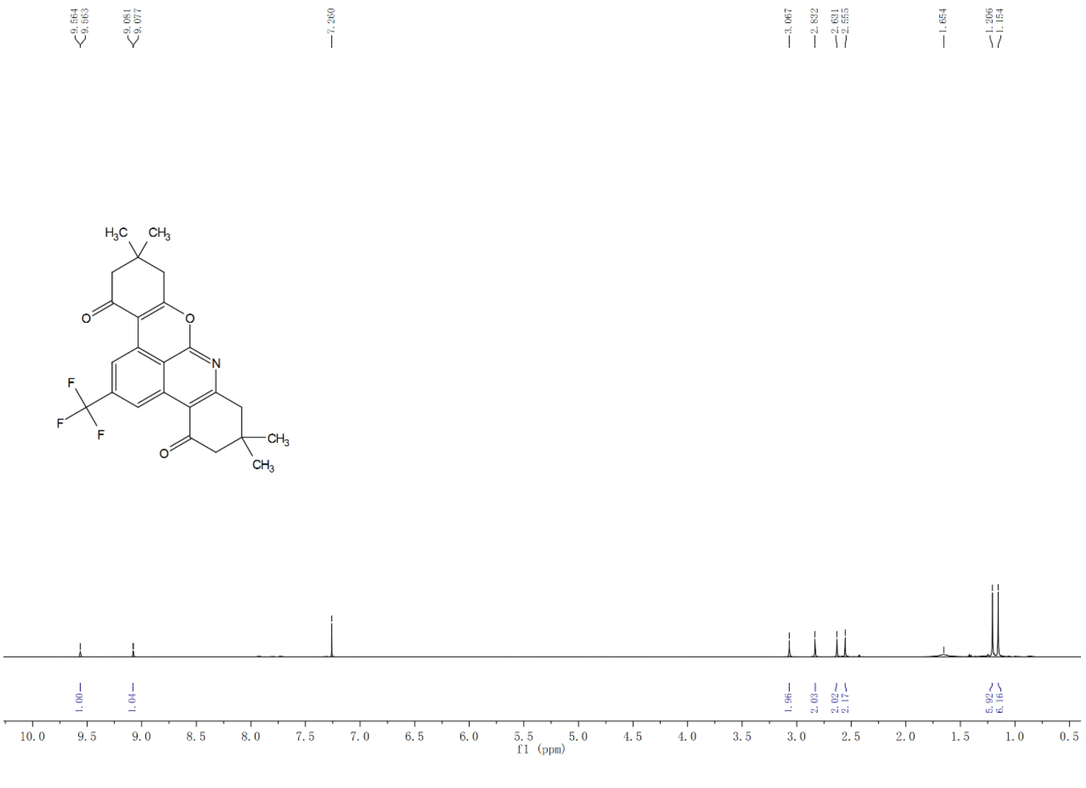

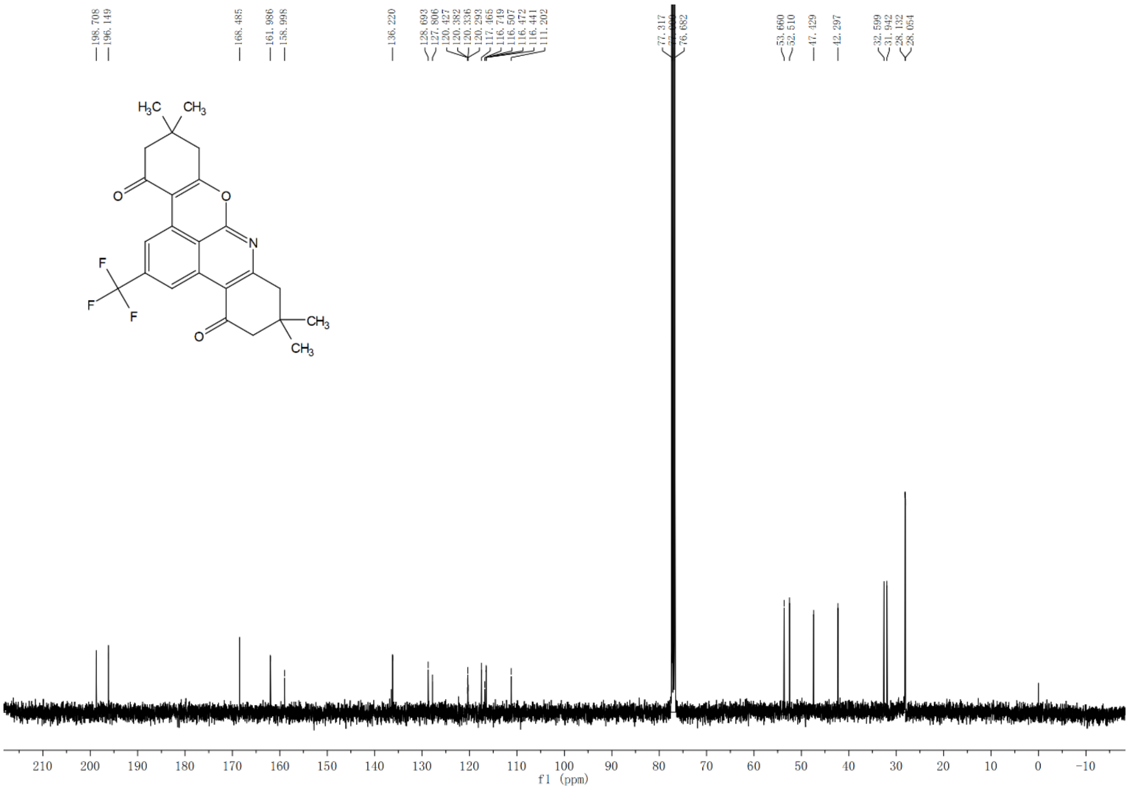
f1 (ppm)

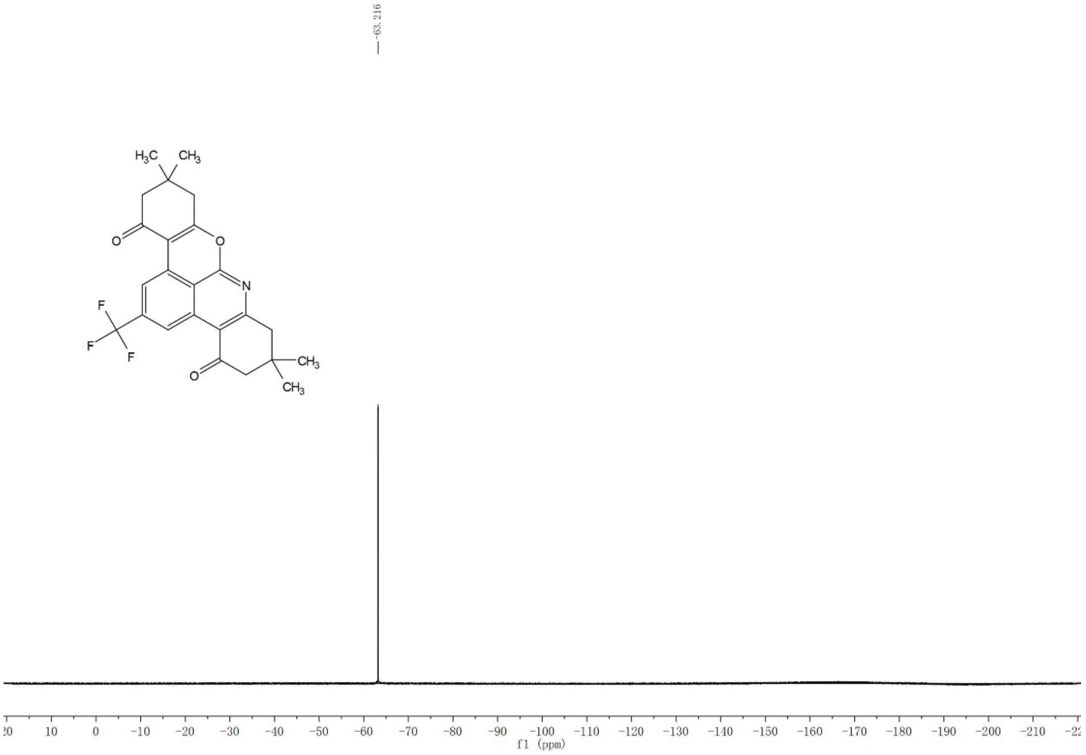
—63.216
f1 (ppm)

6,6,11,11-tetramethyl-2-phenyl-6,7,11,12-tetrahydrochromeno[2,3,4-gh]phenanthridine-4,13(5H,10H)-dione **(3j)**

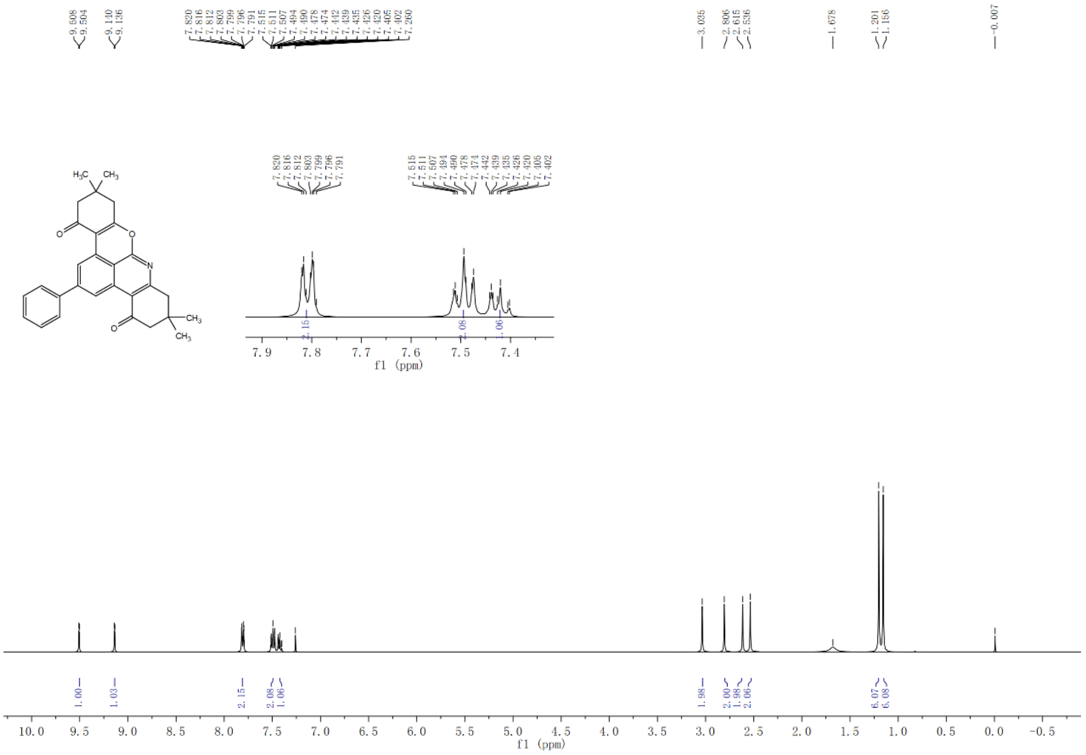

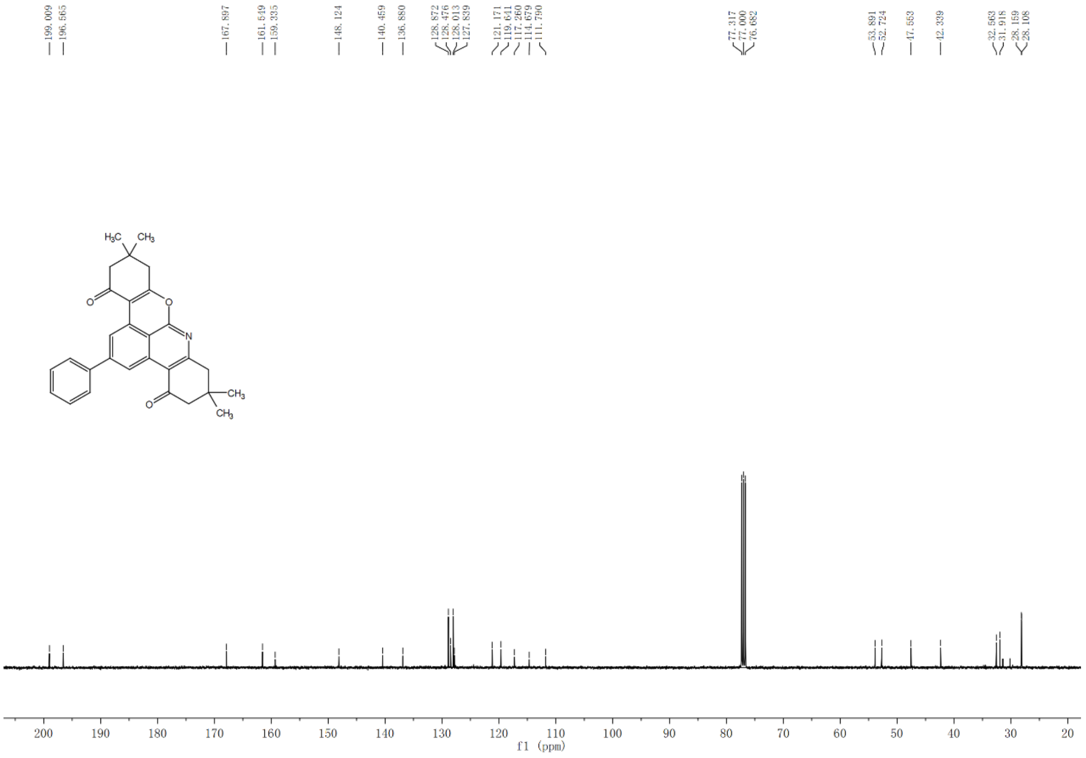

3-methoxy-6,6,11,11-tetramethyl-6,7,11,12-tetrahydrochromeno[2,3,4-gh]phenanthridine-4,13(5H,10H)-dione **(3k)**

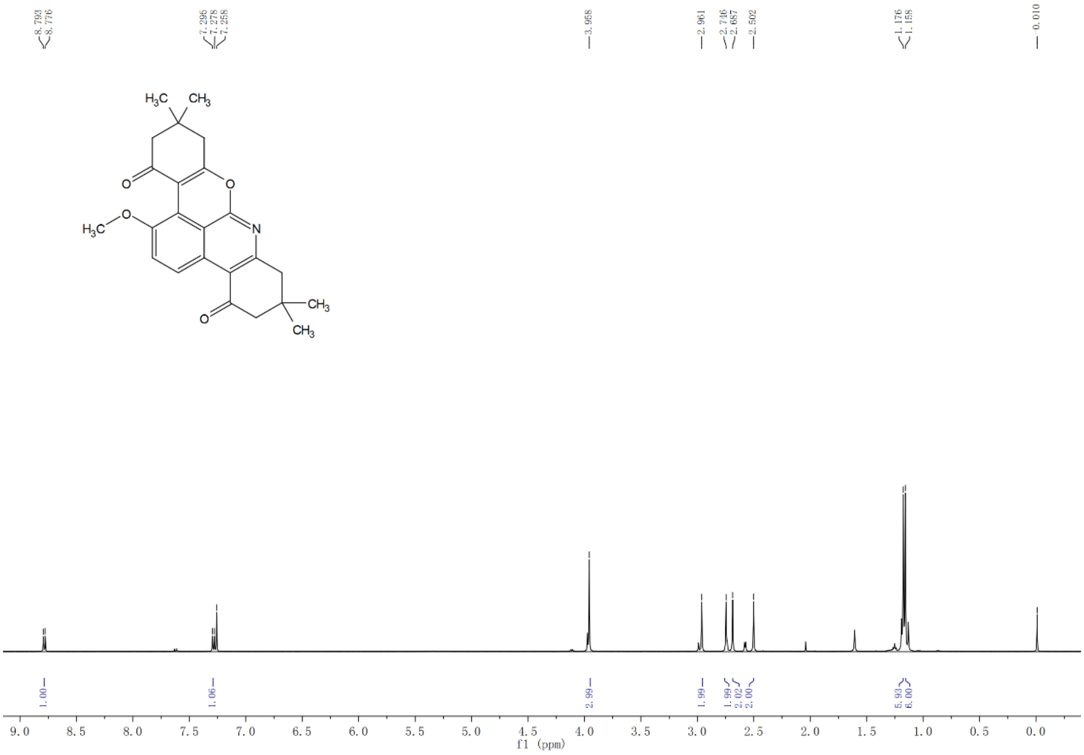

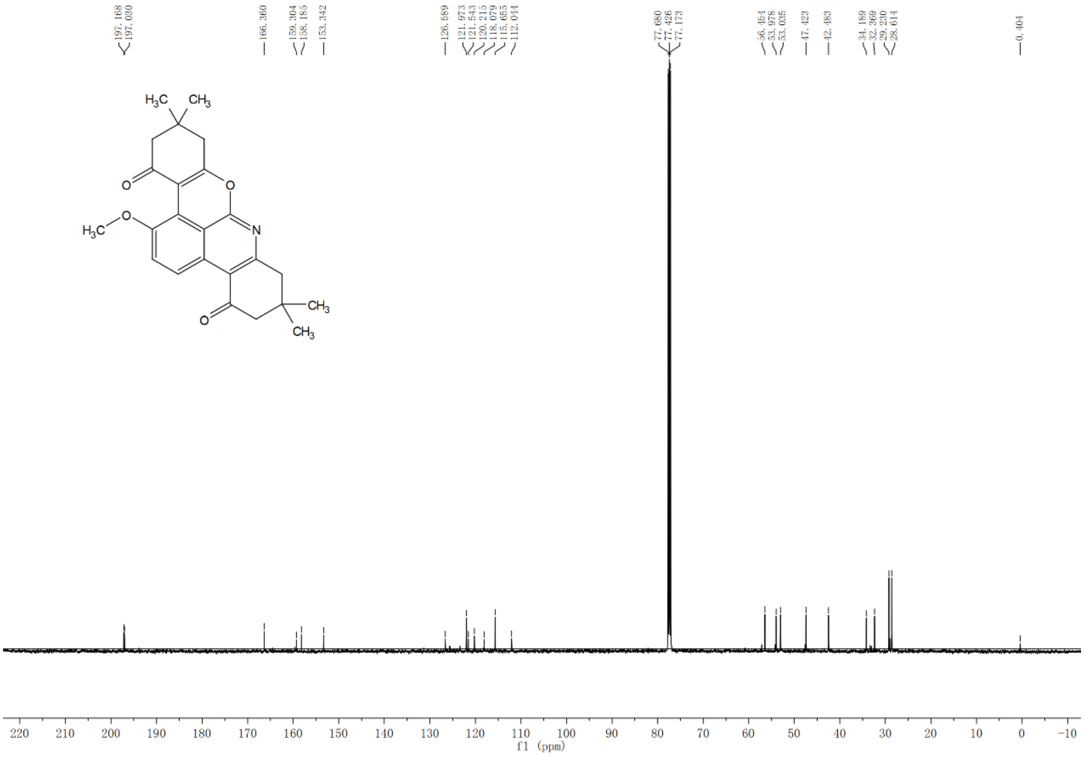

3-fluoro-6,6,11,11-tetramethyl-6,7,11,12-tetrahydrochromeno[2,3,4-gh]phenanthridine-4,13(5H,10H)-dione **(3l)**

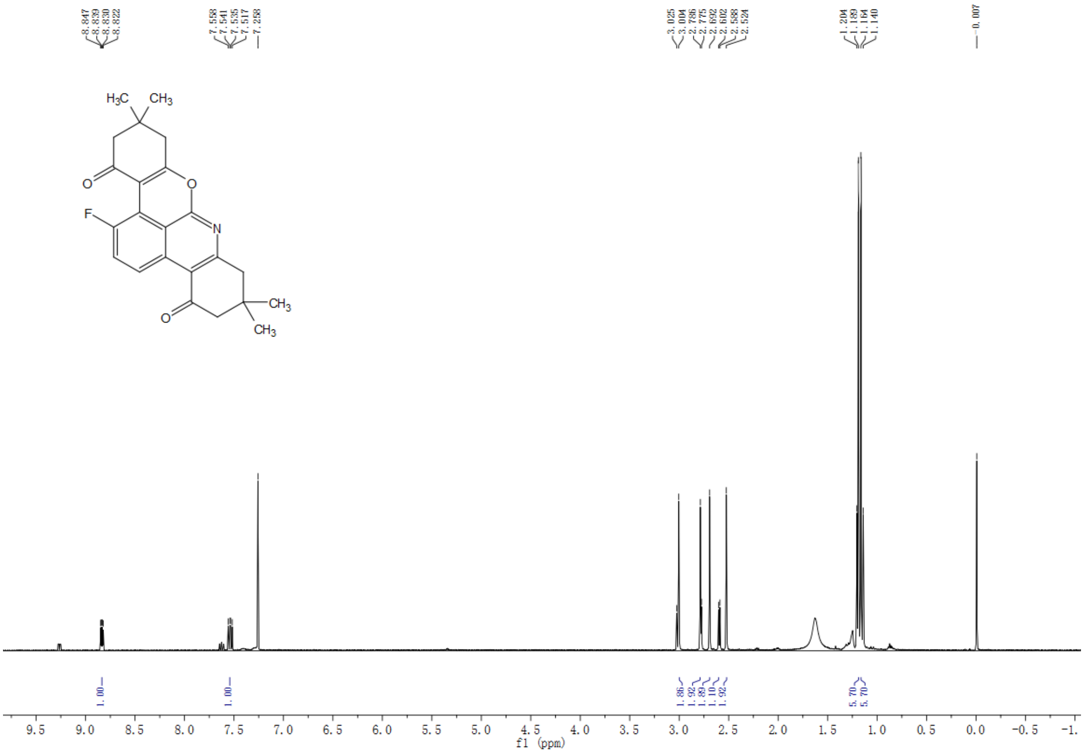

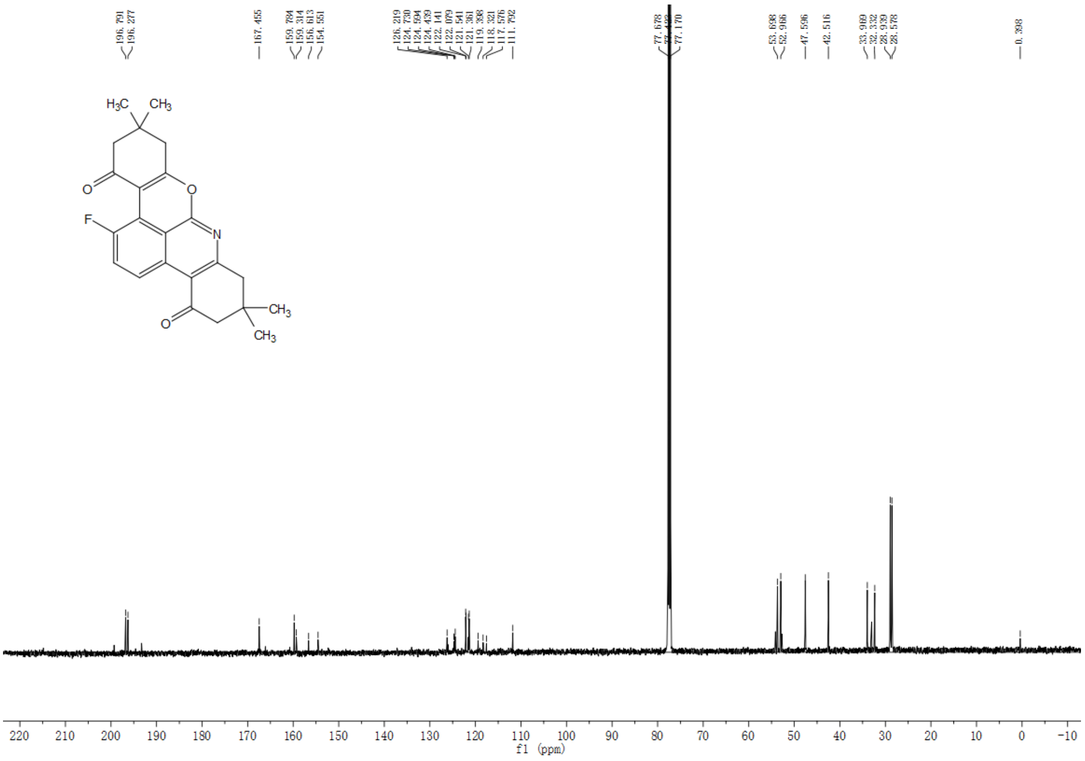

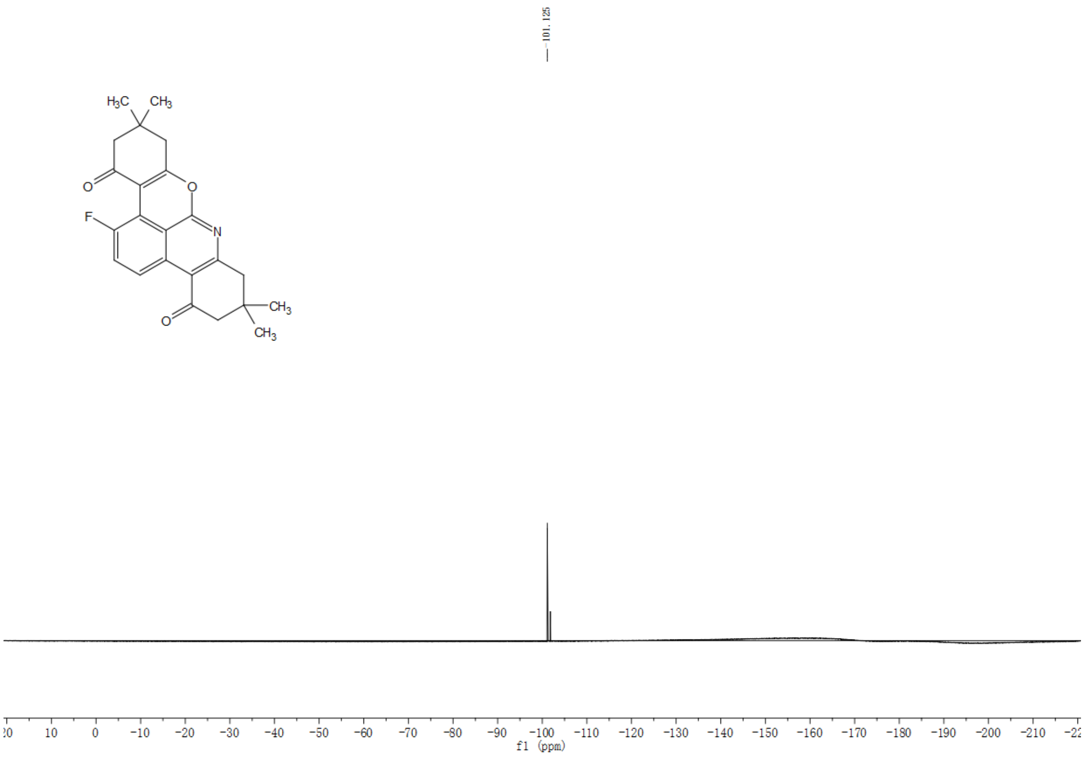

6,11-dimethyl-6,7,11,12-tetrahydrochromeno[2,3,4-gh]phenanthridine-4,13(5H,10H)-dione **(3m)**

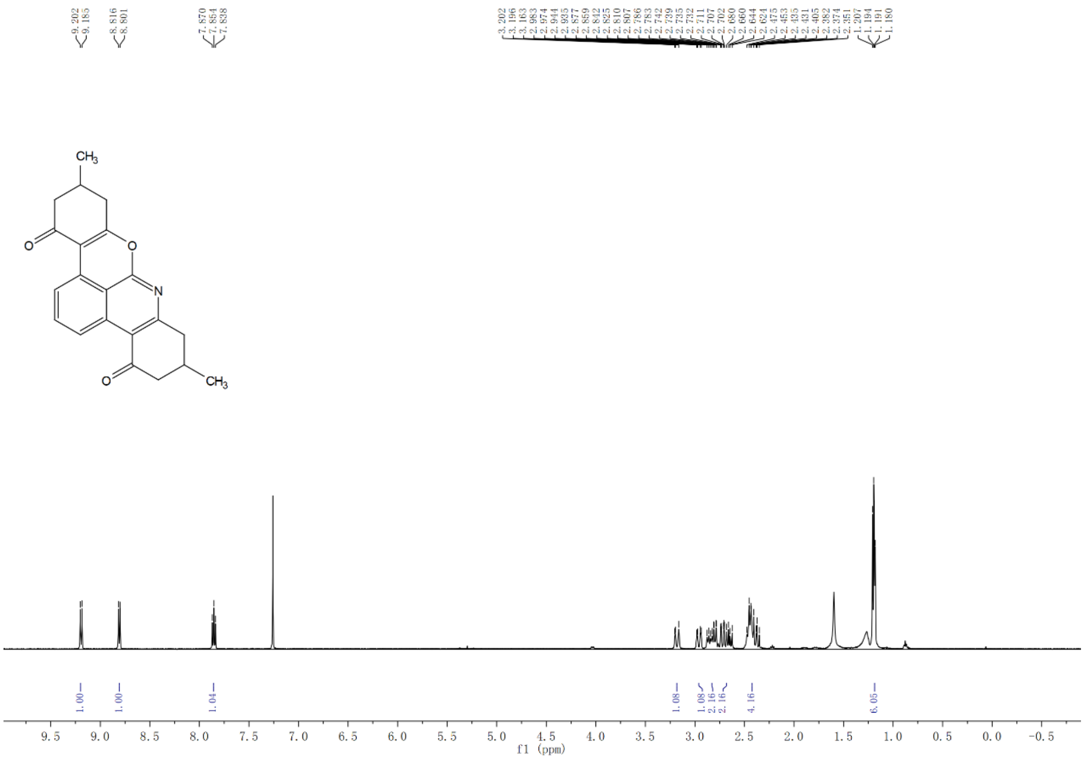

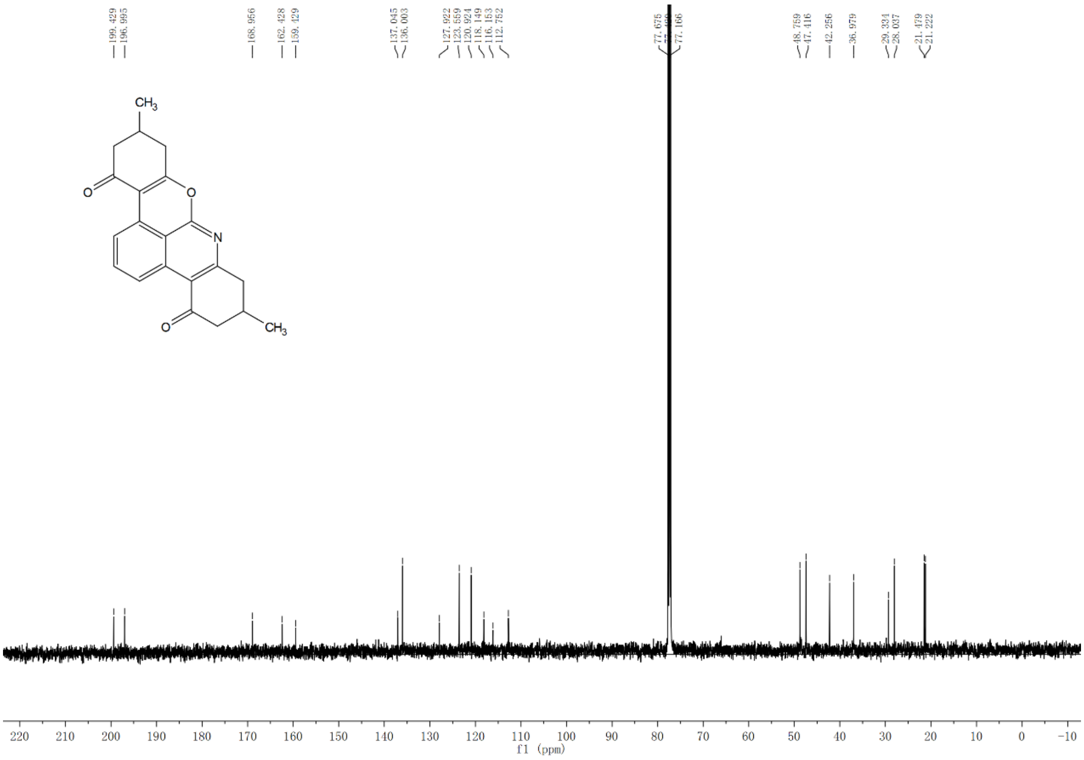

6,7,11,12-tetrahydrochromeno[2,3,4-gh]phenanthridine-4,13(5H,10H)-dione **(3n)**

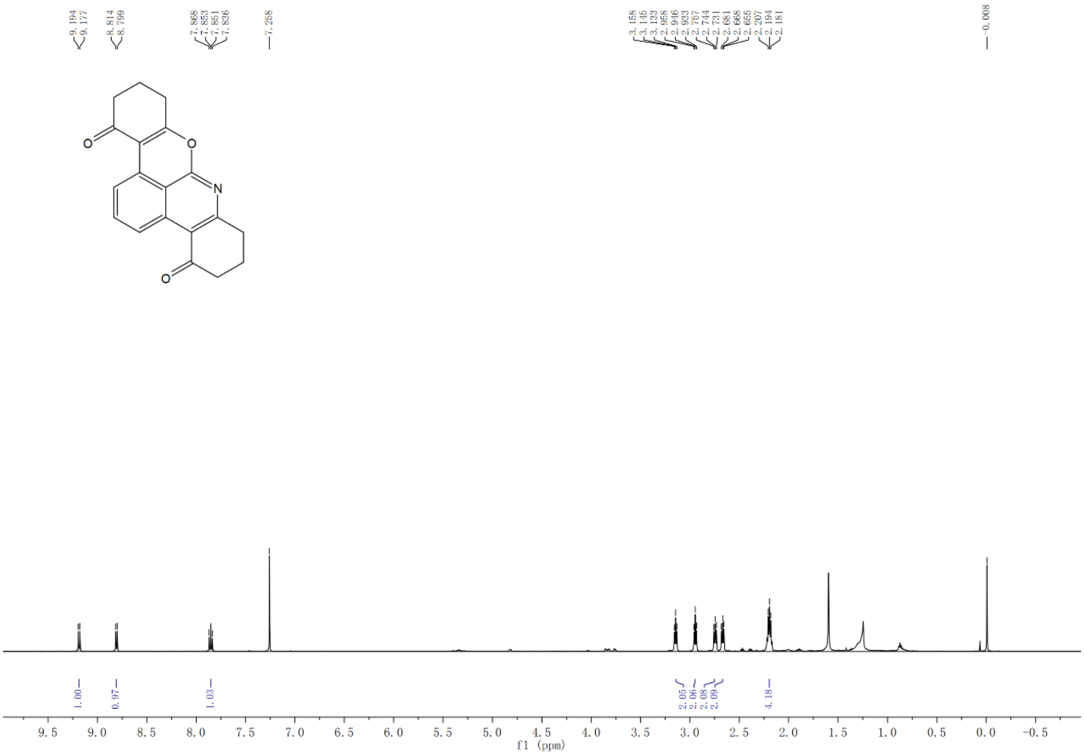

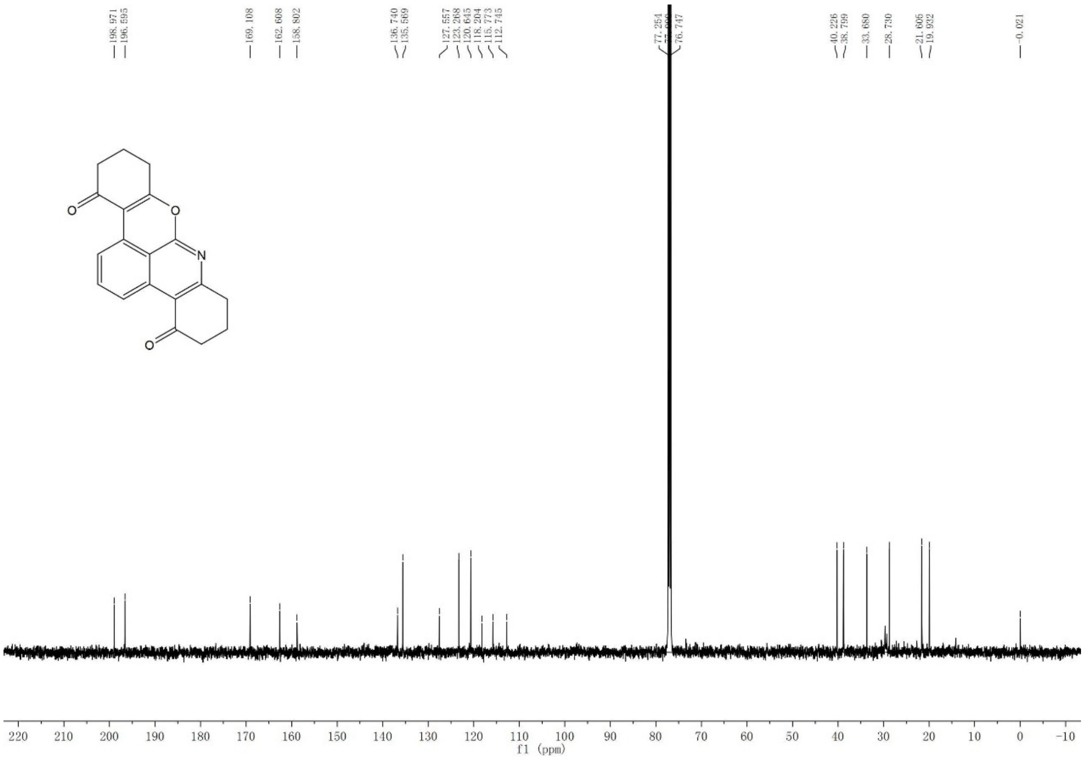

2-methoxy-6,7,11,12-tetrahydrochromeno[2,3,4-gh]phenanthridine-4,13(5H,10H)-dione **(3o)**

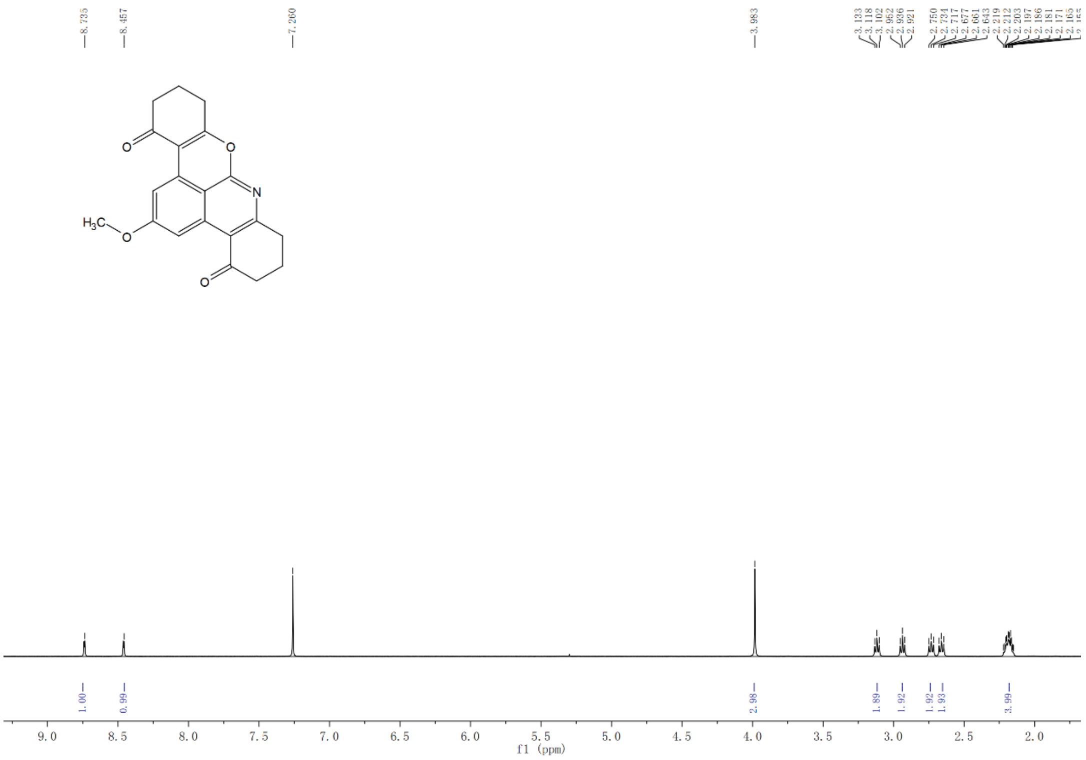

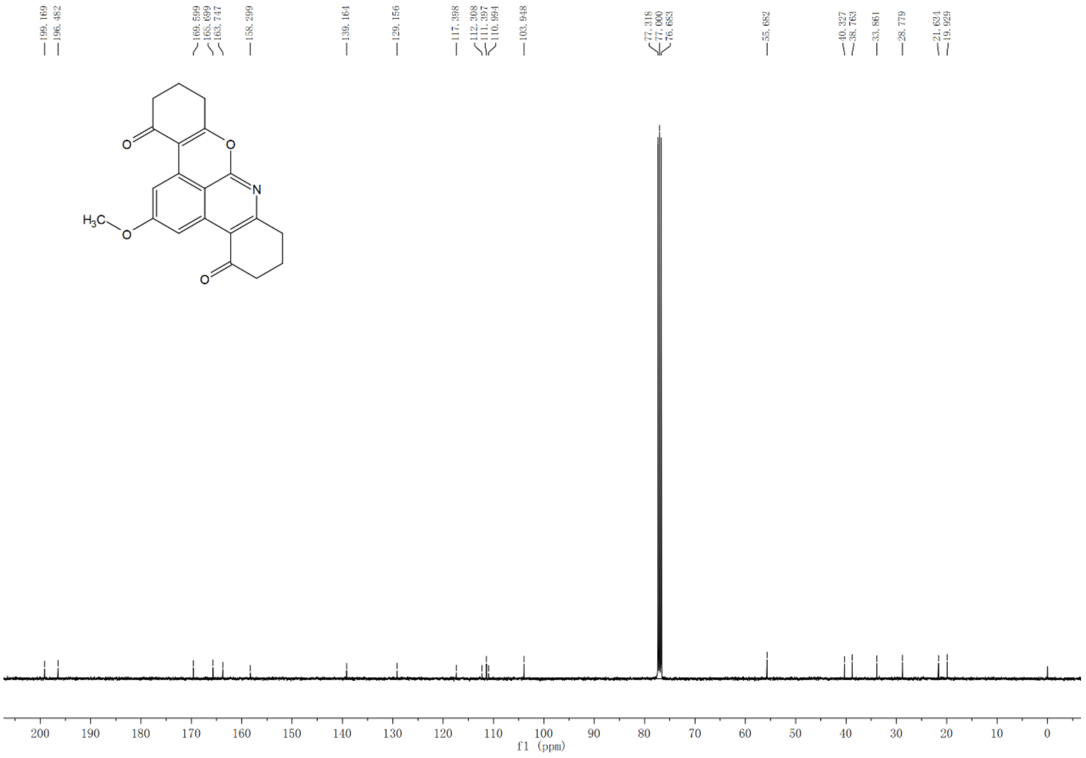

2-methyl-6,7,11,12-tetrahydrochromeno[2,3,4-gh]phenanthridine-4,13(5H,10H)-dione **(3p)**

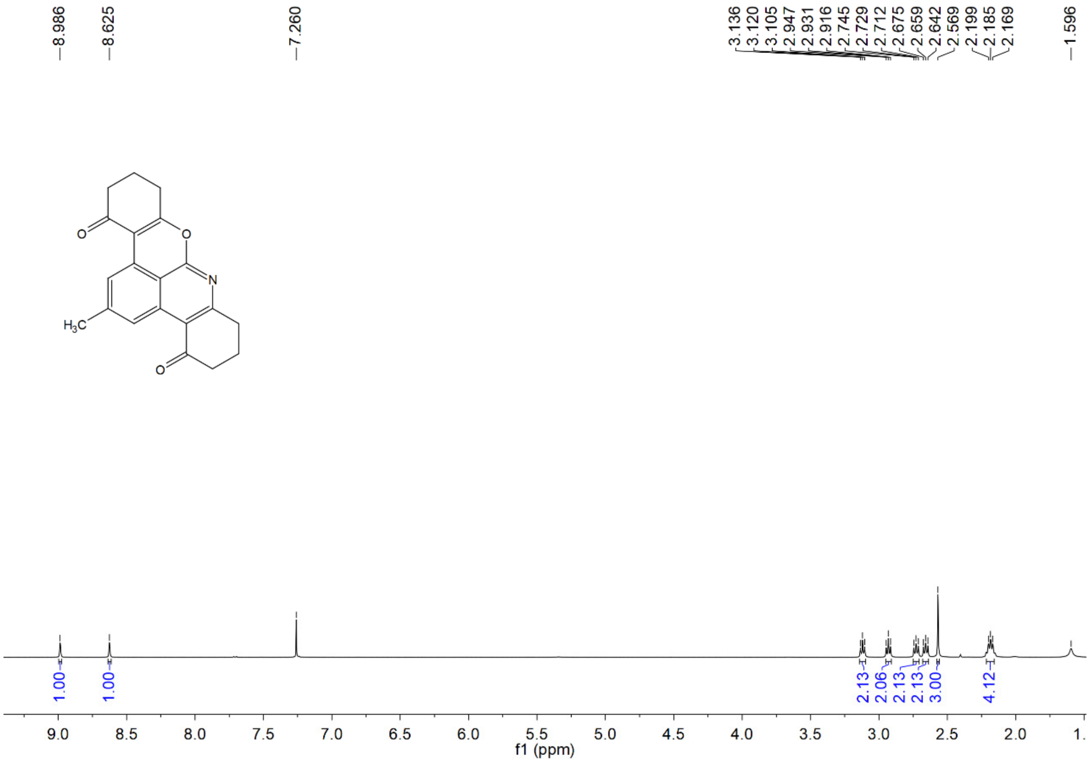

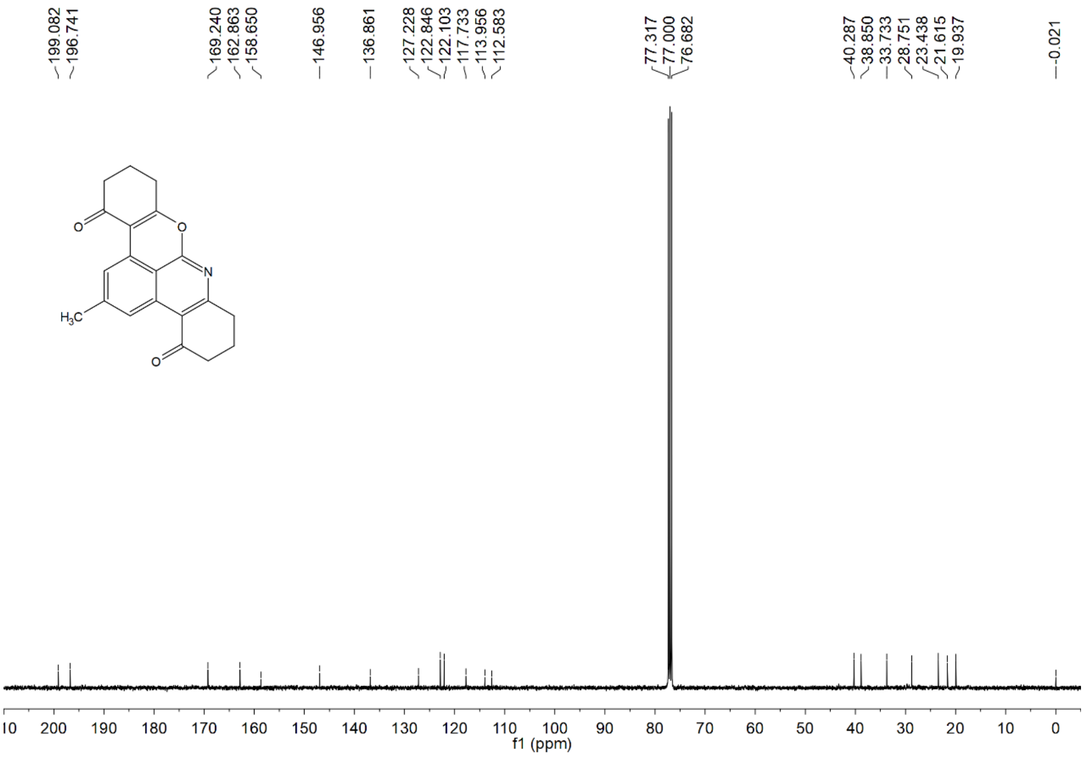

5,6,9,10-tetrahydrocyclopenta[5,6]pyrano[4,3,2-ij]cyclopenta[c]isoquinoline-4,11-Dione **(3q)**

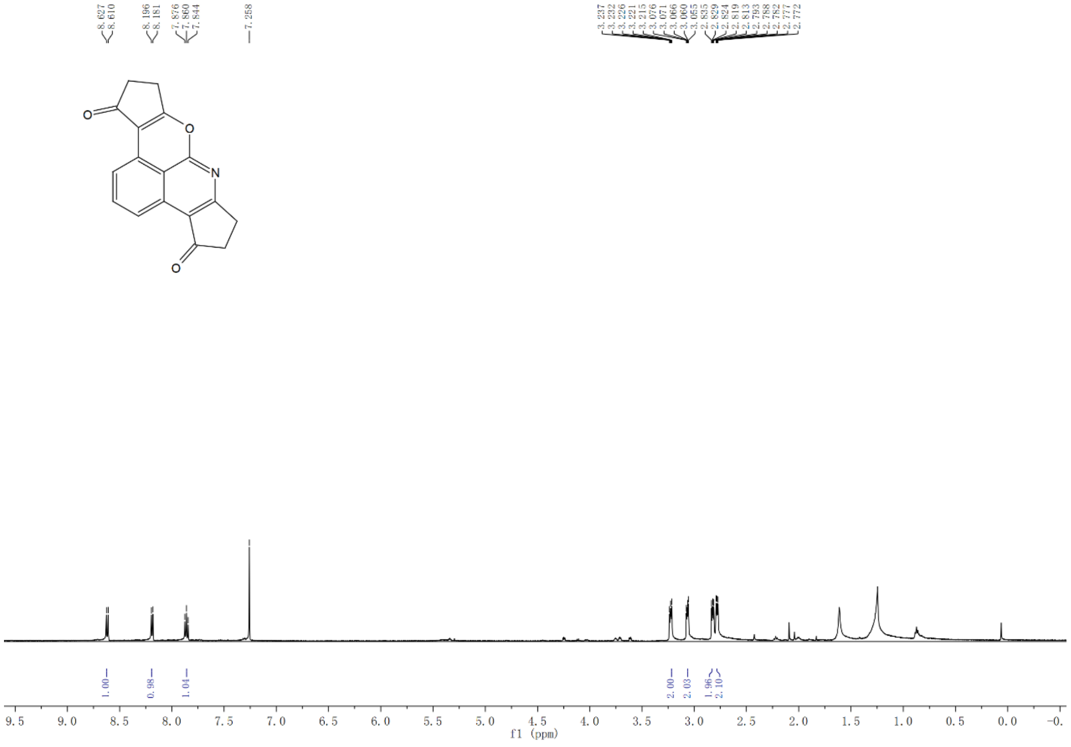

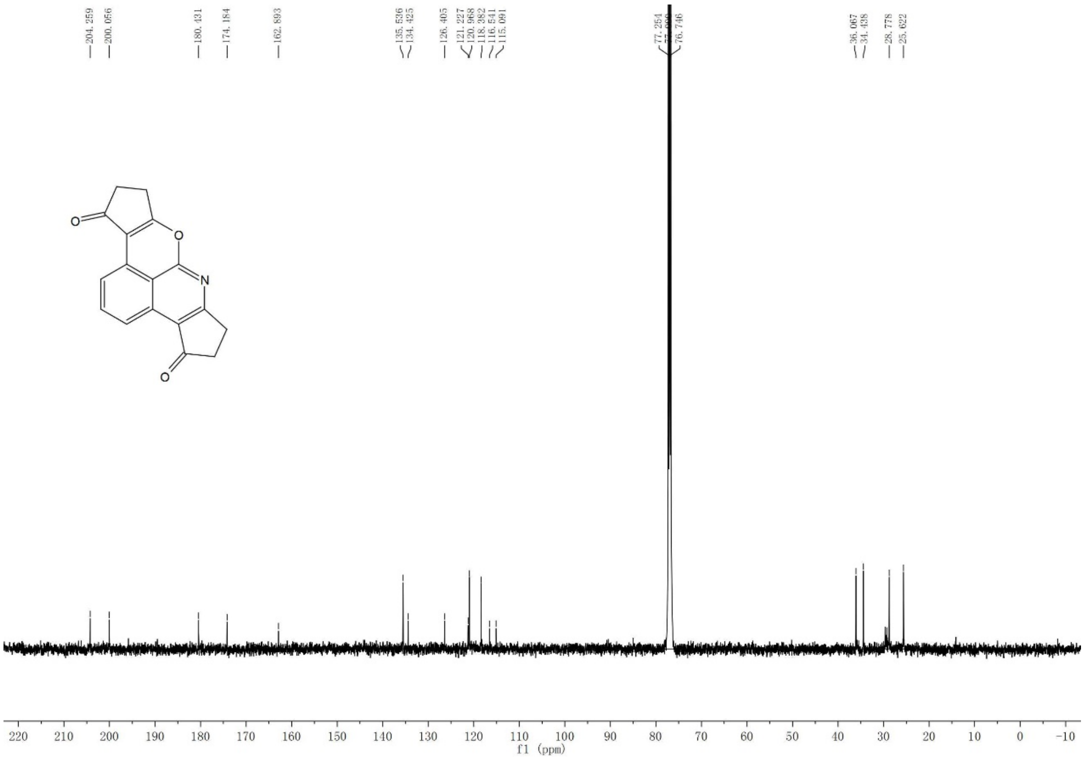

6,11-diphenyl-6,7,11,12-tetrahydrochromeno[2,3,4-gh]phenanthridine-4,13(5H,10H)-dione **(3r)**

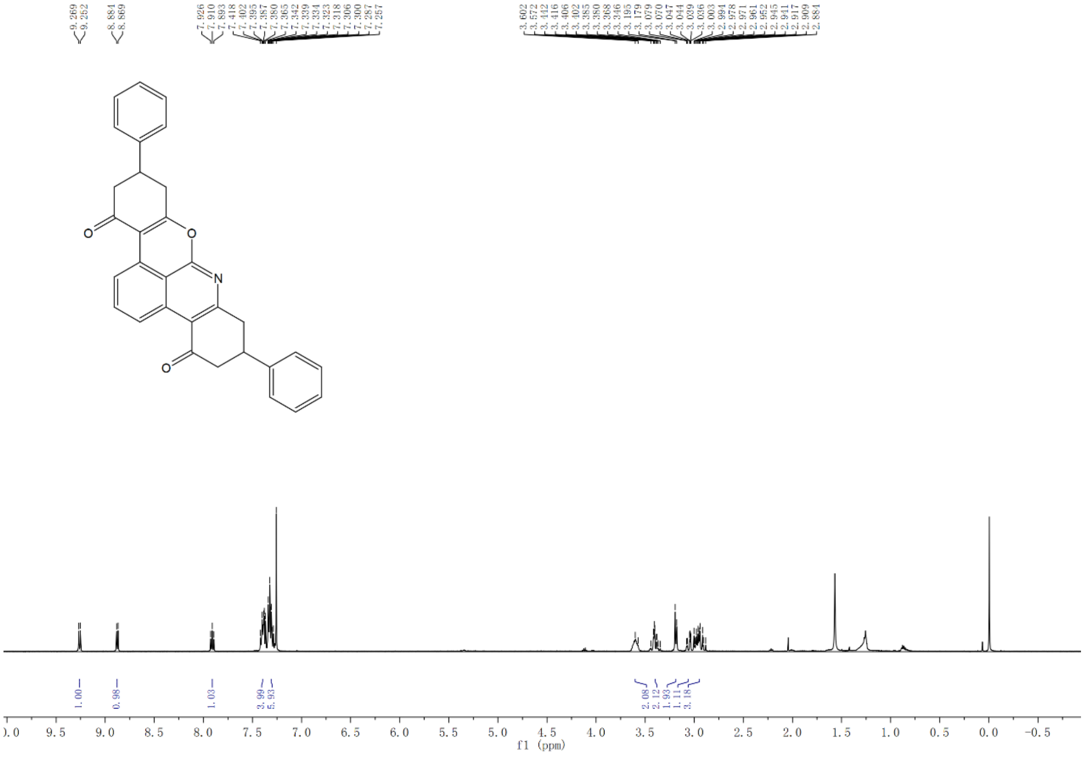

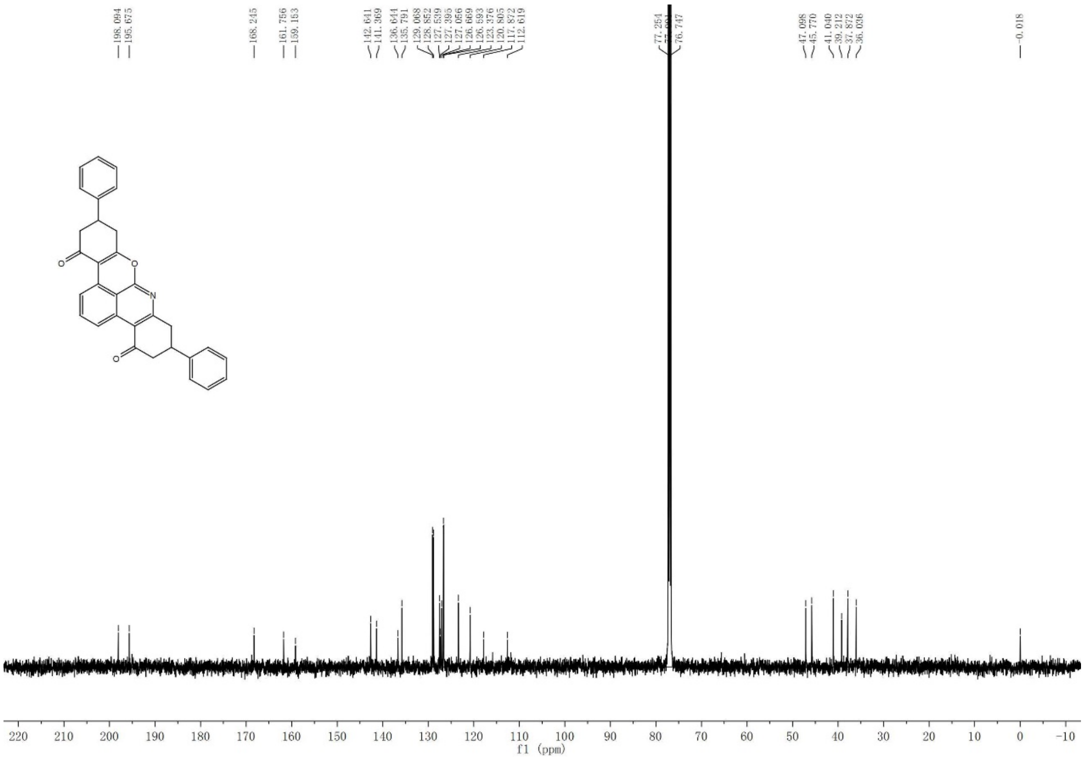

6,11-bis(4-chlorophenyl)-6,7,11,12-tetrahydrochromeno[2,3,4-gh]phenanthridine-4,13(5H,10H)-dione **(3s)**

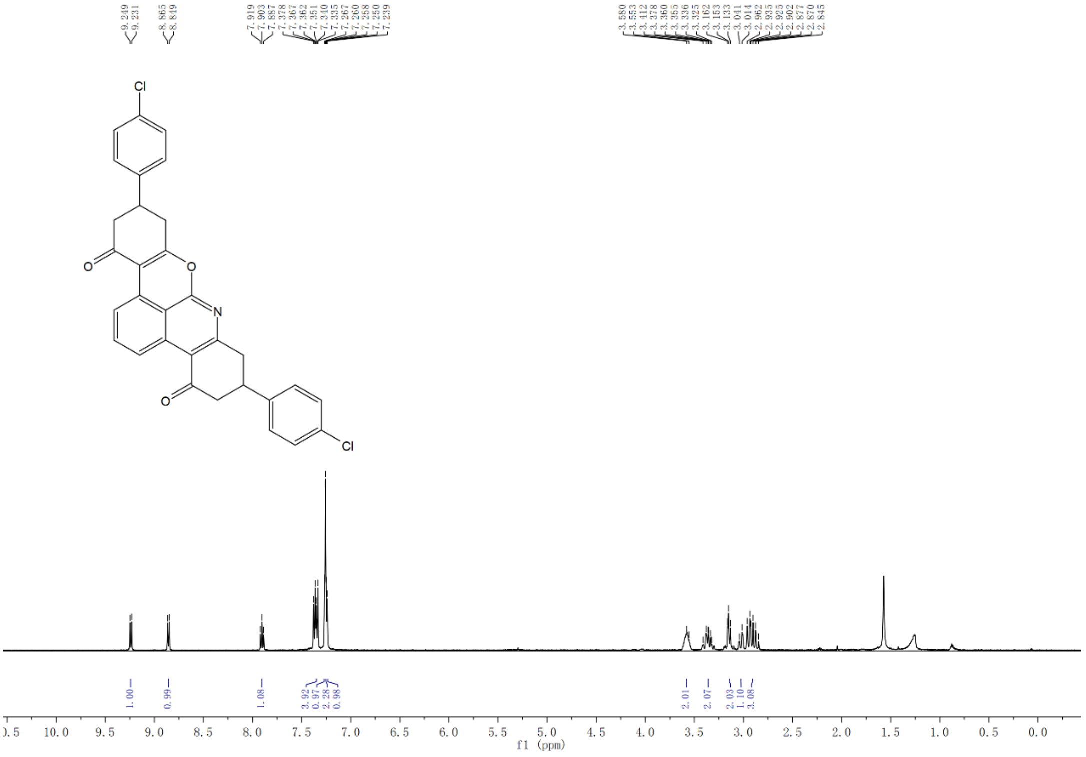

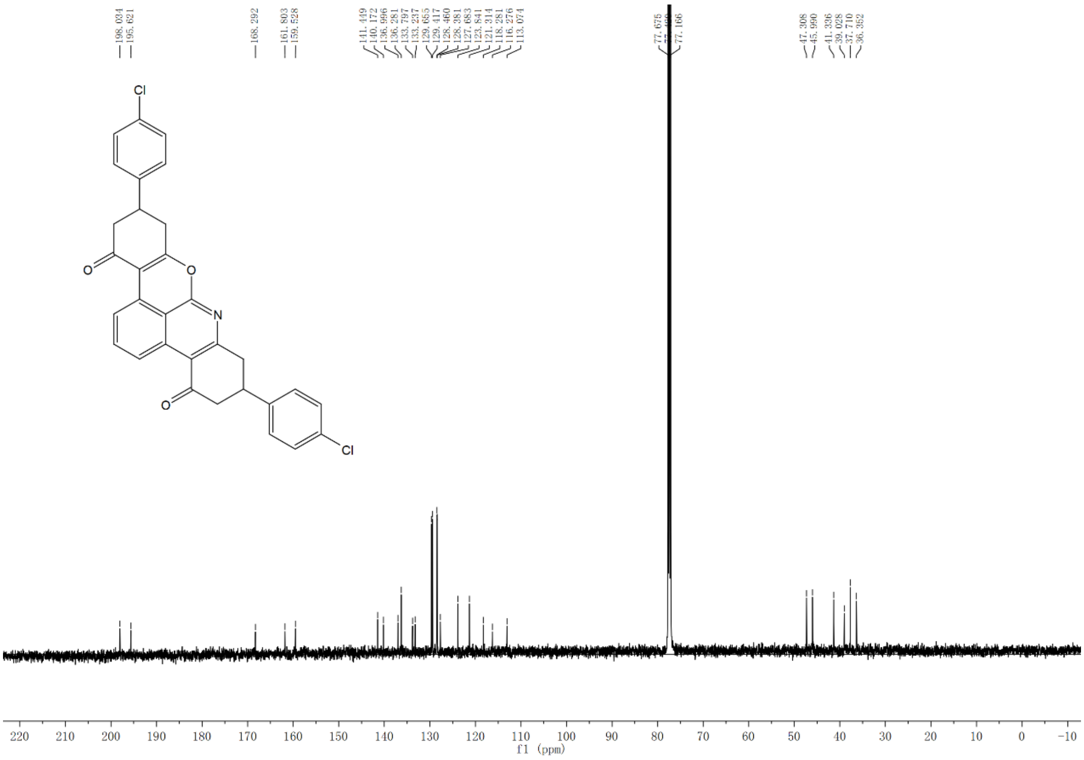

2-butyl-6,11-bis(4-chlorophenyl)-6,7,11,12-tetrahydrochromeno[2,3,4-gh]phenanthridine-4,13(5H,10H)-dione **(3t)**

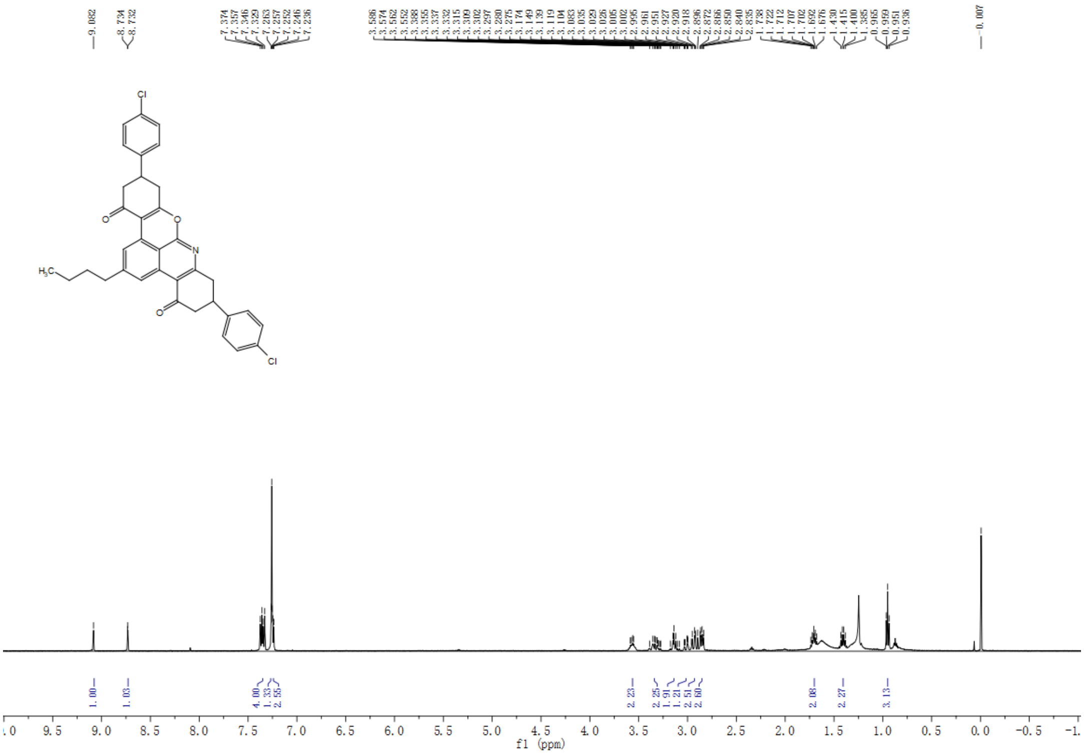

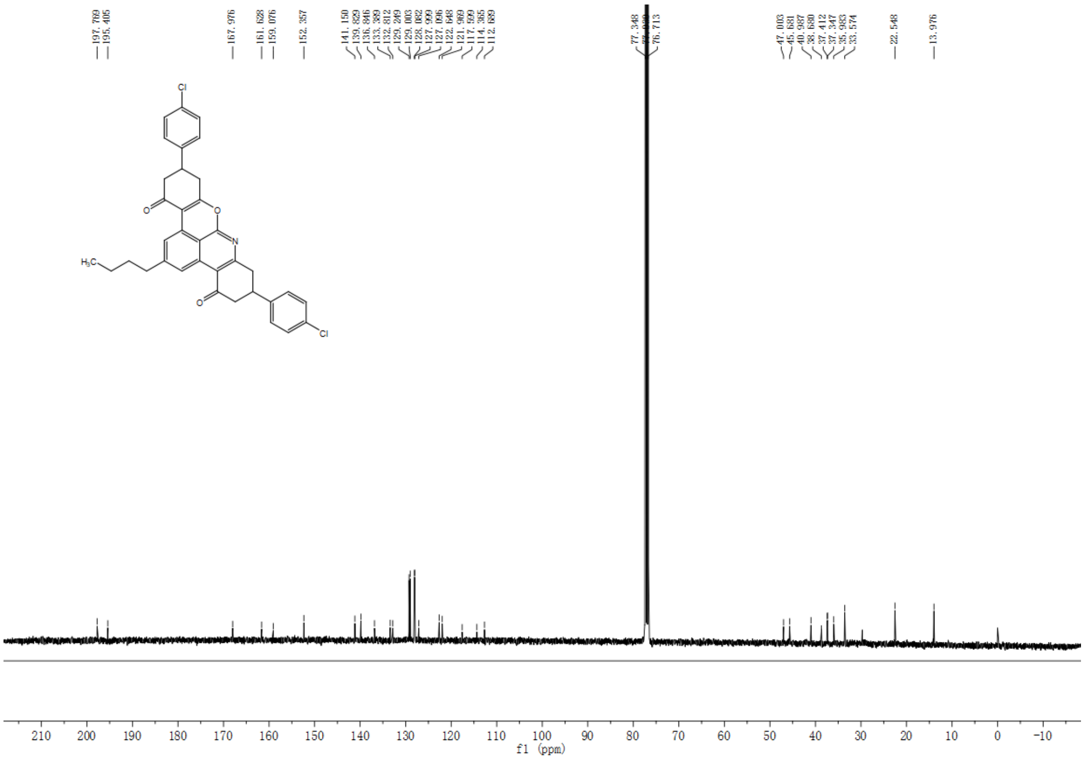

(E)-6,6,11,11-tetramethyl-2-styryl-6,7,11,12-tetrahydrochromeno[2,3,4-gh] phenanthridine-4,13(5H,10H)-dione **(4)**

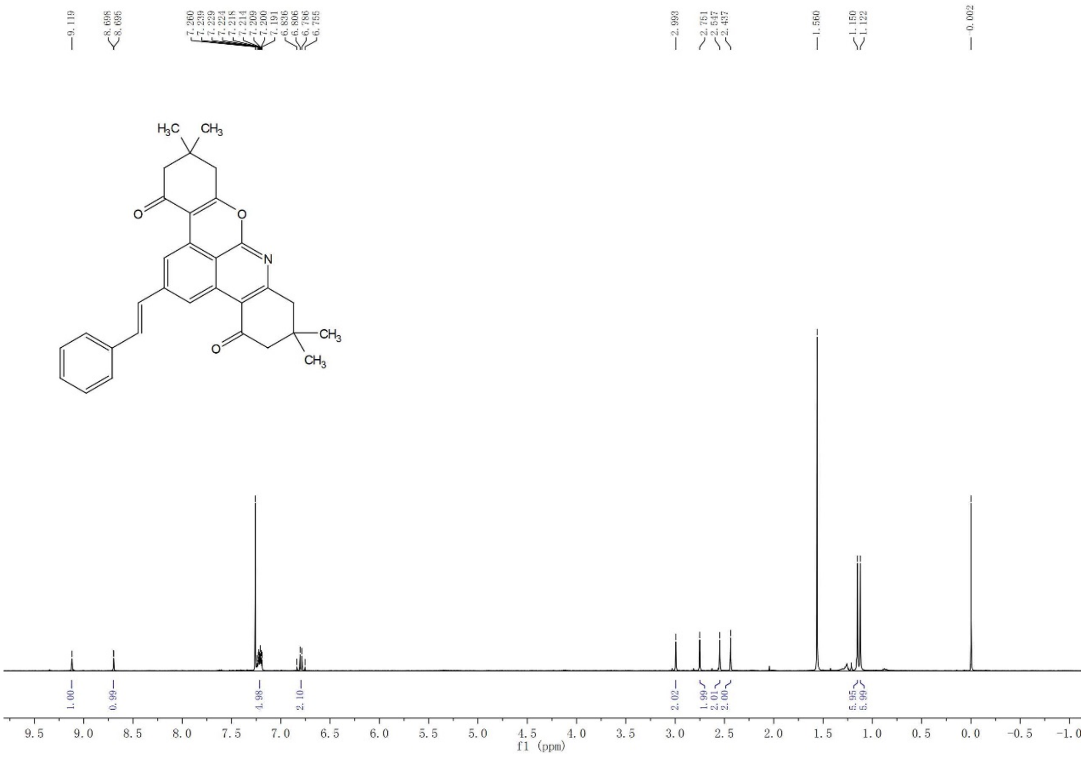

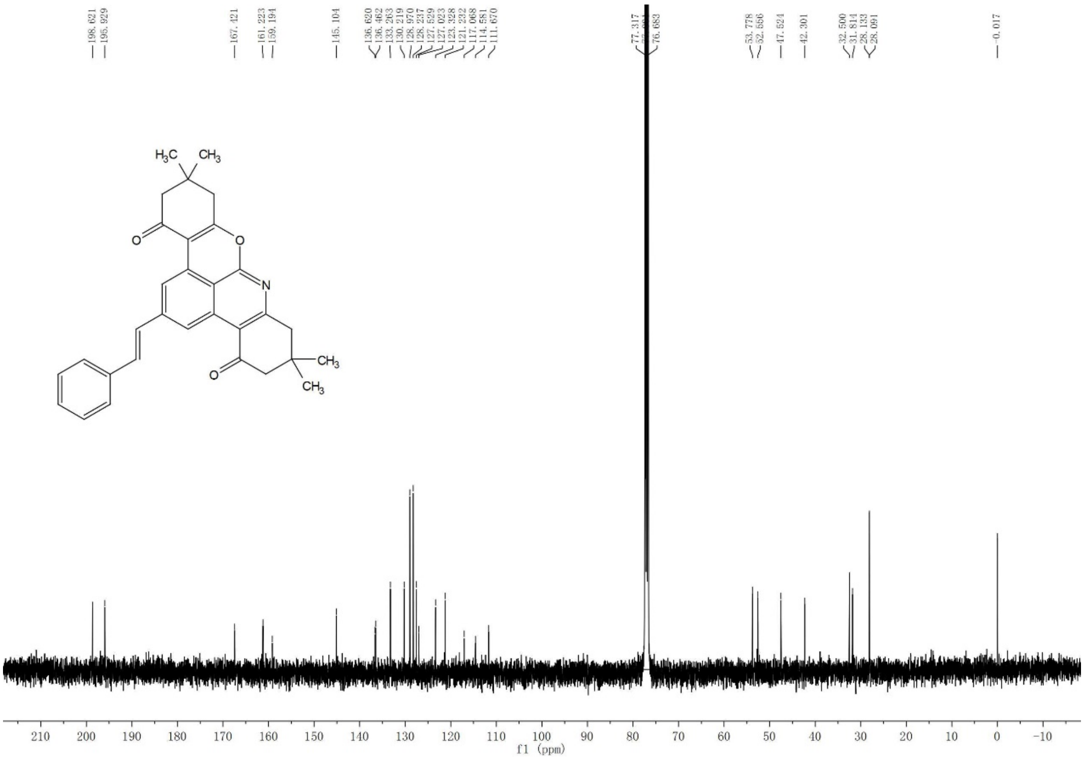

6,6,11,11-tetramethyl-2-(phenylethynyl)-6,7,11,12-tetrahydrochromeno[2,3,4-gh] phenanthridine-4,13(5H,10H)-dione **(5)**

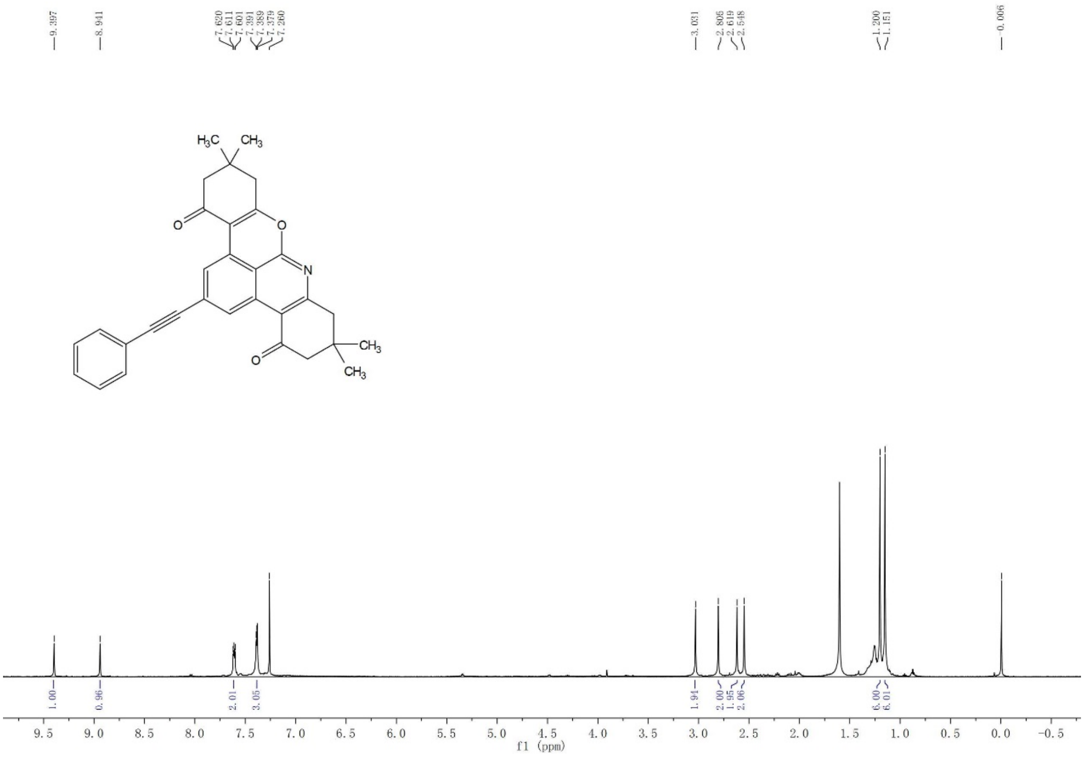

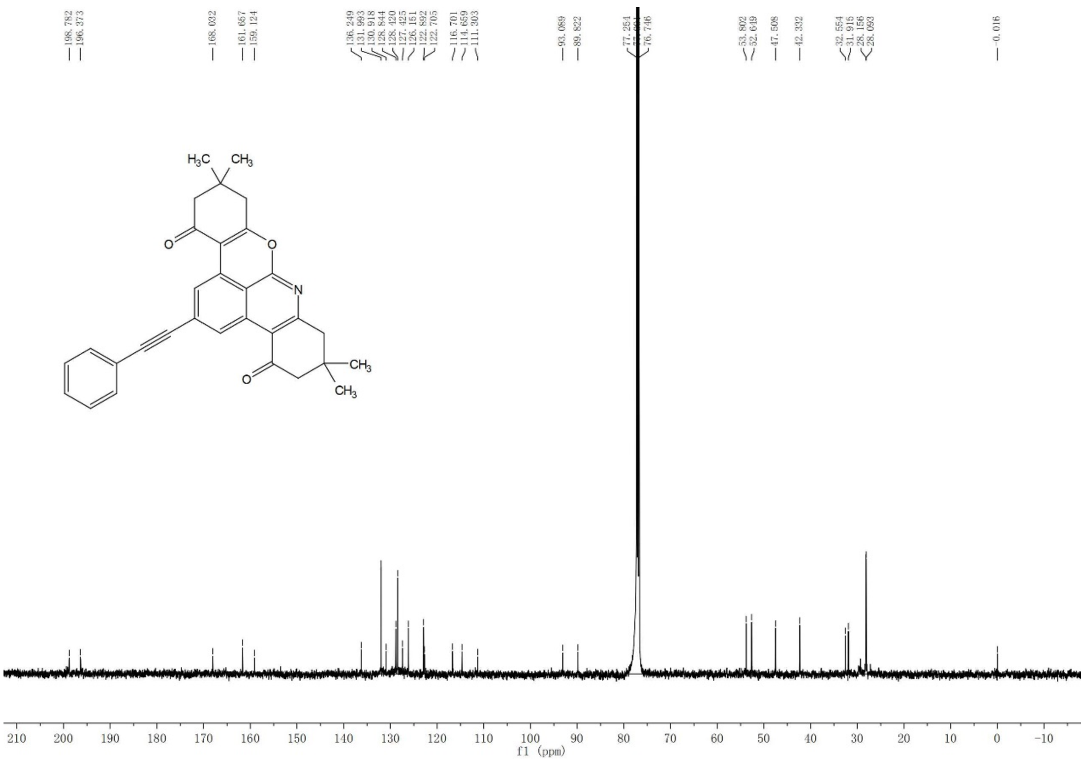

6,6,11,11-tetramethyl-2-(p-tolyl)-6,7,11,12-tetrahydrochromeno[2,3,4-gh] phenanthridine-4,13(5H,10H)-dione **(6)**

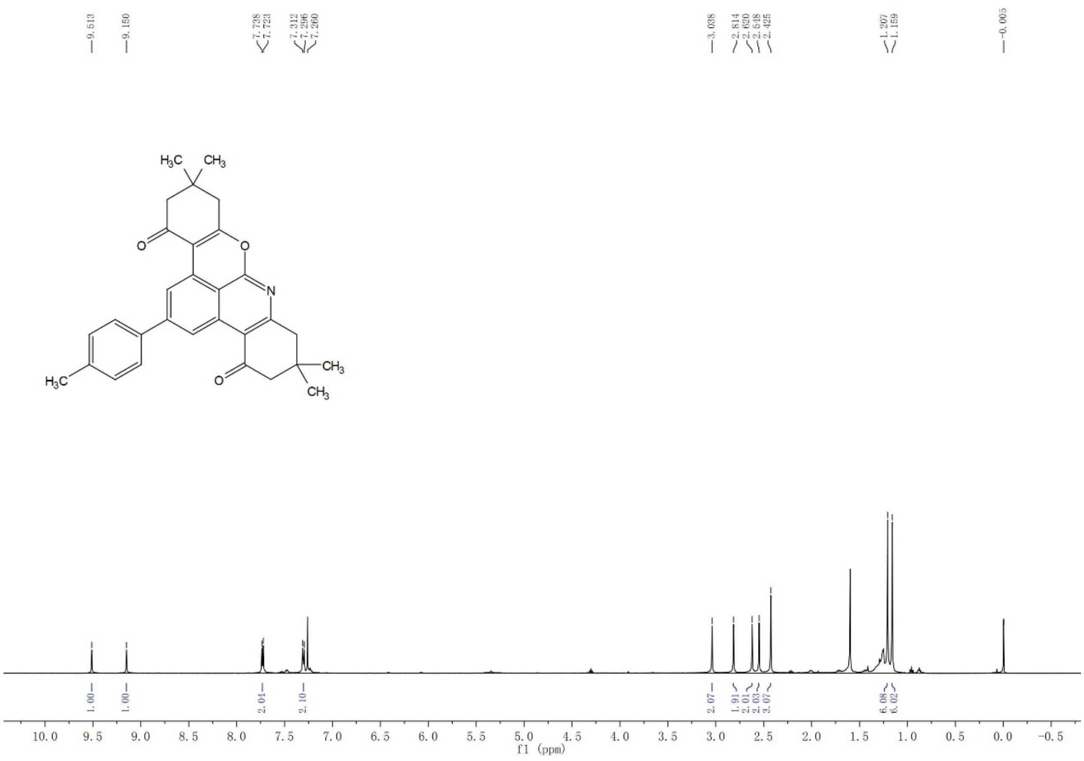

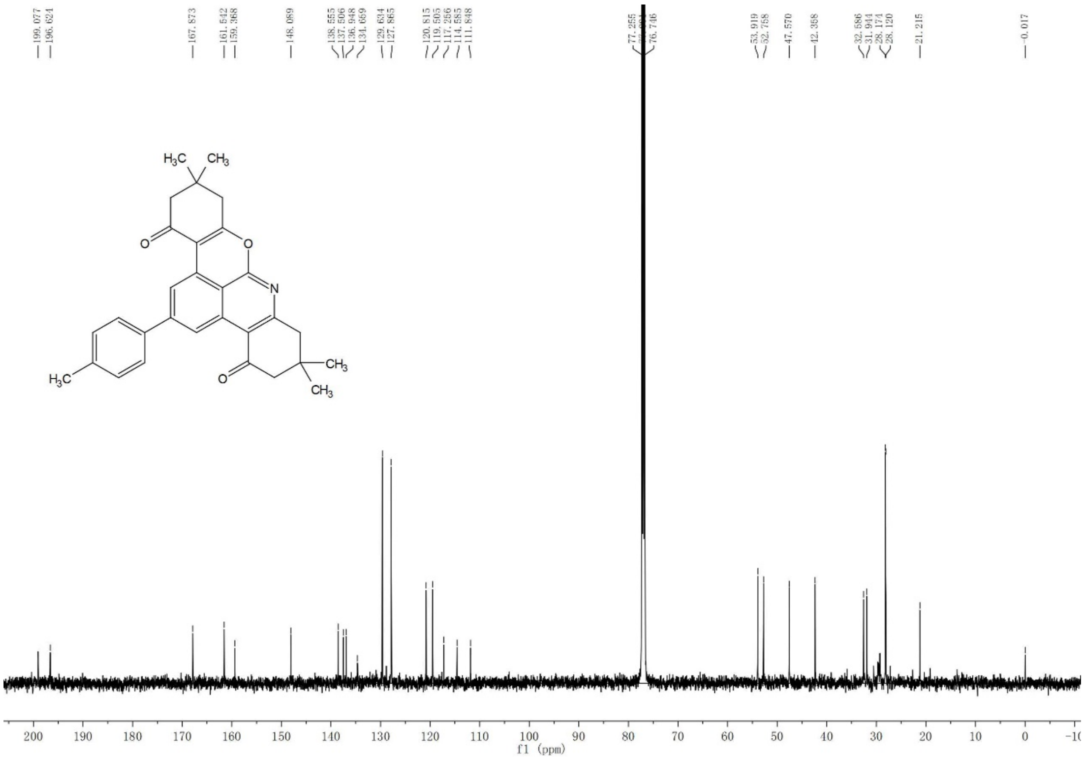

6,6,11,11-tetramethyl-2-(phenyl(p-tolyl)amino)-6,7,11,12-tetrahydrochromeno[2,3,4-gh] phenanthridine-4,13(5H,10H)-dione **(7)**

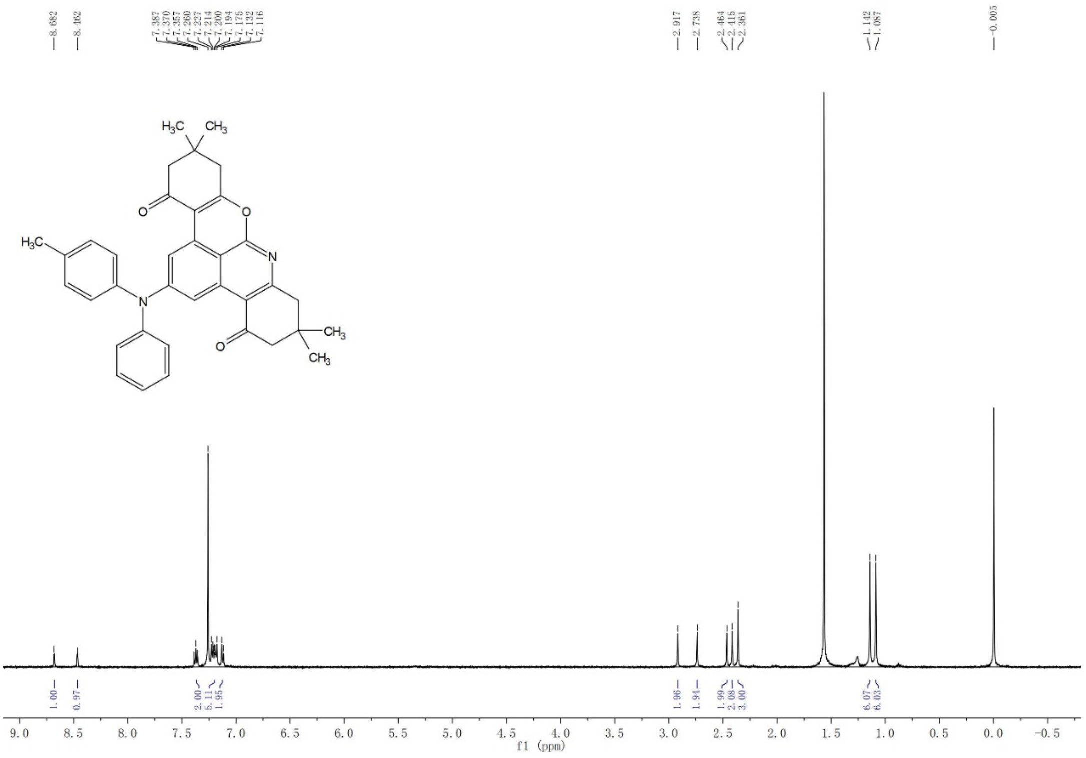

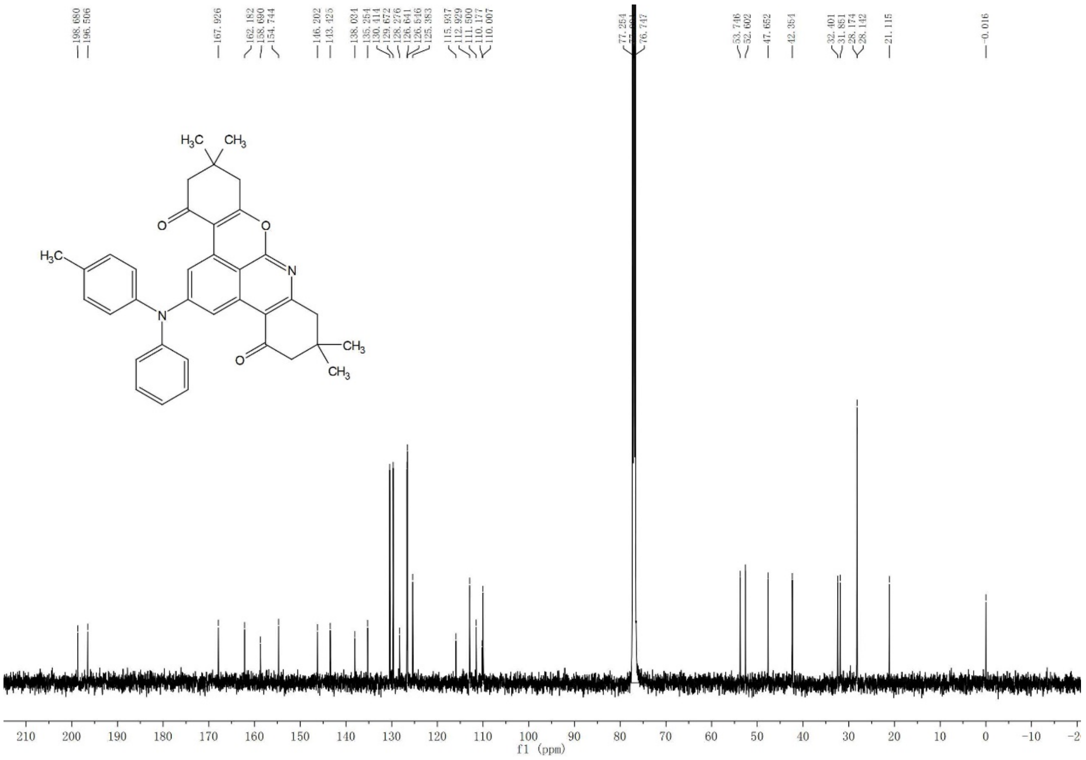

6,6,11,11-tetramethyl-2-(4,4,5,5-tetramethyl-1,3,2-dioxaborolan-2-yl)-6,7,11,12-tetrahydrochromeno[2,3,4-gh] phenanthridine-4,13(5H,10H)-dione **(8)**

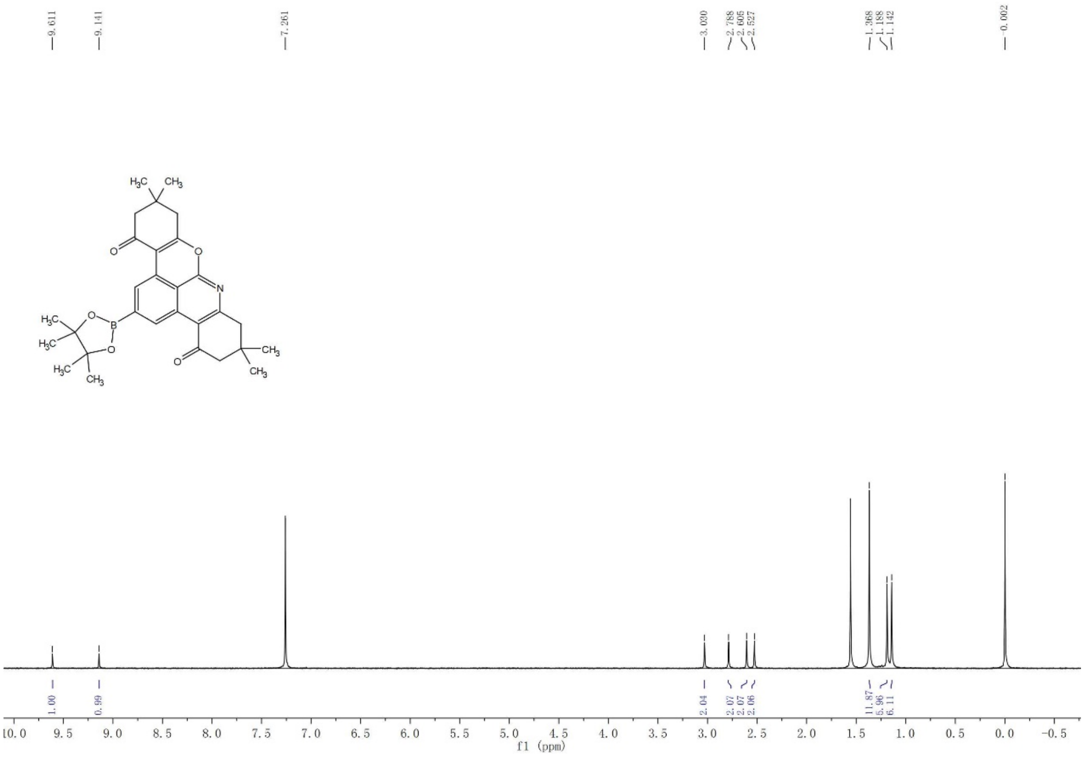

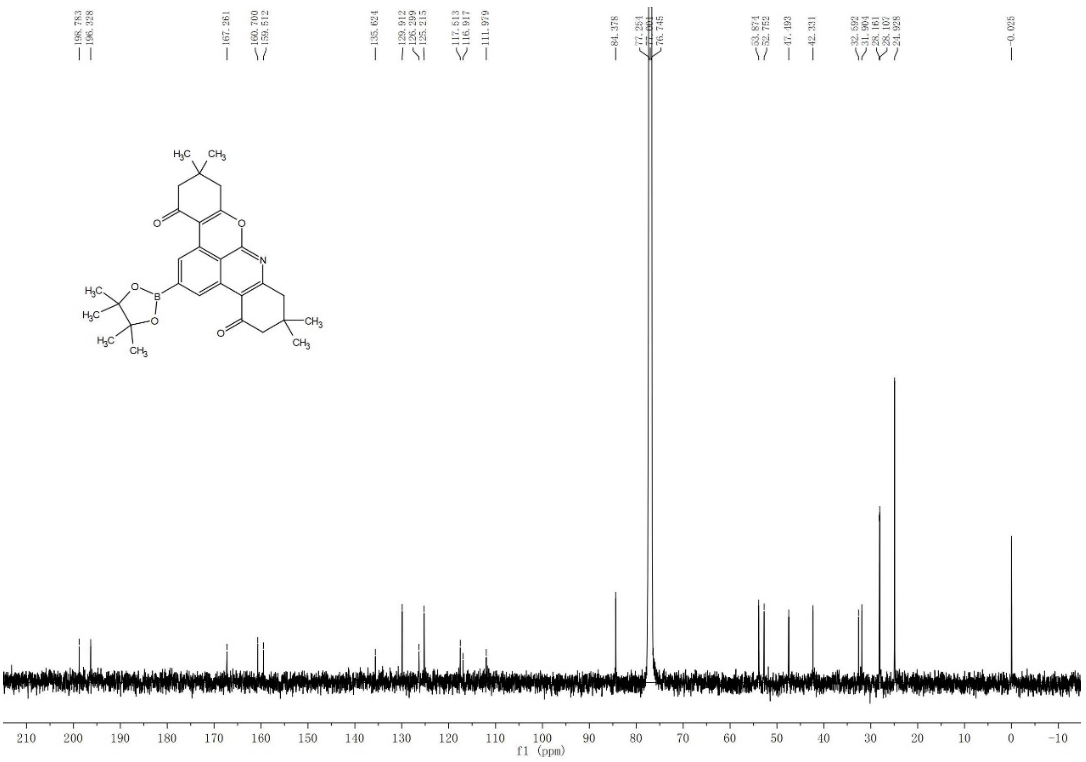

2-bromo-6,6,11,11-tetramethyl-4,5,6,7,10,11,12,13-octahydrochromeno[2,3,4-gh] phenanthridine-4,13-diol **(9)**

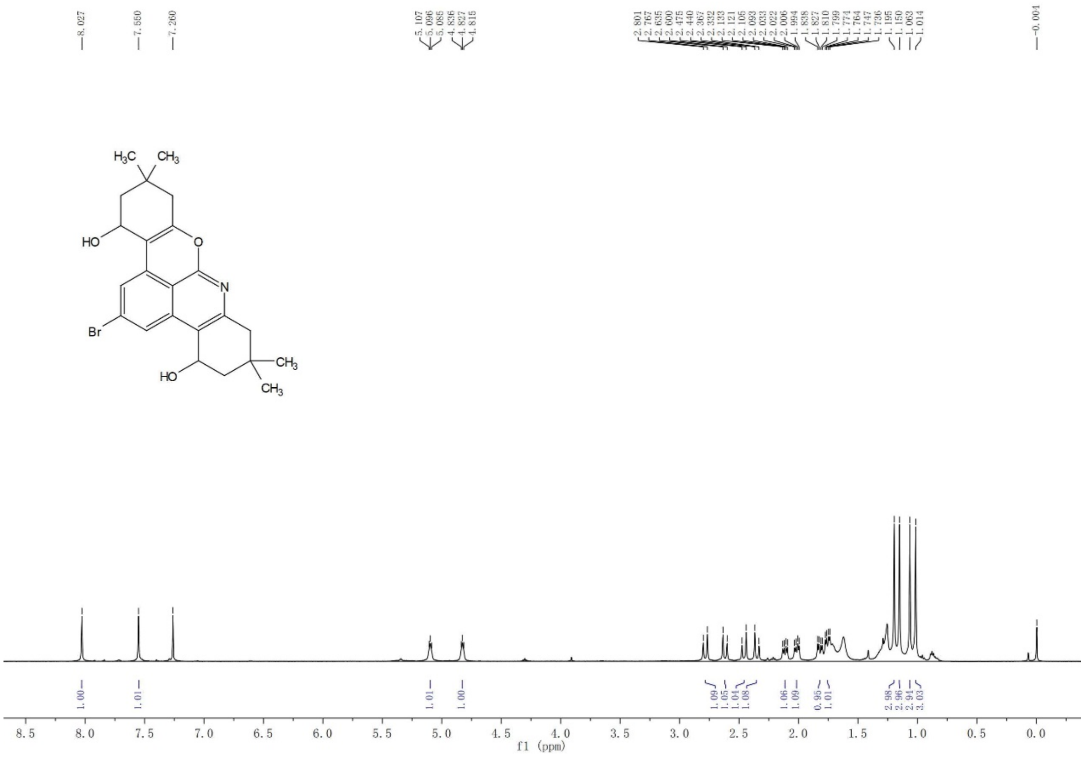

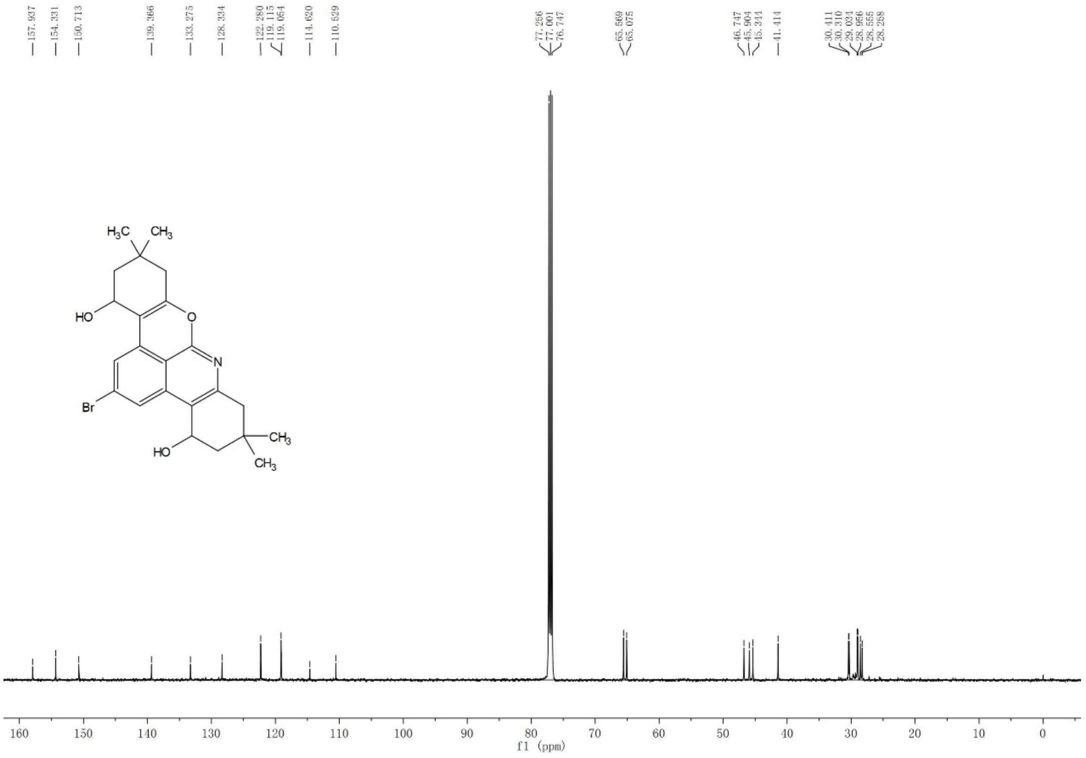
157.937
154.331
150.713
139.366
133.275
128.334
122.280
119.115
119.054
114.620
110.529
77.256
77.001
76.747
65.569
65.075
46.747
45.904
45.311
41.414
30.411
30.310
29.034
28.956
28.555
28.268
H3C
CH3
HO
O
N
Br
CH3
HO
CH3
160 150 140 130 120 110 100 90 80 70 60 50 40 30 20 10 0
f1 (ppm)

S4. X-ray crystal data of compound 3a

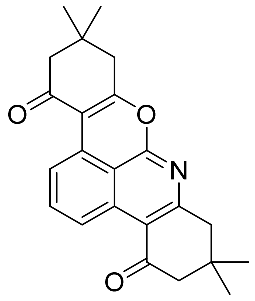

3a

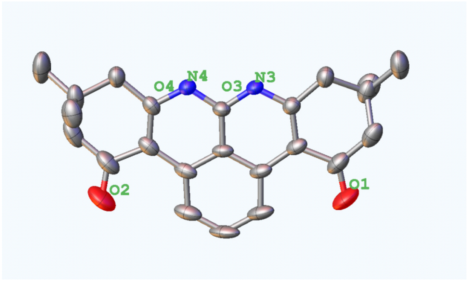

X-ray of **3a**

Table S4. Summary of X-ray crystallographic data for **3a**	
CCDC Number	2132351
Empirical formula	$C_{23}H_{23}NO_3$
Formula weight	363.41
Temperature/K	300.01
Crystal system	orthorhombic
Space group	Pbca
a/Å	8.123
b/Å	19.018
c/Å	24.245
α/°	90
β/°	90
γ/°	90
Volume/Å^3	3745.4
Z	8
ρ_{calc}g/cm^3	1.289
μ/mm^{-1}	0.087
F(000)	1544.0
Crystal size/mm^3	0.22 × 0.21 × 0.2
Radiation	MoKα (λ = 0.71076)
2Θ range for data collection/°	5.706 to 55.148
Index ranges	$-10 \leq h \leq 10$, $-24 \leq k \leq 22$, $-31 \leq l \leq 31$
Reflections collected	72515
Independent reflections	4320 [R_{int} = 0.1268, R_{sigma} = 0.0451]
Data/restraints/parameters	4320/0/248
Goodness-of-fit on F^2	1.076

Final R indexes [I>=2σ (I)]	R_1 = 0.0874, wR_2 = 0.2376
Final R indexes [all data]	R_1 = 0.1544, wR_2 = 0.2798
Largest diff. peak/hole / e $Å^{-3}$	0.43/-0.30

S5. HRMS spectra

6,6,11,11-tetramethyl-6,7,11,12-tetrahydrochromeno[2,3,4-gh]phenanthridine-4,13(5H,10H)-dione (**3a**), HRMS (ESI) m/z: $[M+H]^+$ calcd for $C_{23}H_{24}NO_3^+$ 362.1751; found 362.1753.

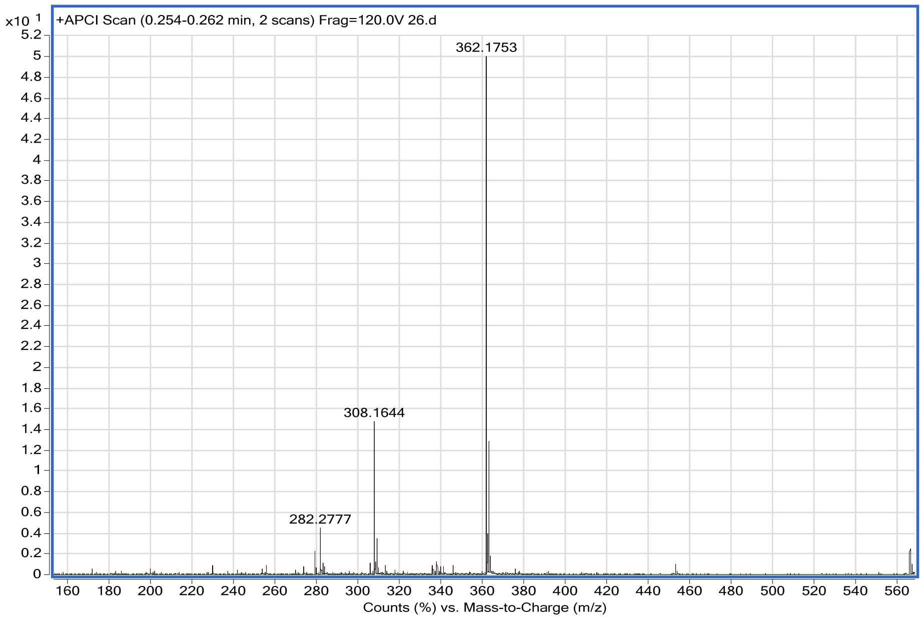

2,6,6,11,11-pentamethyl-6,7,11,12-tetrahydrochromeno[2,3,4-gh]phenanthridine-4,13(5H,10H)-dione (**3b**), HRMS (ESI) m/z: $[M+H]^+$ calcd for $C_{24}H_{26}NO_3^+$ 376.1907; found 376.1906.

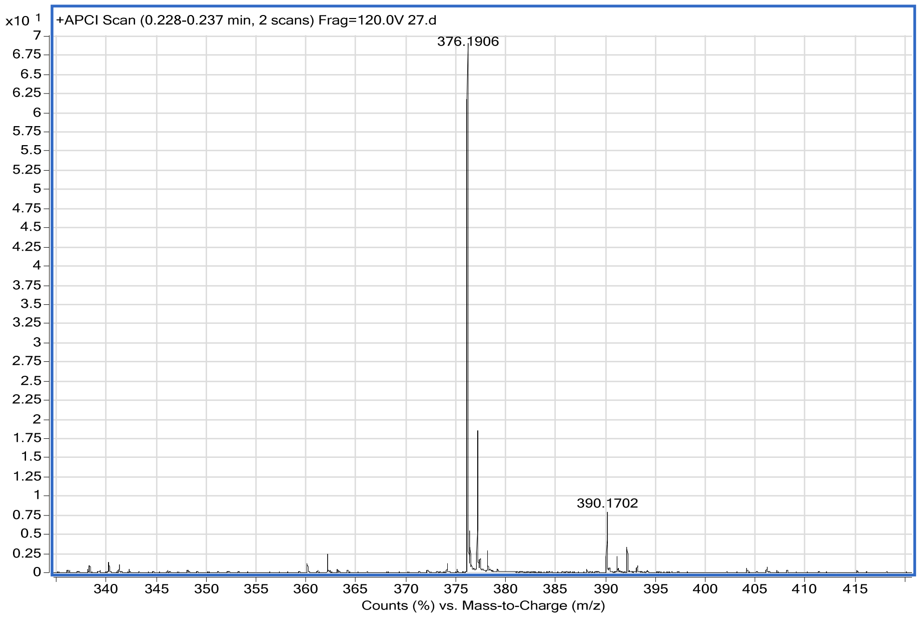

2-butyl-6,6,11,11-tetramethyl-6,7,11,12-tetrahydrochromeno[2,3,4-gh]phenanthridine-4,13(5H,10H)-dione (**3c**), HRMS (ESI) m/z: $[M+H]^+$ calcd for $C_{27}H_{32}NO_3^+$ 418.2377; found 418.2368.

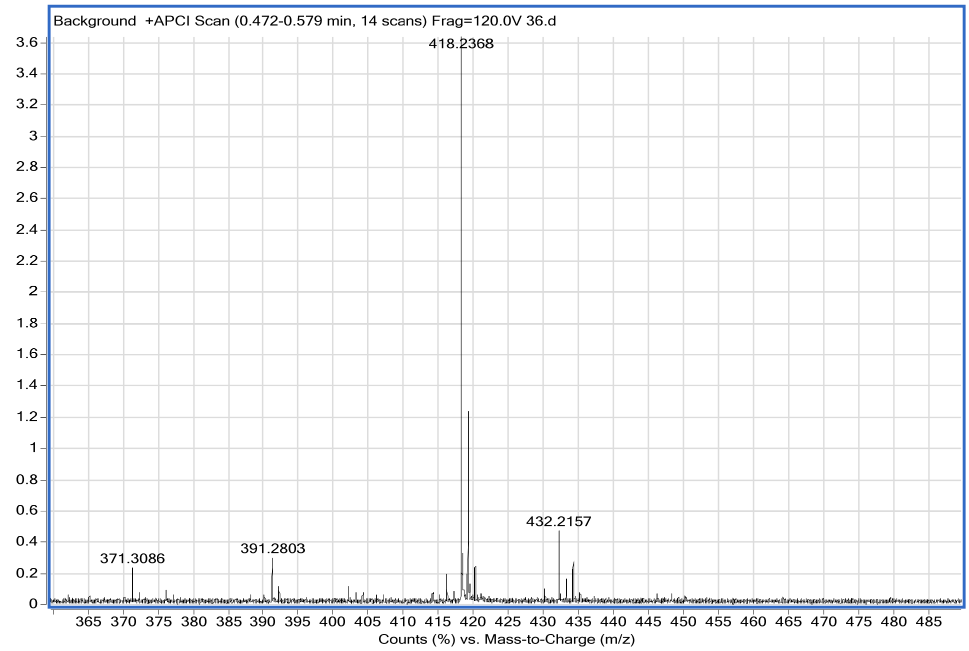

2-methoxy-6,6,11,11-tetramethyl-6,7,11,12-tetrahydrochromeno[2,3,4-gh]phenanthridine-4,13(5H,10H)-dione **(3d)**, HRMS (ESI) m/z: $[M+H]^+$ calcd for $C_{24}H_{26}NO_4^+$ 392.1856; found 392.1853.

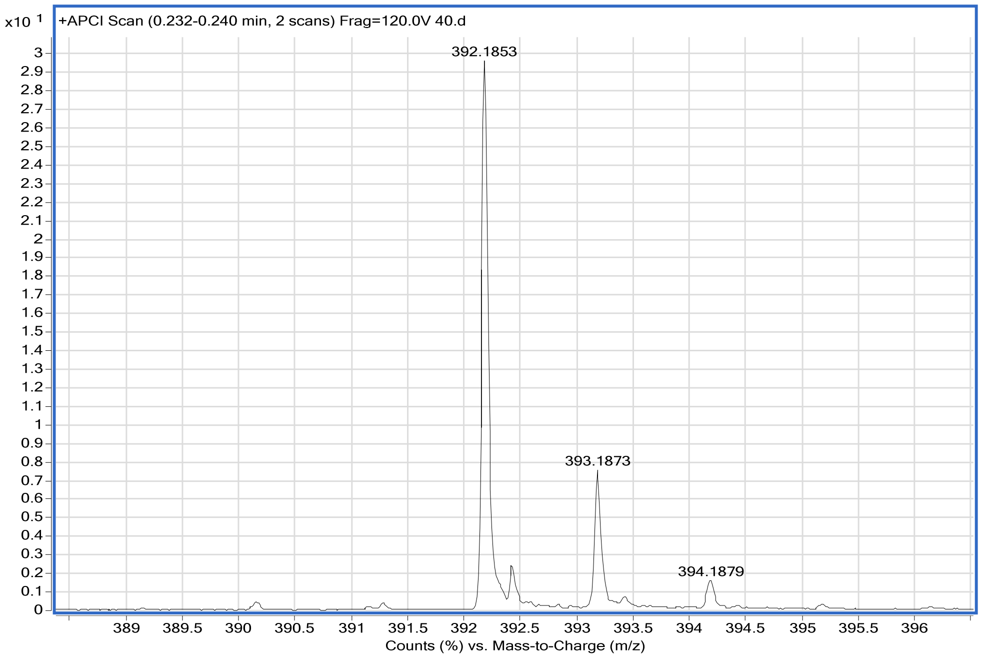

6,6,11,11-tetramethyl-2-(methylthio)-6,7,11,12-tetrahydrochromeno[2,3,4-gh]phenanthridine-4,13(5H,10H)-dione **(3e)**, HRMS (ESI) m/z: $[M+H]^+$ calcd for $C_{24}H_{26}NO_3S^+$ 408.1628; found 408.1625.

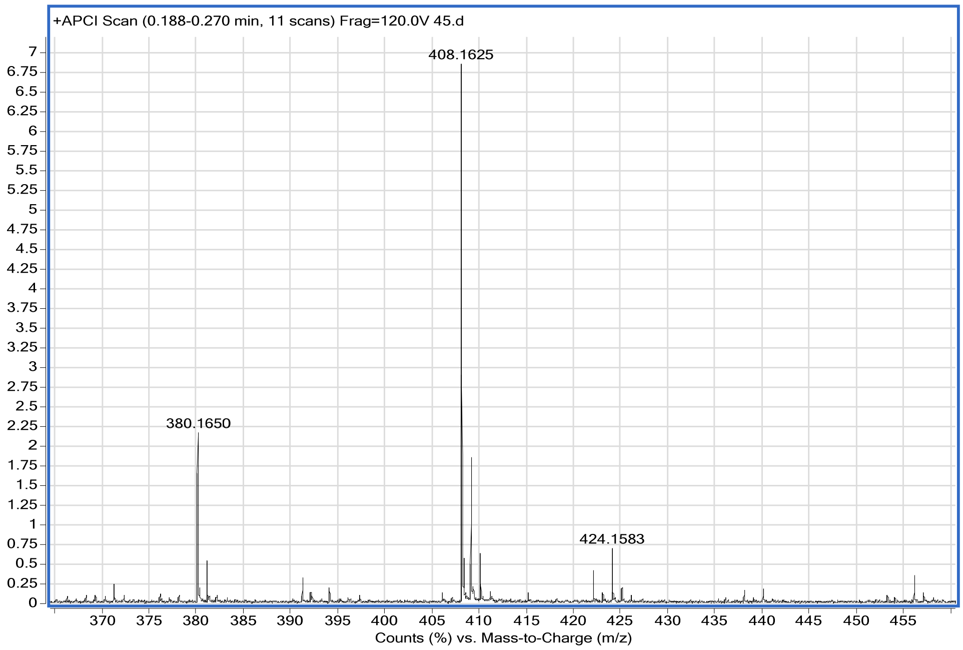

2-fluoro-6,6,11,11-tetramethyl-6,7,11,12-tetrahydrochromeno[2,3,4-gh]phenanthridine-4,13(5H,10H)-dione (**3f**), HRMS (ESI) m/z: $[M+H]^+$ calcd for $C_{23}H_{23}FNO_3^+$ 380.1656; found 380.1665.

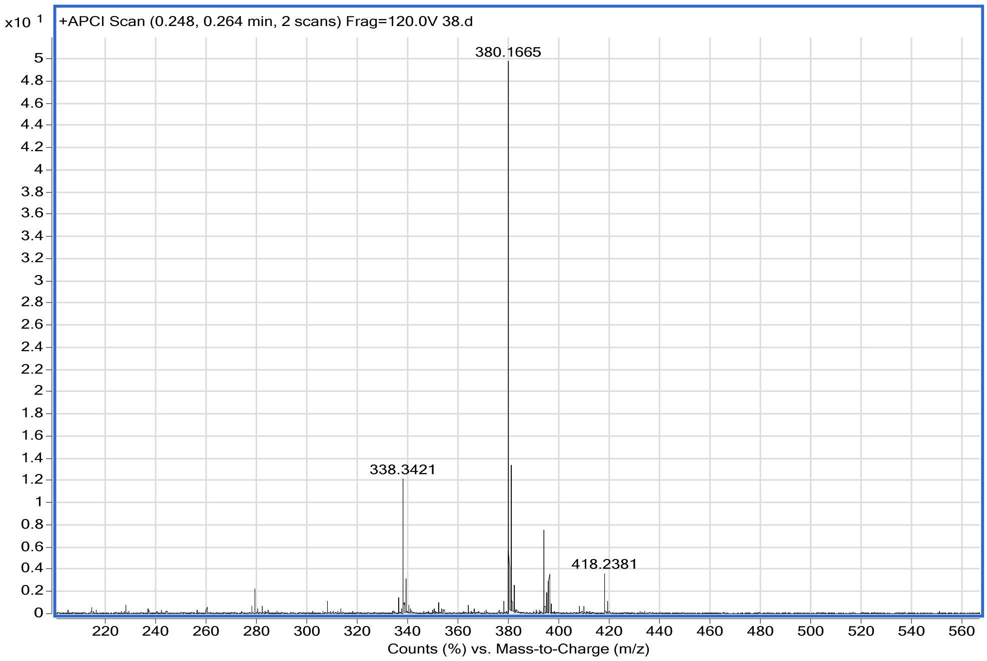

2-chloro-6,6,11,11-tetramethyl-6,7,11,12-tetrahydrochromeno[2,3,4-gh]phenanthridine-4,13(5H,10H)-dione (**3g**), HRMS (ESI) m/z: $[M+H]^+$ calcd for $C_{23}H_{23}ClNO_3^+$ 396.1361; found 396.1369.

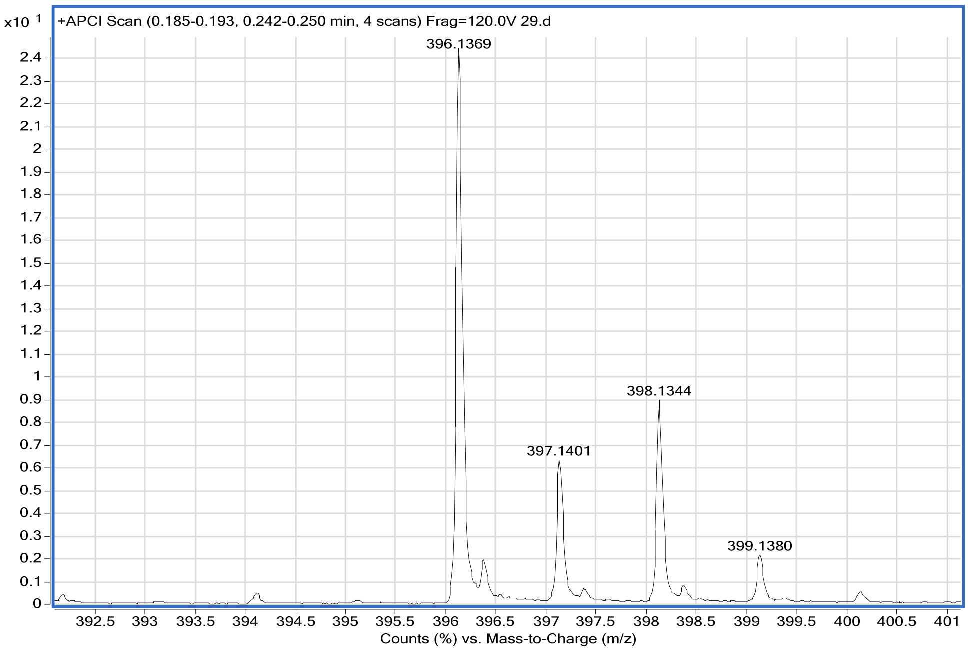

2-bromo-6,6,11,11-tetramethyl-6,7,11,12-tetrahydrochromeno[2,3,4-gh]phenanthridine-4,13(5H,10H)-dione (**3h**), HRMS (ESI) m/z: $[M+H]^+$ calcd for $C_{23}H_{23}BrNO_3^+$ 440.0856; found 440.0853.

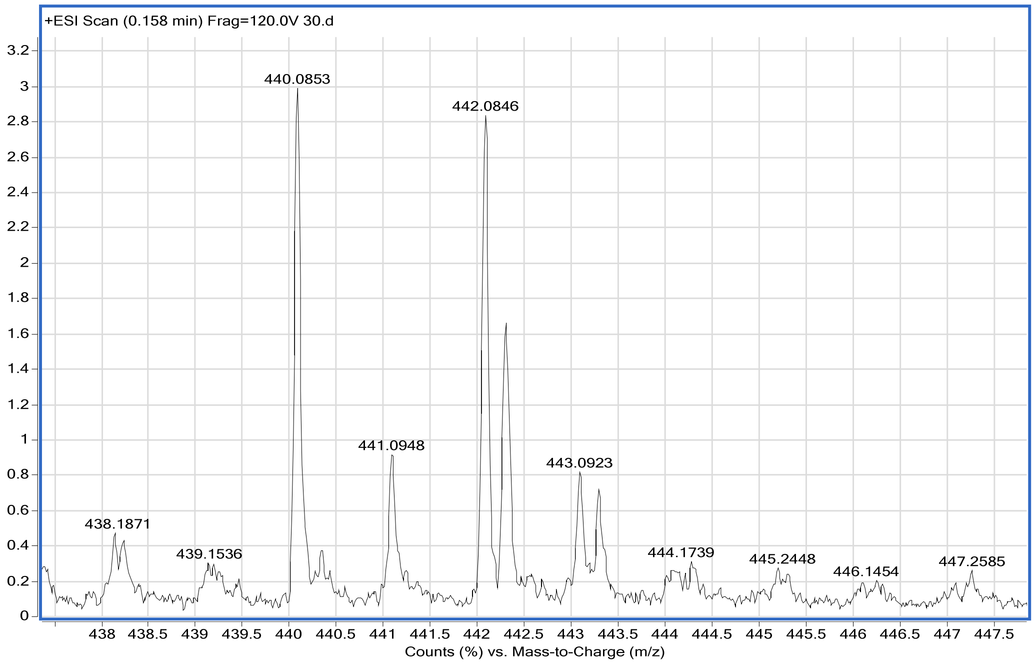

6,6,11,11-tetramethyl-2-(trifluoromethyl)-6,7,11,12-tetrahydrochromeno[2,3,4-gh]phenanthridine-4,13(5H,10H)-dione (**3i**), HRMS (ESI) m/z: $[M+H]^+$ calcd for $C_{24}H_{23}F_3NO_3^+$ 430.1625; found 430.1624.

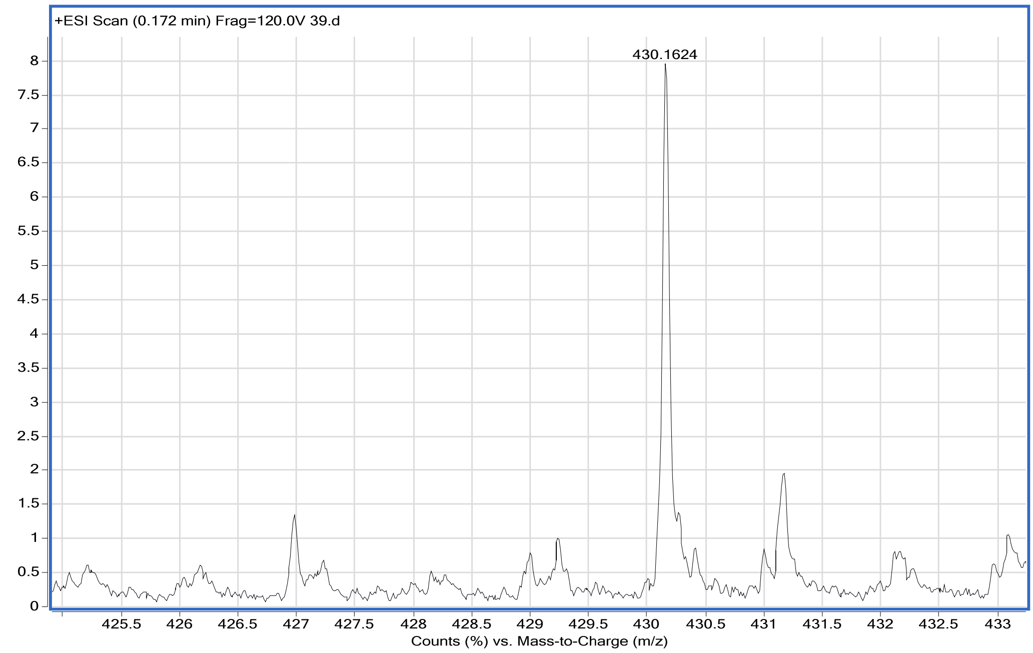

6,6,11,11-tetramethyl-2-phenyl-6,7,11,12-tetrahydrochromeno[2,3,4-gh]phenanthridine-4,13(5H,10H)-dione (**3j**), HRMS (ESI) m/z: $[M+H]^+$ calcd for $C_{29}H_{28}NO_3^+$ 438.2064; found 438.2066.

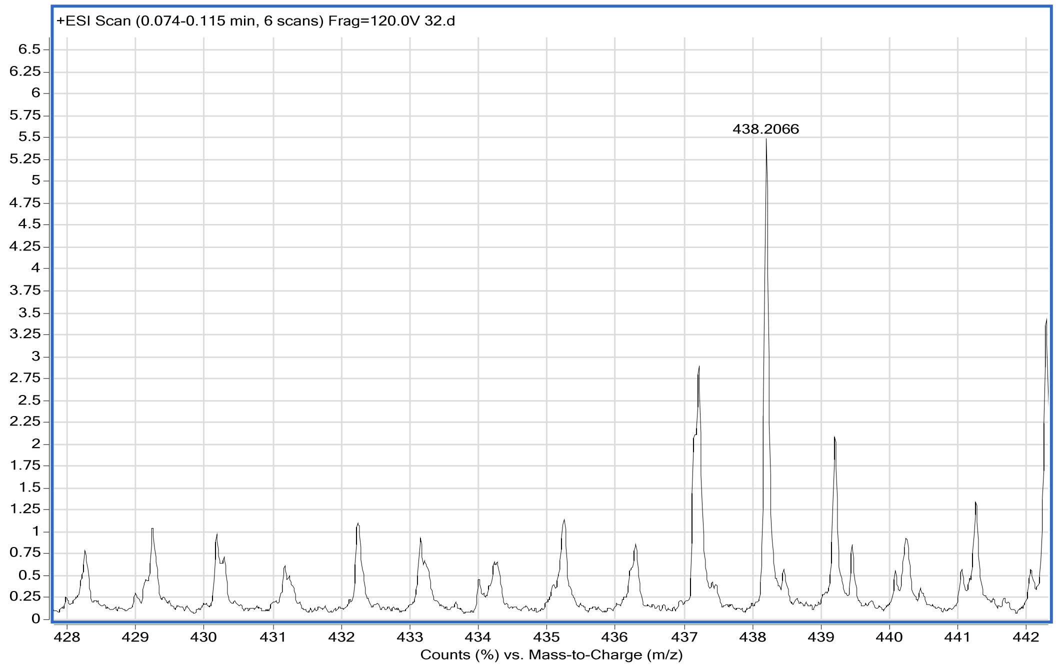

3-methoxy-6,6,11,11-tetramethyl-6,7,11,12-tetrahydrochromeno[2,3,4-gh]phenanthridine-4,13(5H,10H)-dione (**3k**), HRMS (ESI) m/z: $[M+H]^+$ calcd for $C_{24}H_{26}NO_4^+$ 392.1856; found 392.1857.

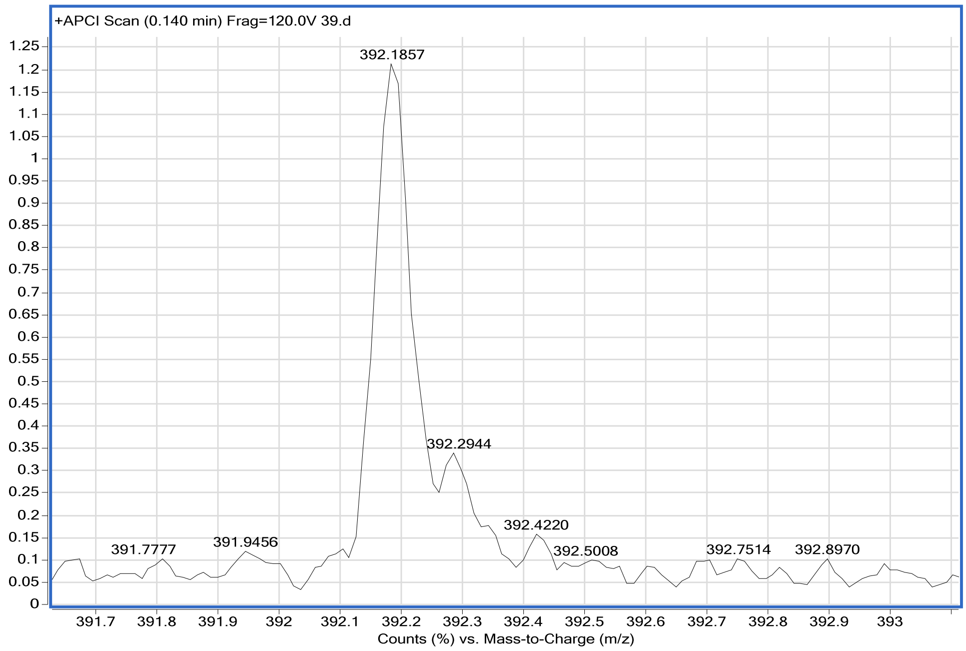

3-fluoro-6,6,11,11-tetramethyl-6,7,11,12-tetrahydrochromeno[2,3,4-gh]phenanthridine-4,13(5H,10H)-dione (**3l**), HRMS (ESI) m/z: $[M+H]^+$ calcd for $C_{23}H_{23}FNO_3^+$ 380.1656; found 380.1658.

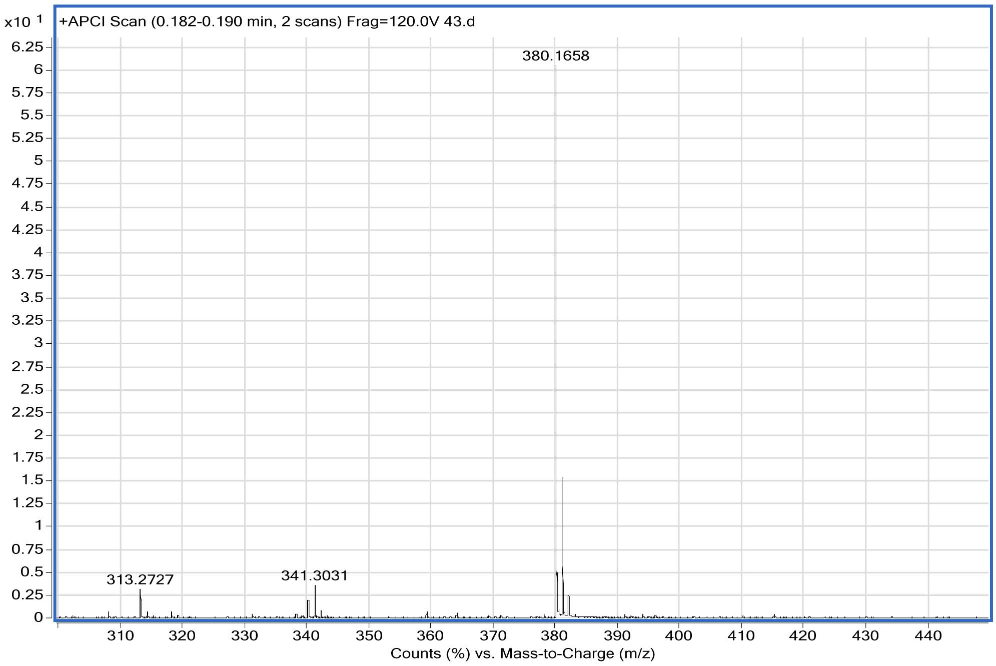

6,11-dimethyl-6,7,11,12-tetrahydrochromeno[2,3,4-gh]phenanthridine-4,13(5H,10H)-dione (**3m**), HRMS (ESI) m/z: $[M+H]^+$ calcd for $C_{21}H_{20}NO_3^+$ 334.1438; found 334.1447.

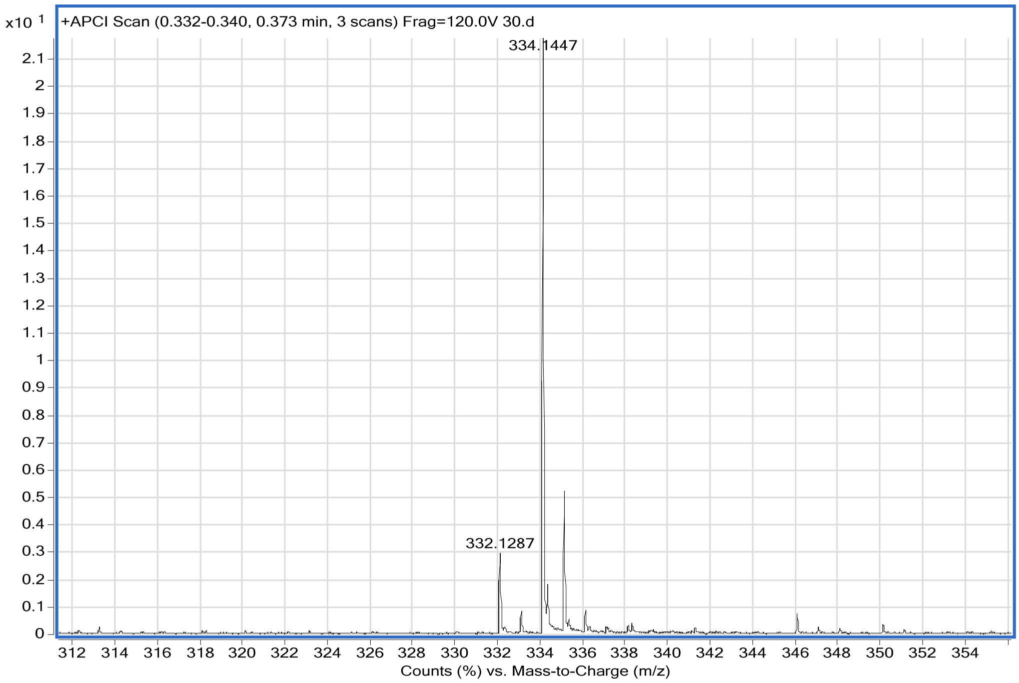

6,7,11,12-tetrahydrochromeno[2,3,4-gh]phenanthridine-4,13(5H,10H)-dione (**3n**), HRMS (ESI) m/z: $[M+H]^+$ calcd for $C_{19}H_{16}NO_3^+$ 306.1125; found 306.1134.

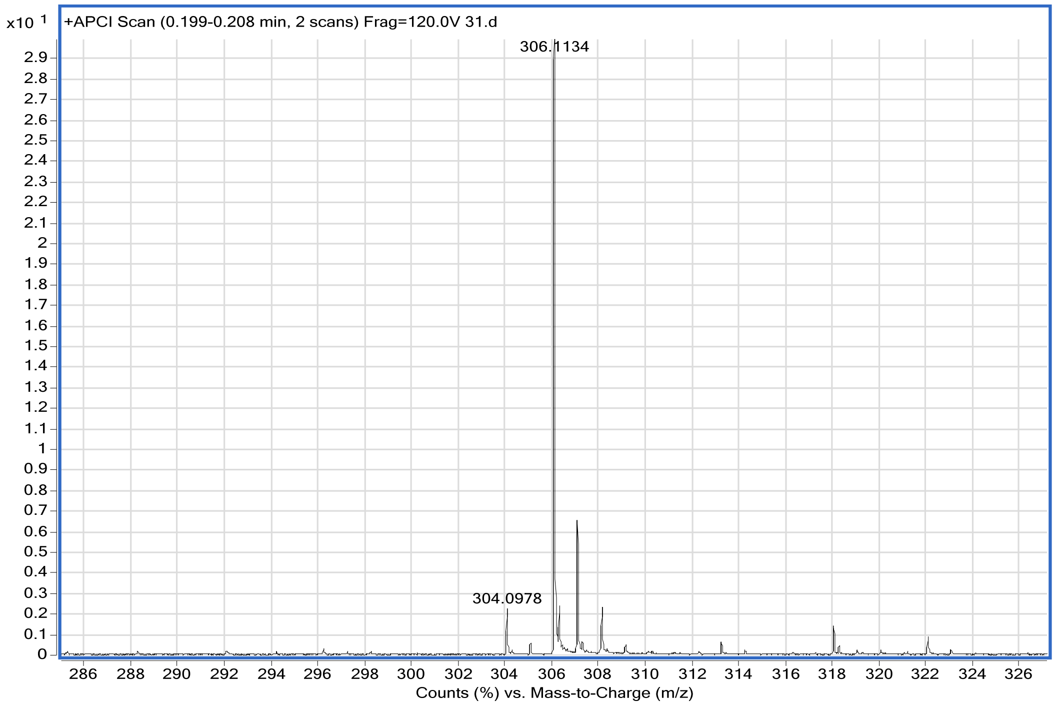

2-methoxy-6,7,11,12-tetrahydrochromeno[2,3,4-gh]phenanthridine-4,13(5H,10H)-dione (**3o**), HRMS (ESI) m/z: $[M+H]^+$ calcd for $C_{20}H_{18}NO_4^+$ 336.1230; found 336.1232.

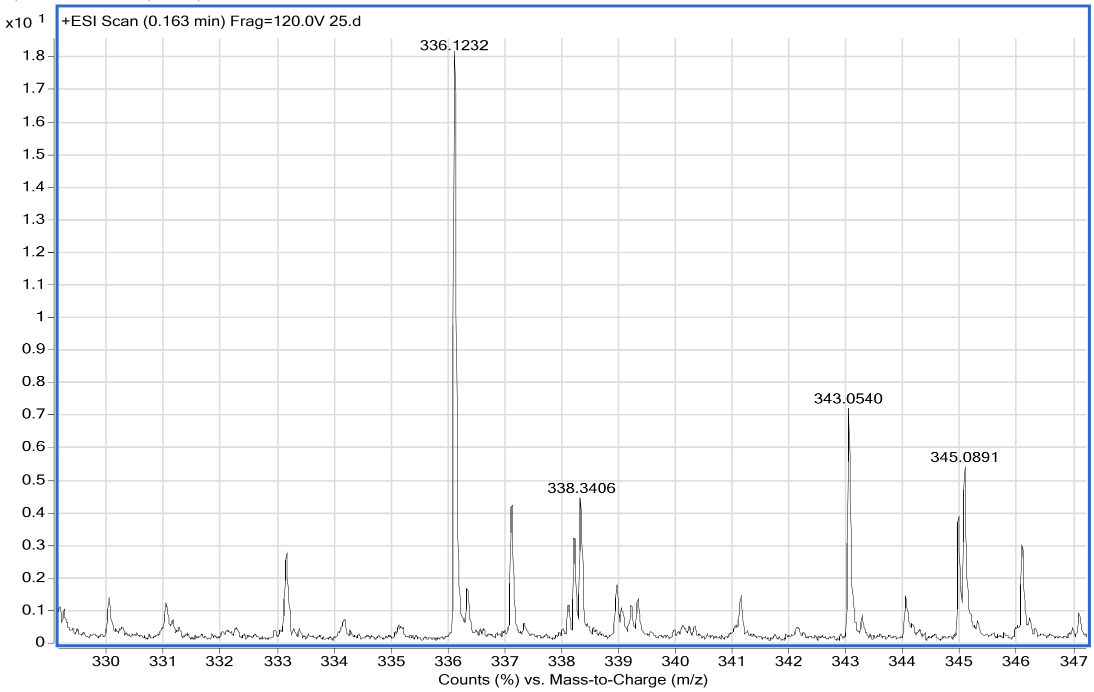

2-methyl-6,7,11,12-tetrahydrochromeno[2,3,4-gh]phenanthridine-4,13(5H,10H)-dione (**3p**), HRMS (ESI) m/z: $[M+H]^+$ calcd for $C_{20}H_{18}NO_3^+$ 320.1281; found 320.1286.

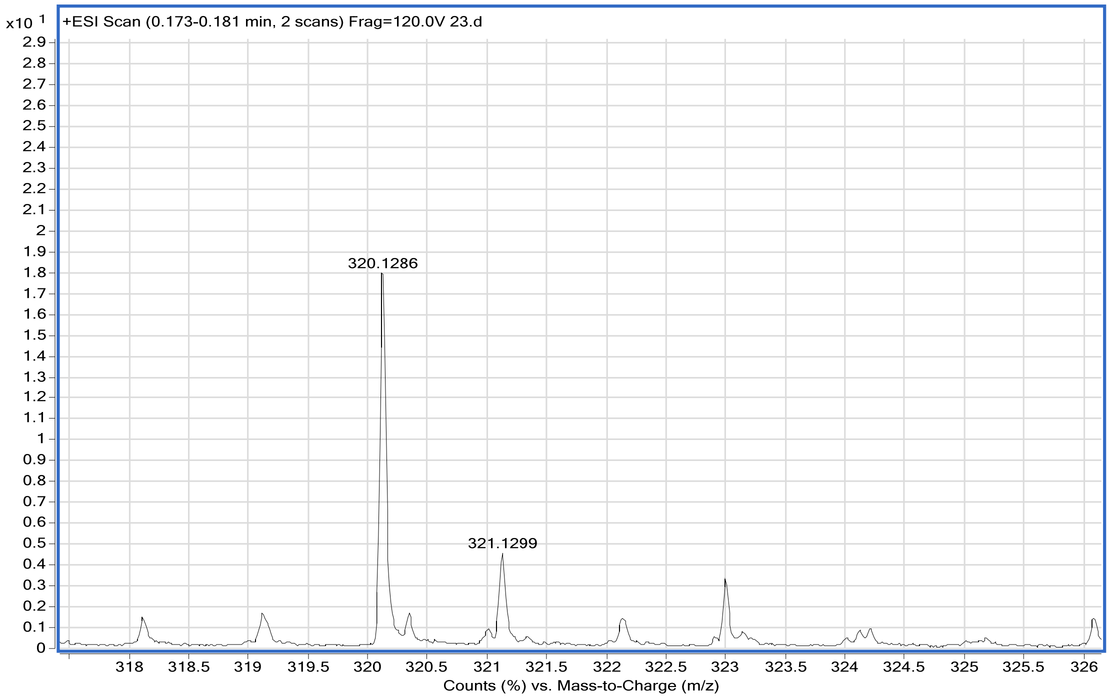

5,6,9,10-tetrahydrocyclopenta[5,6]pyrano[4,3,2-ij]cyclopenta[c]isoquinoline-4,11-Dione (**3q**), HRMS (ESI) m/z: $[M+H]^+$ calcd for $C_{17}H_{12}NO_3^+$ 278.0812; found 278.0816.

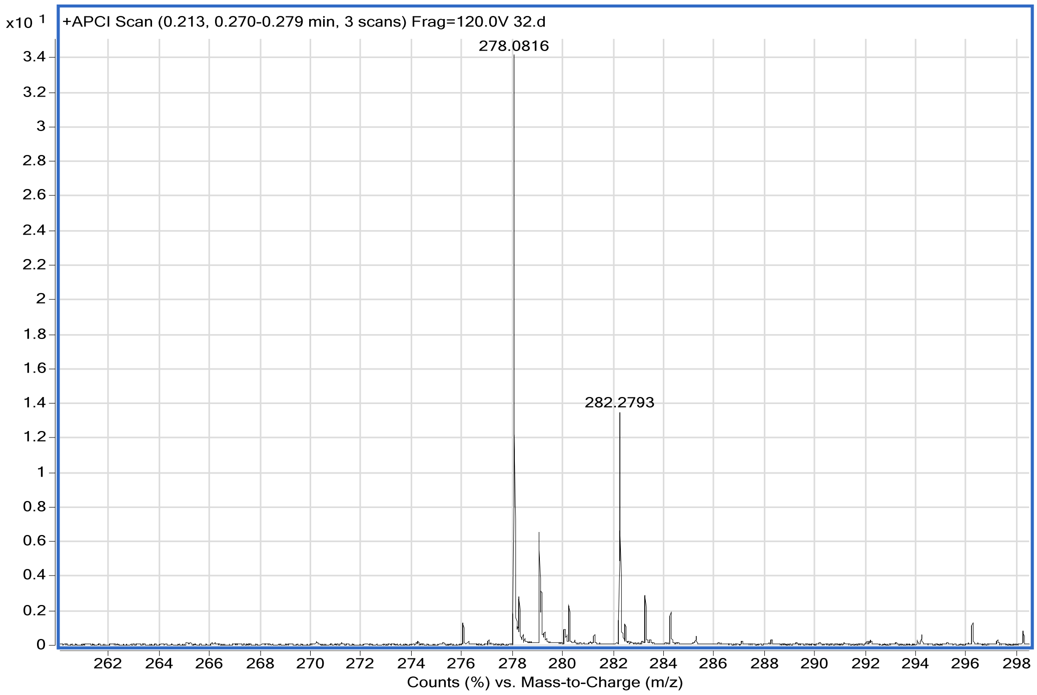

6,11-diphenyl-6,7,11,12-tetrahydrochromeno[2,3,4-gh]phenanthridine-4,13(5H,10H)-dione (**3r**), HRMS (ESI) m/z: $[M+H]^+$ calcd for $C_{31}H_{24}NO_3^+$ 458.1751; found 458.1744.

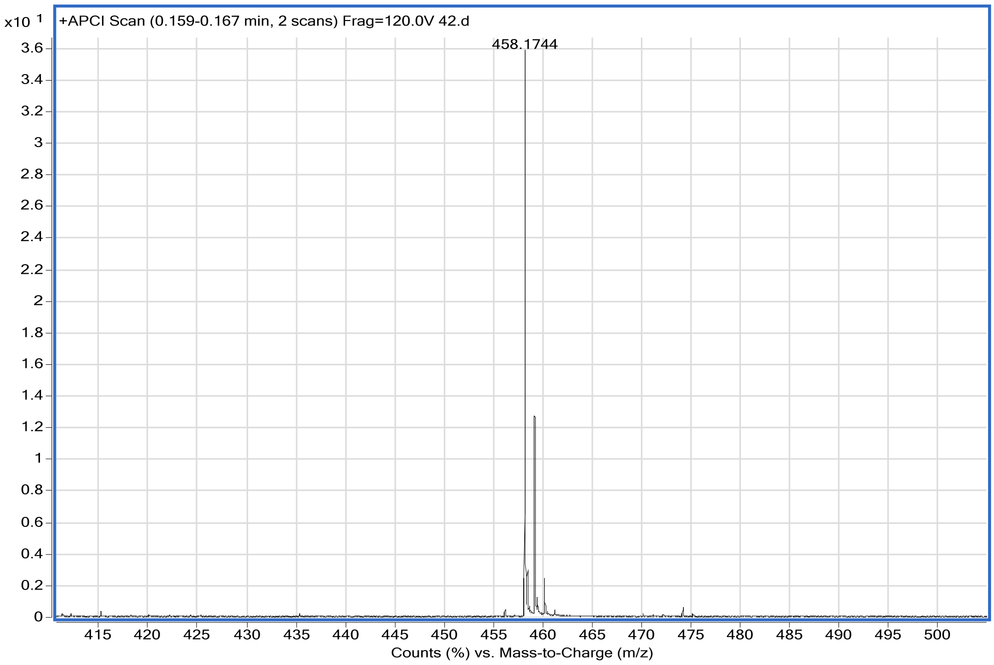

6,11-bis(4-chlorophenyl)-6,7,11,12-tetrahydrochromeno[2,3,4-gh]phenanthridine-4,13(5H,10H)-dione (**3s**),HRMS (ESI) m/z: $[M+H]^+$ calcd for $C_{31}H_{22}ClNO_3^+$ 526.0971; found 526.0978.

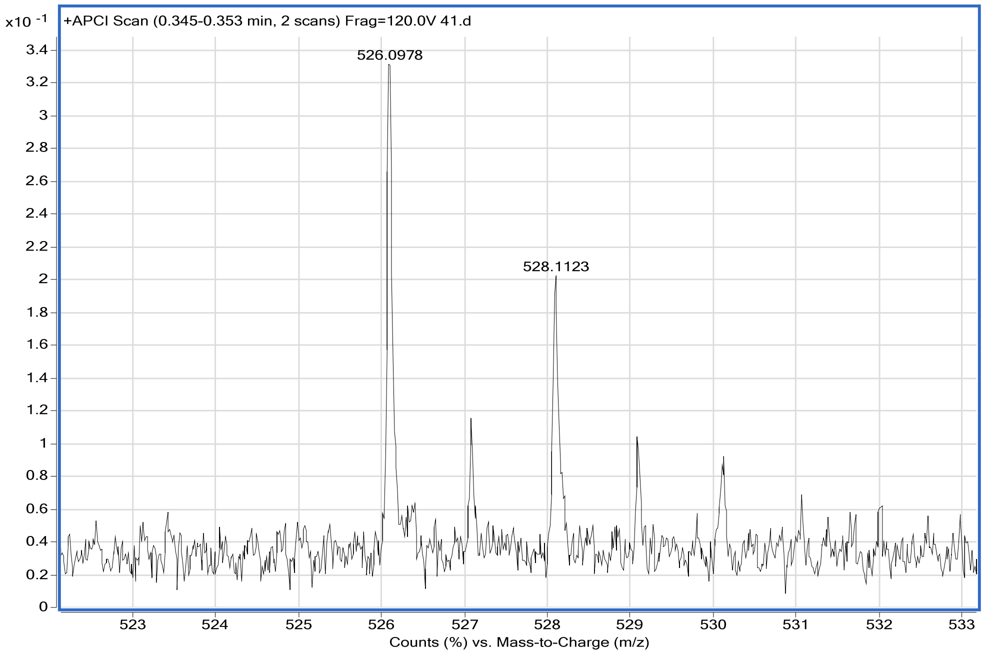

2-butyl-6,11-bis(4-chlorophenyl)-6,7,11,12-tetrahydrochromeno[2,3,4-gh]phenanthridine-4,13(5H,10H)-dione (**3t**), HRMS (ESI) m/z: $[M+H]^+$ calcd for $C_{35}H_{30}ClNO_3^+$ 582.1597; found 582.1604.

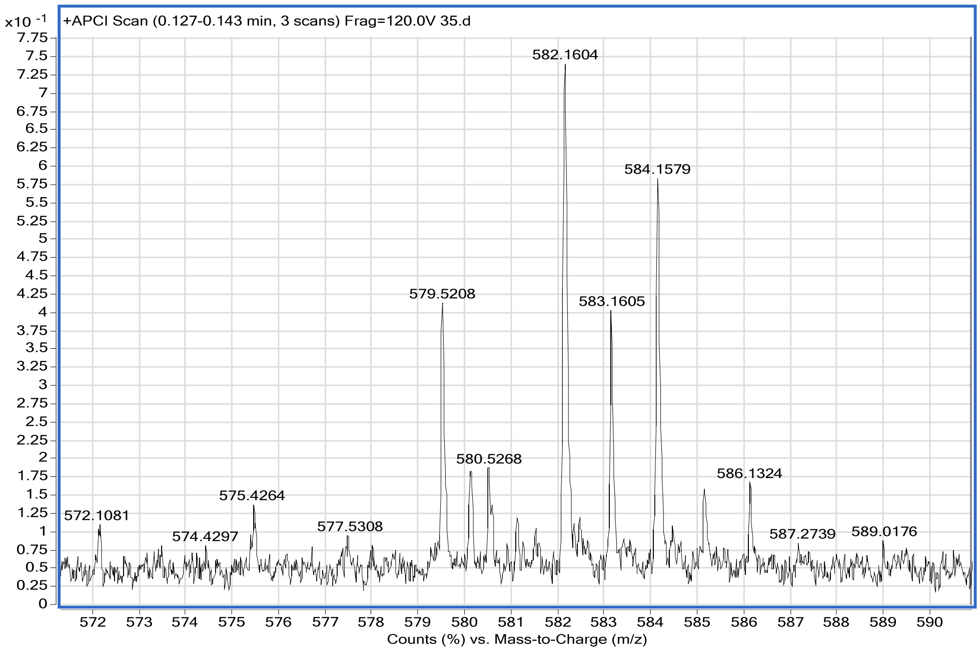

6,6,11,11-tetramethyl-2-(p-tolyl)-6,7,11,12-tetrahydrochromeno[2,3,4-gh] phenanthridine-4,13(5H,10H)-dione (**6**), HRMS (ESI) m/z: [M+H]$^+$ calcd for $C_{30}H_{30}NO_3^+$ 452.2220; found 452.2227.

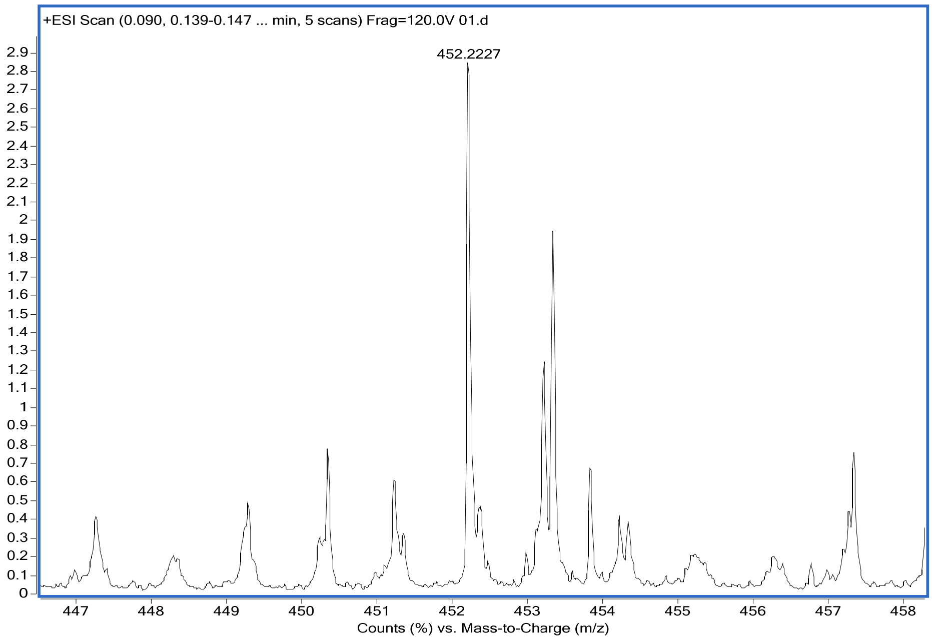

6,6,11,11-tetramethyl-2-(phenyl(p-tolyl)amino)-6,7,11,12-tetrahydrochromeno[2,3,4-gh] phenanthridine-4,13(5H,10H)-dione (**7**), HRMS (ESI) m/z: [M+H]$^+$ calcd for $C_{36}H_{35}N_2O_3^+$ 543.2642; found 543.2648.

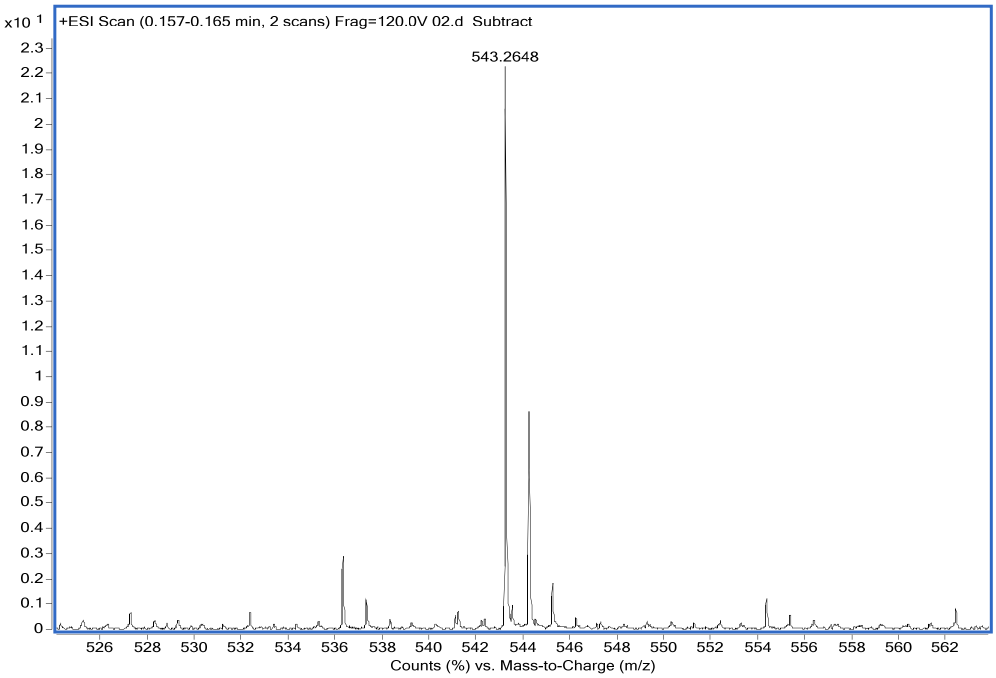

tetrahydrochromeno[2,3,4-gh] phenanthridine-4,13(5H,10H)-dione (**8**), HRMS (ESI) m/z: [M+H]$^+$ calcd for $C_{29}H_{35}BNO_5^+$ 488.2603; found 488.2604.

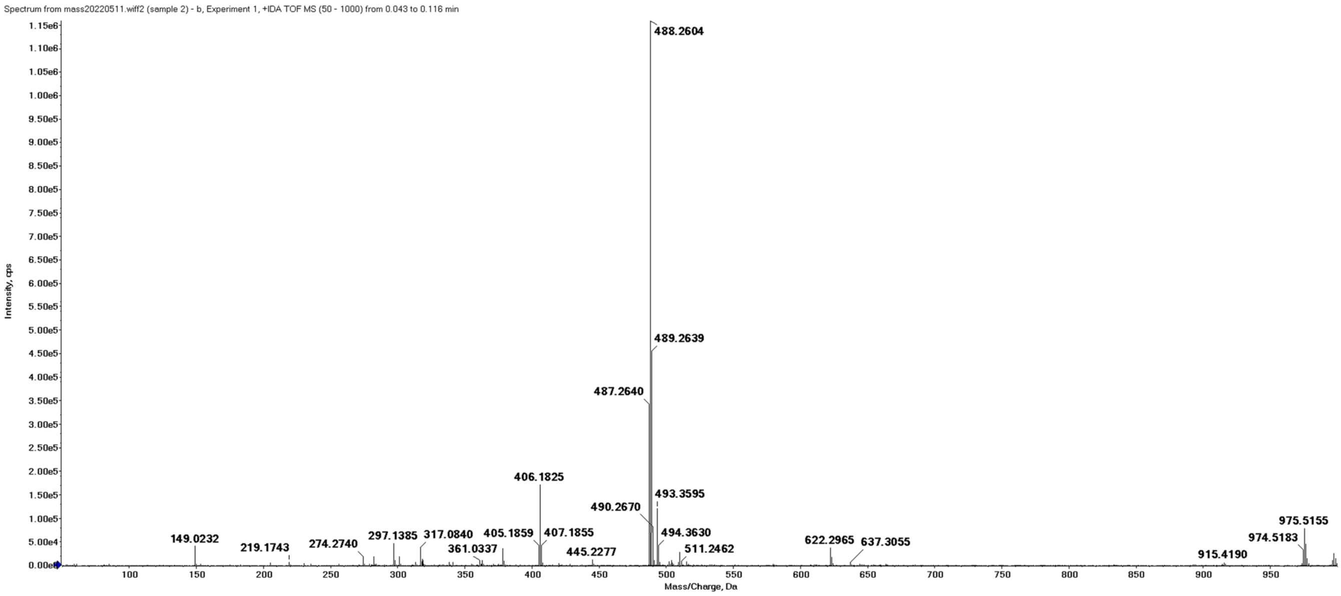

2-bromo-6,6,11,11-tetramethyl-4,5,6,7,10,11,12,13-octahydrochromeno[2,3,4-gh] phenanthridine-4,13-diol (**9**), HRMS (ESI) m/z: [M+H]$^+$ calcd for $C_{23}H_{27}BrNO_3^+$ 444.1169; found 444.1171.

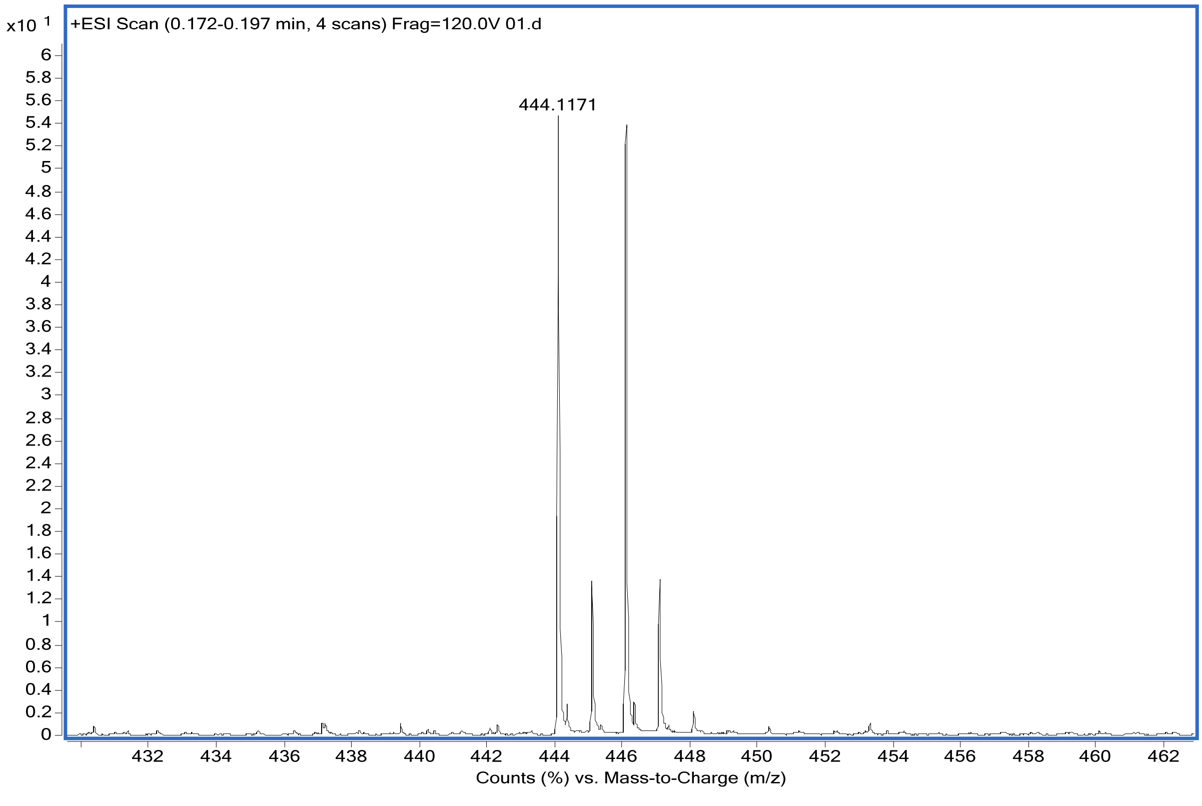

Supplement: Supplementary file 1 [file molecules-30-00149-s001.zip › molecules-3365162-supplementary.pdf]
